# Supplementary figures and images for: Combinatorial CRISPR screen reveals FYN and KDM4 as targets for synergistic drug combination for treating triple negative breast cancer
Source: eLife. 2025 Apr 17;13:RP93921. doi: 10.7554/eLife.93921 (PMC12005726; doi:10.7554/eLife.93921)

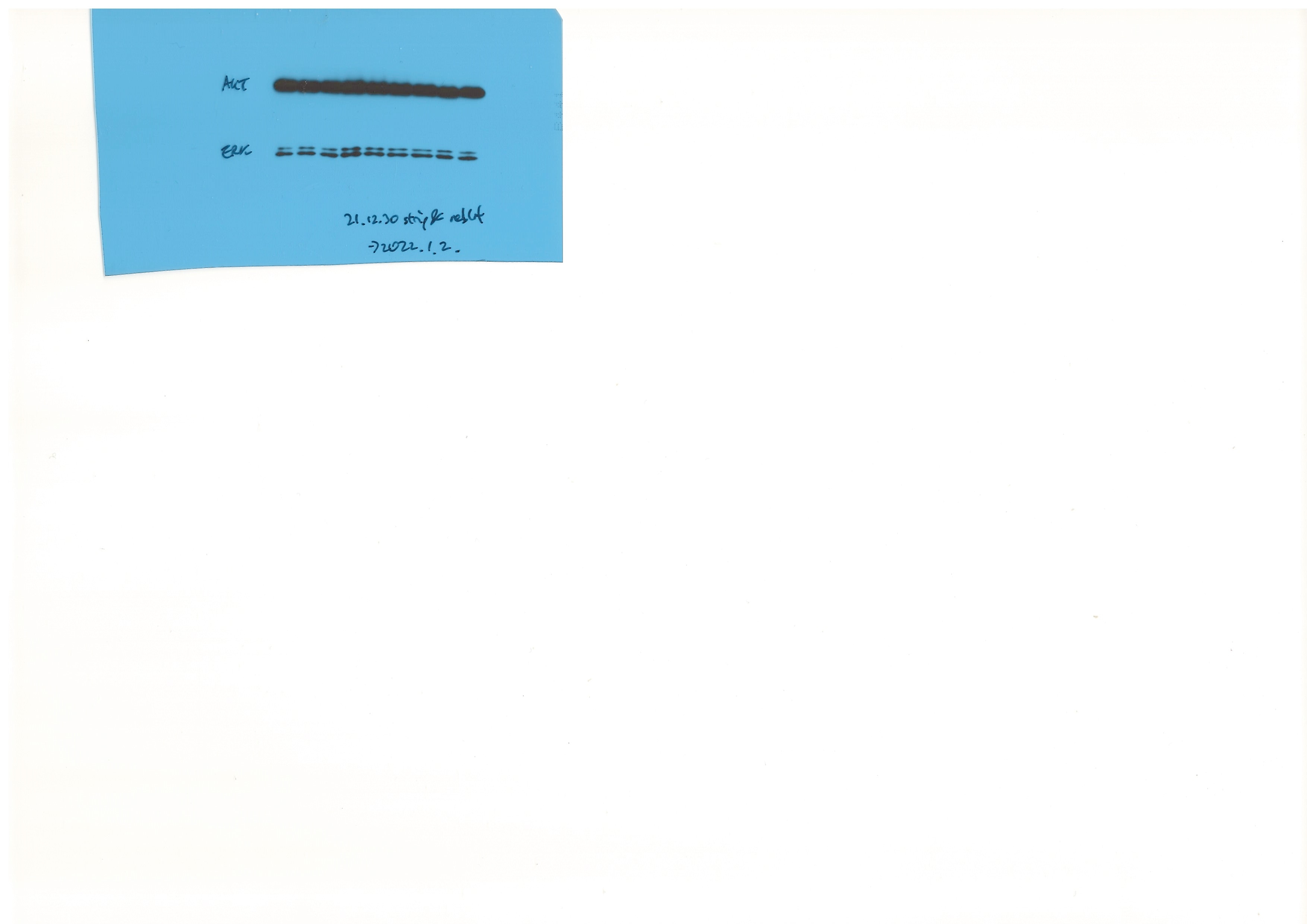

Supplement: Figure 2—source data 1. [file elife-93921-fig2-data1.zip › 2J-ERK AKT.jpg]

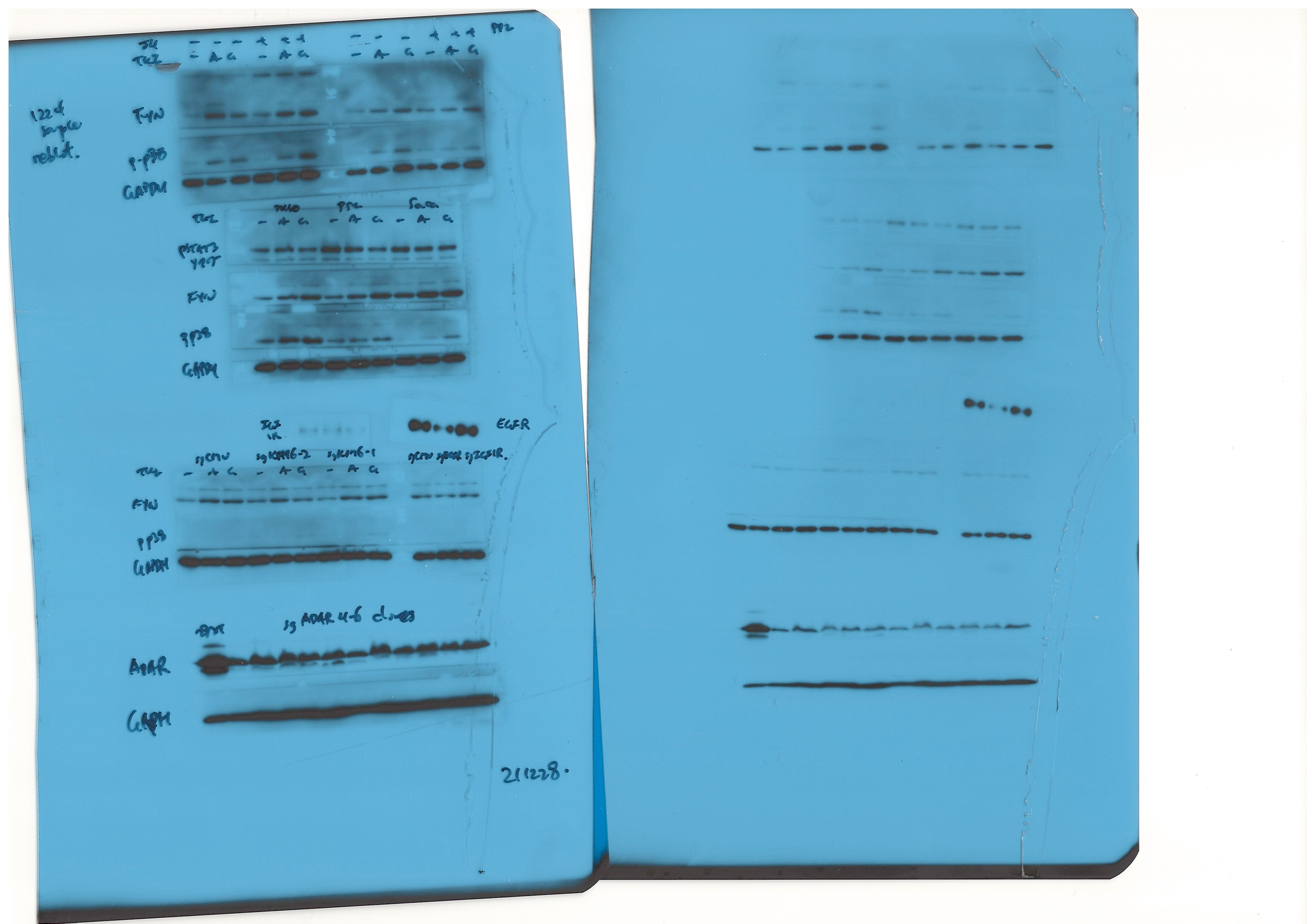

Supplement: Figure 2—source data 1. [file elife-93921-fig2-data1.zip › 2J-FYN pSTAT3 pp38 GAPDH.jpg]

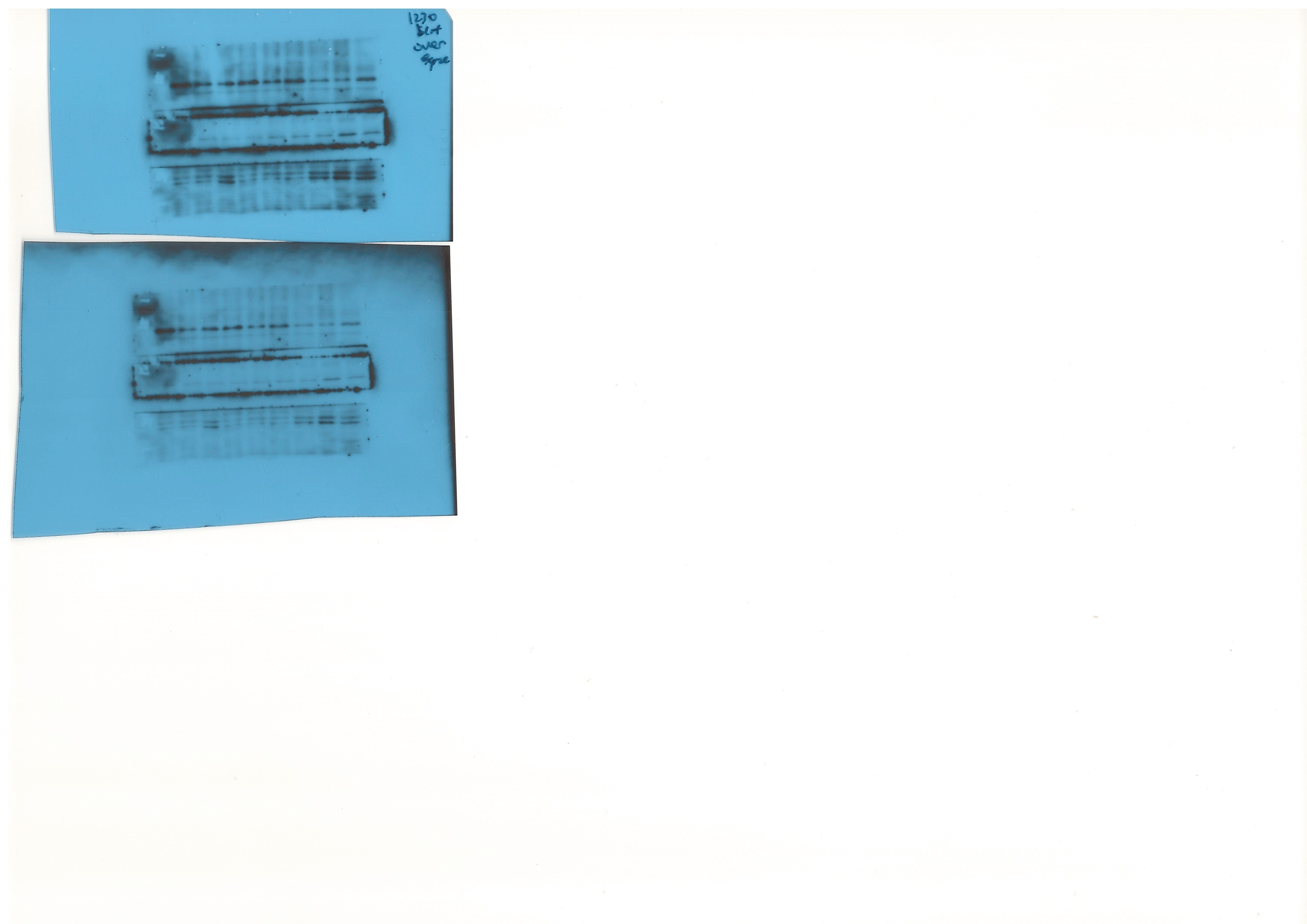

Supplement: Figure 2—source data 1. [file elife-93921-fig2-data1.zip › 2J-SRC pAKT pERK.jpg]

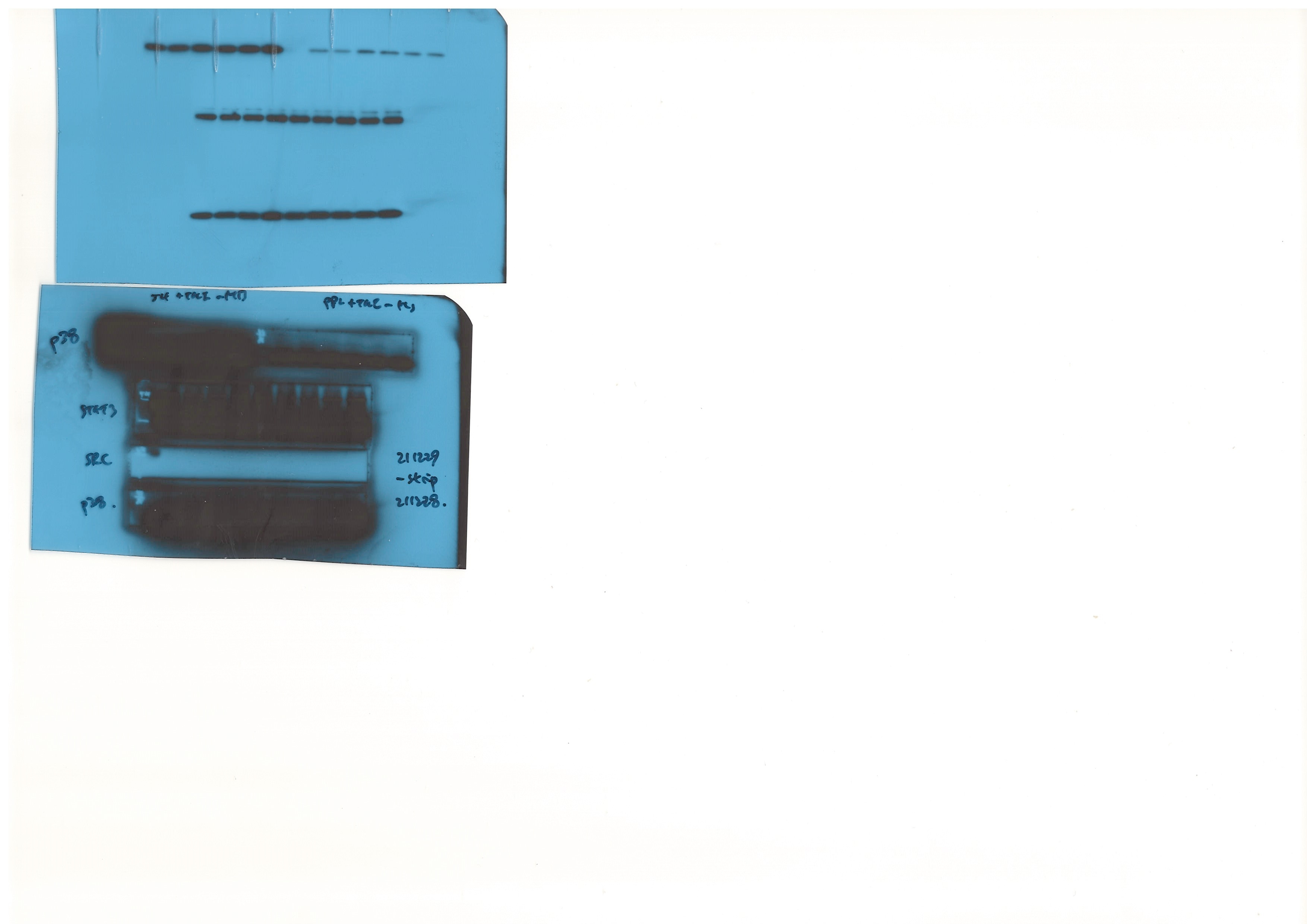

Supplement: Figure 2—source data 1. [file elife-93921-fig2-data1.zip › 2J-STAT3 p38.jpg]

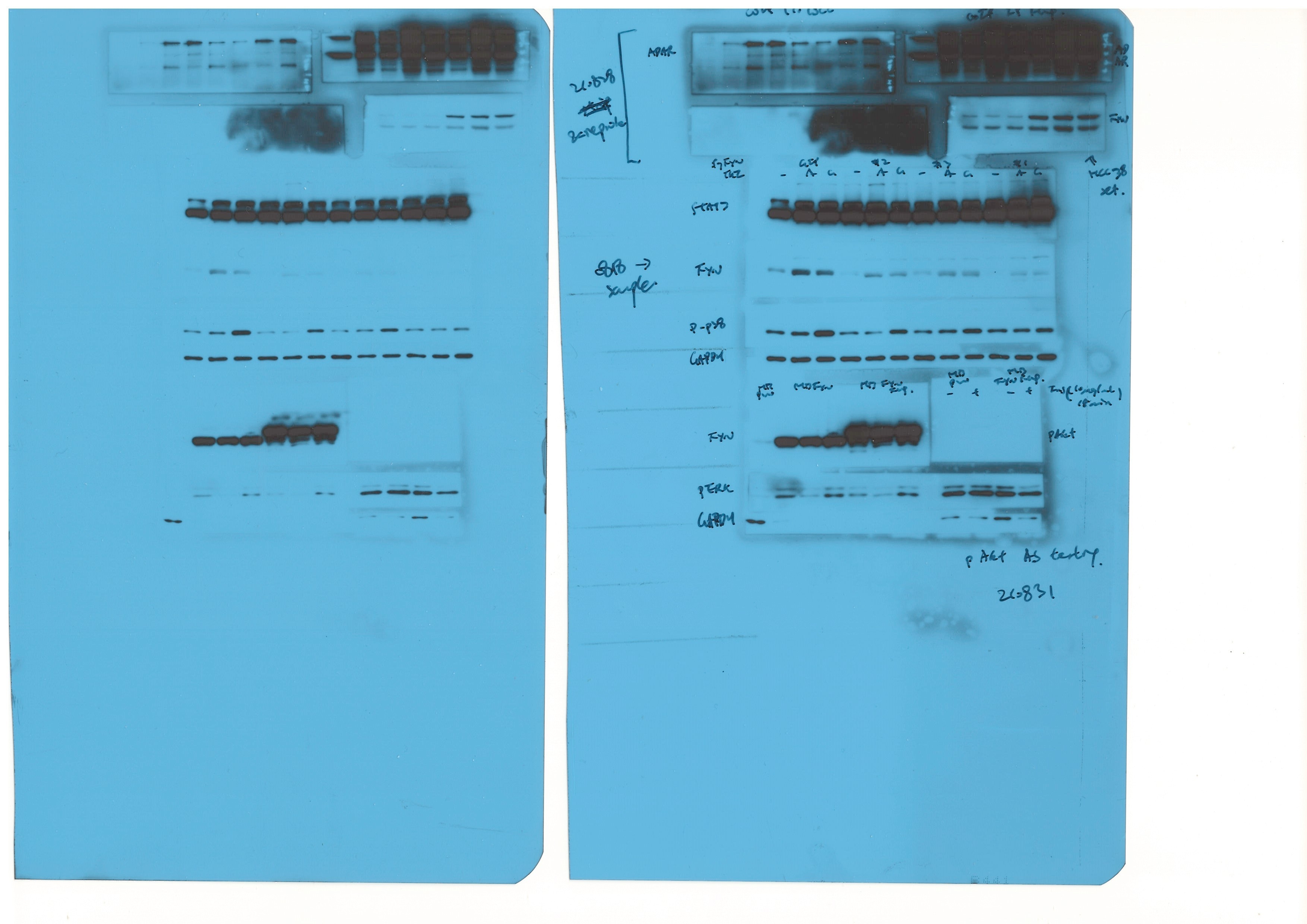

Supplement: Figure 2—source data 1. [file elife-93921-fig2-data1.zip › 2K.jpg]

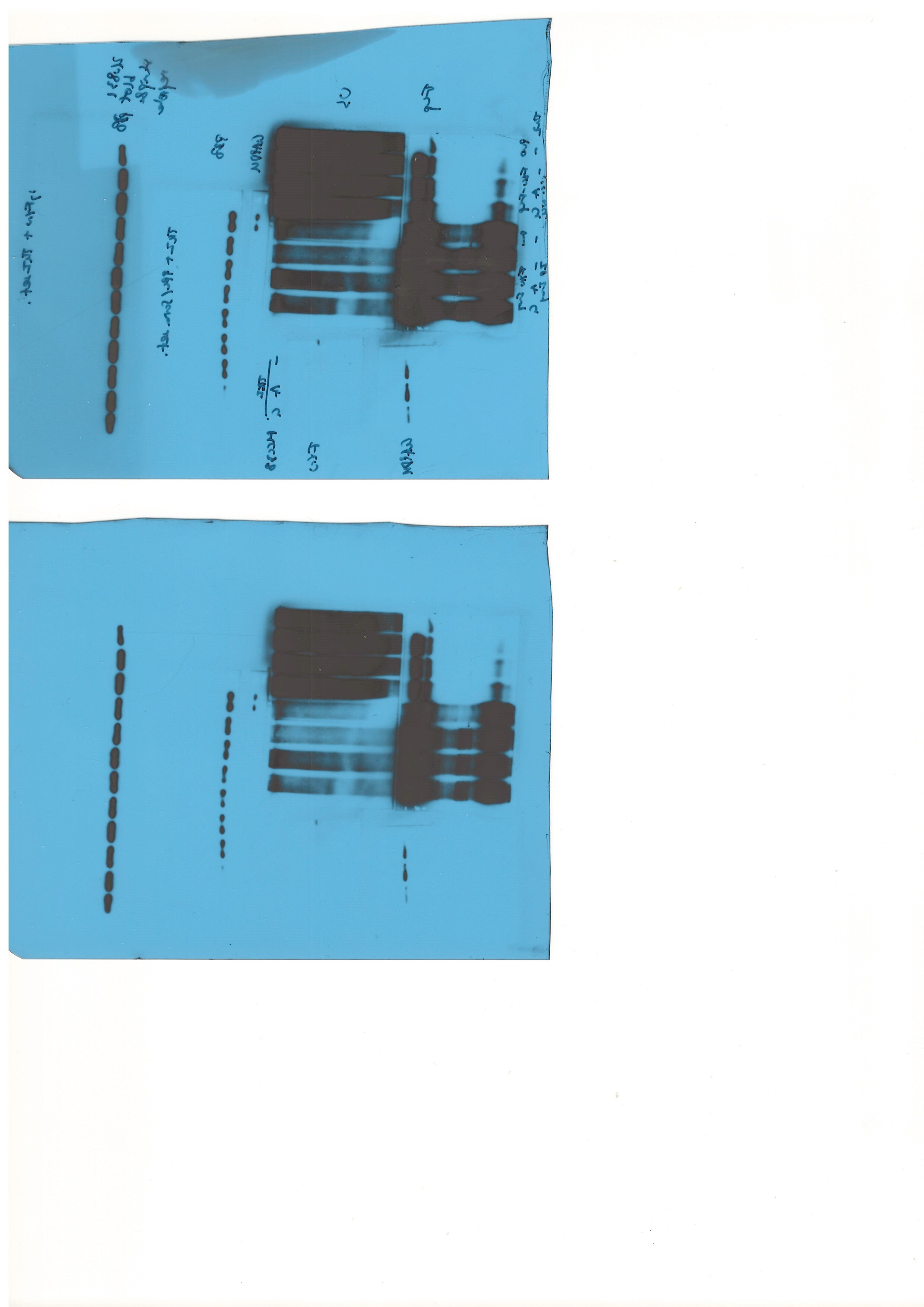

Supplement: Figure 2—source data 1. [file elife-93921-fig2-data1.zip › 2K-p38.jpg]

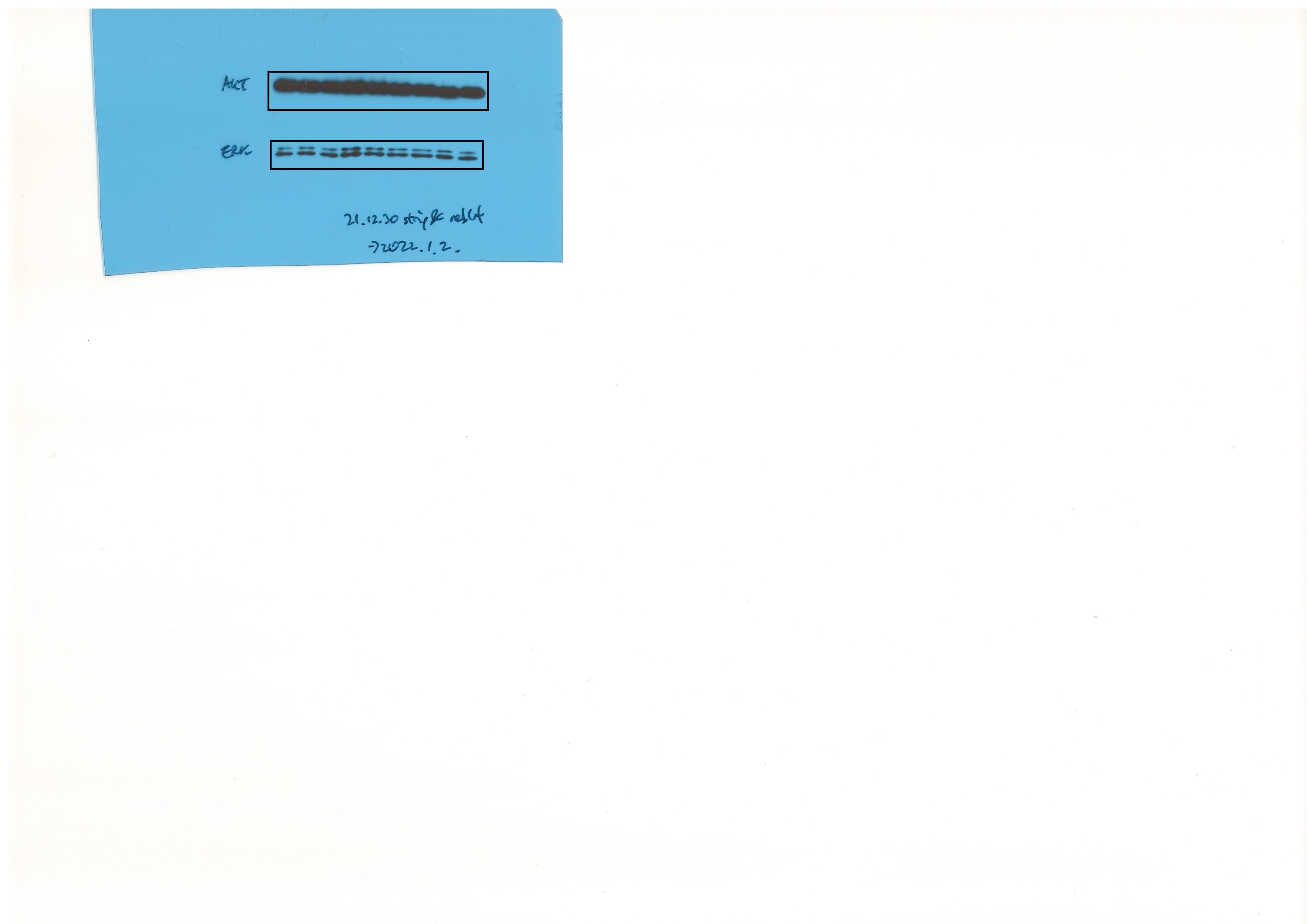

Supplement: Figure 2—source data 2. [file elife-93921-fig2-data2.zip › 2J-ERK AKT.jpg]

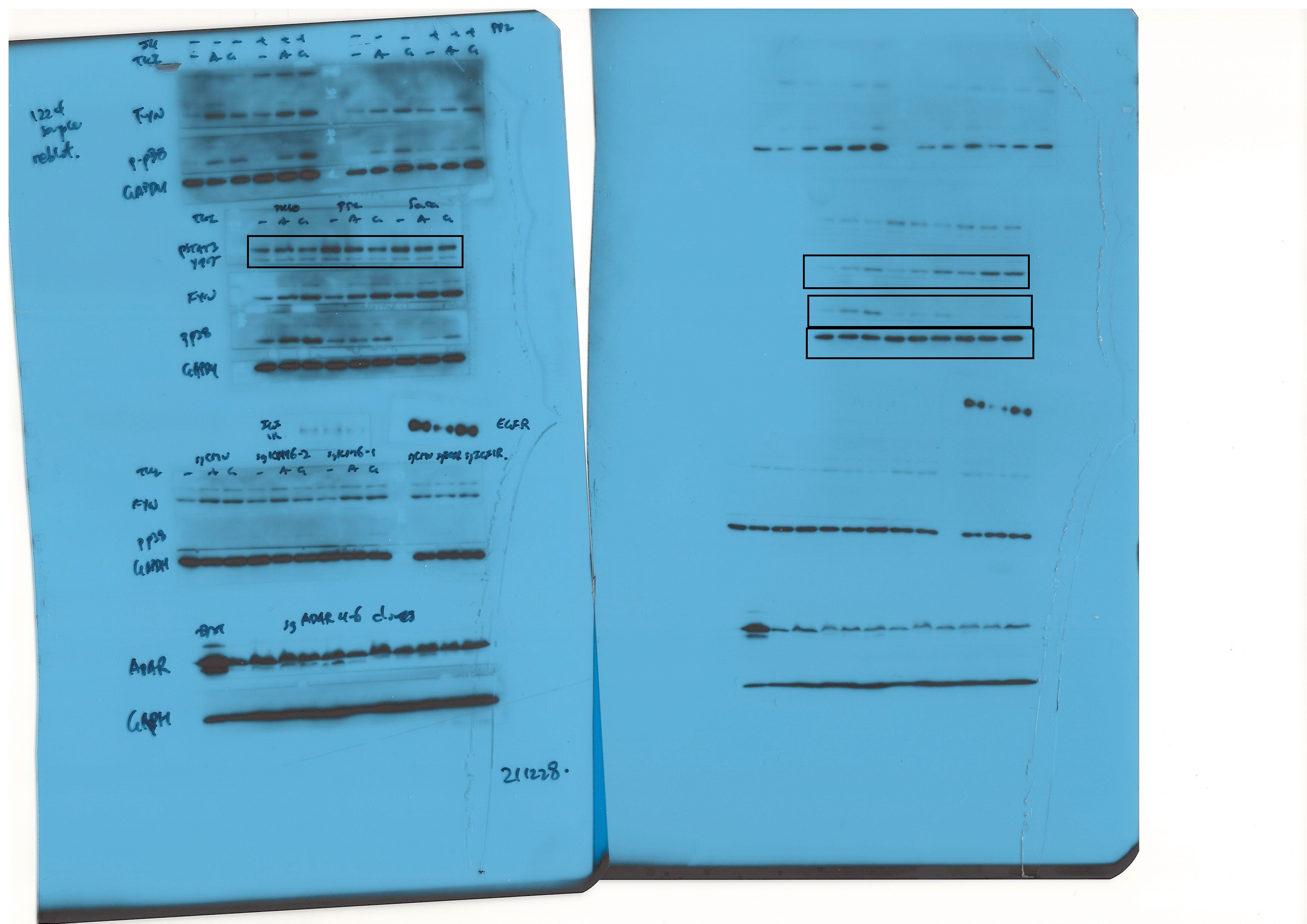

Supplement: Figure 2—source data 2. [file elife-93921-fig2-data2.zip › 2J-FYN pSTAT3 pp38 GAPDH.jpg]

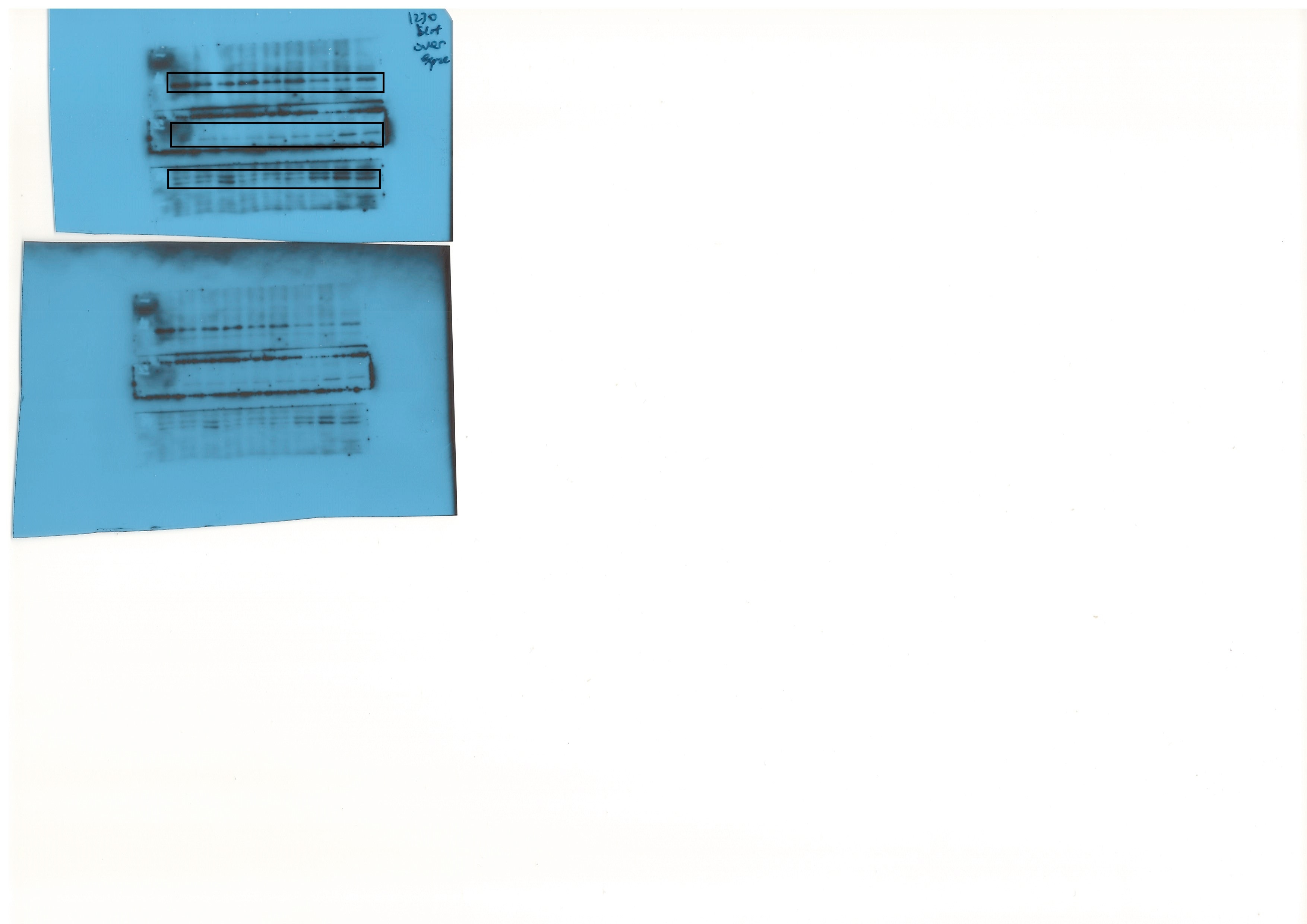

Supplement: Figure 2—source data 2. [file elife-93921-fig2-data2.zip › 2J-SRC pAKT pERK.jpg]

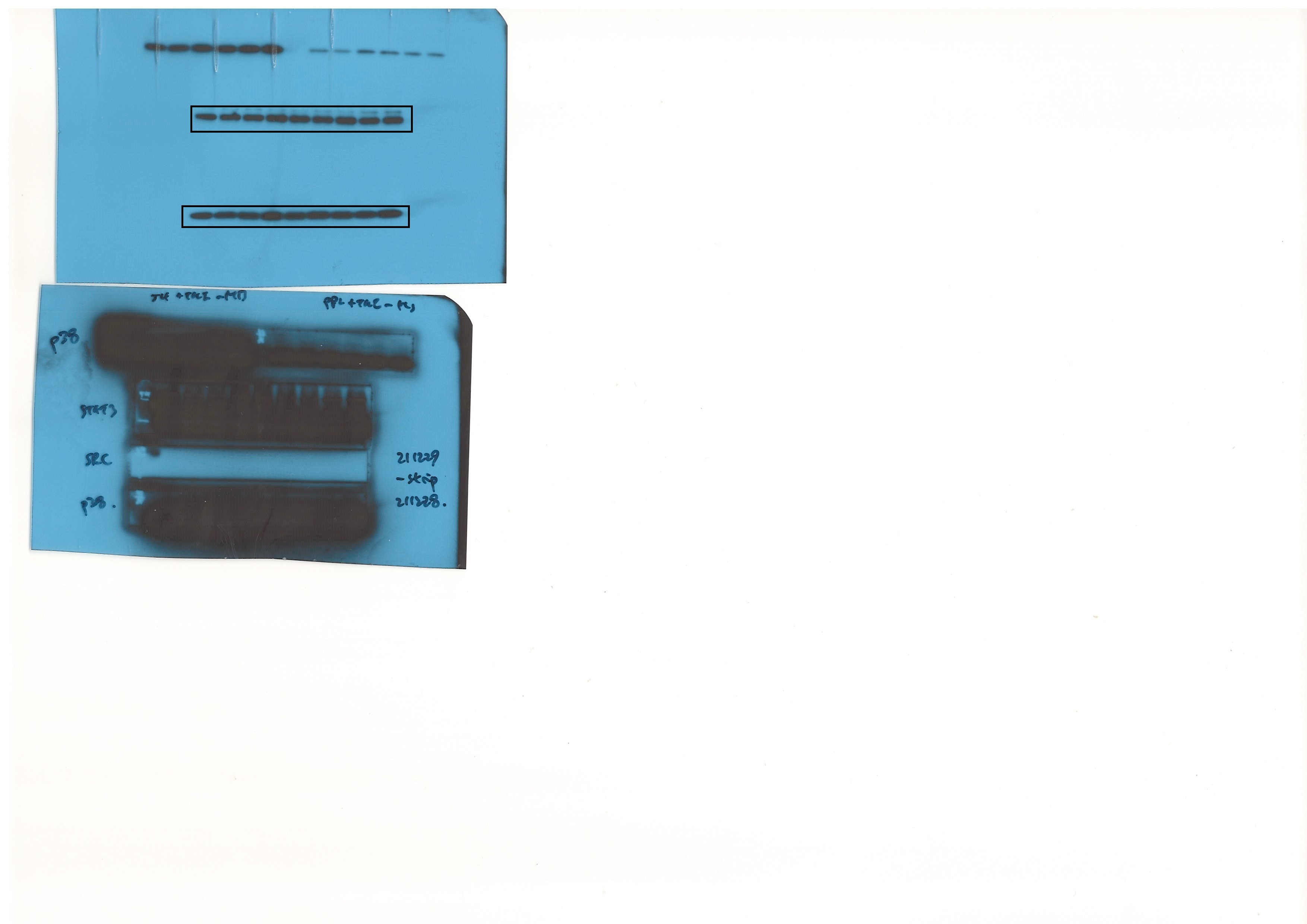

Supplement: Figure 2—source data 2. [file elife-93921-fig2-data2.zip › 2J-STAT3 p38.jpg]

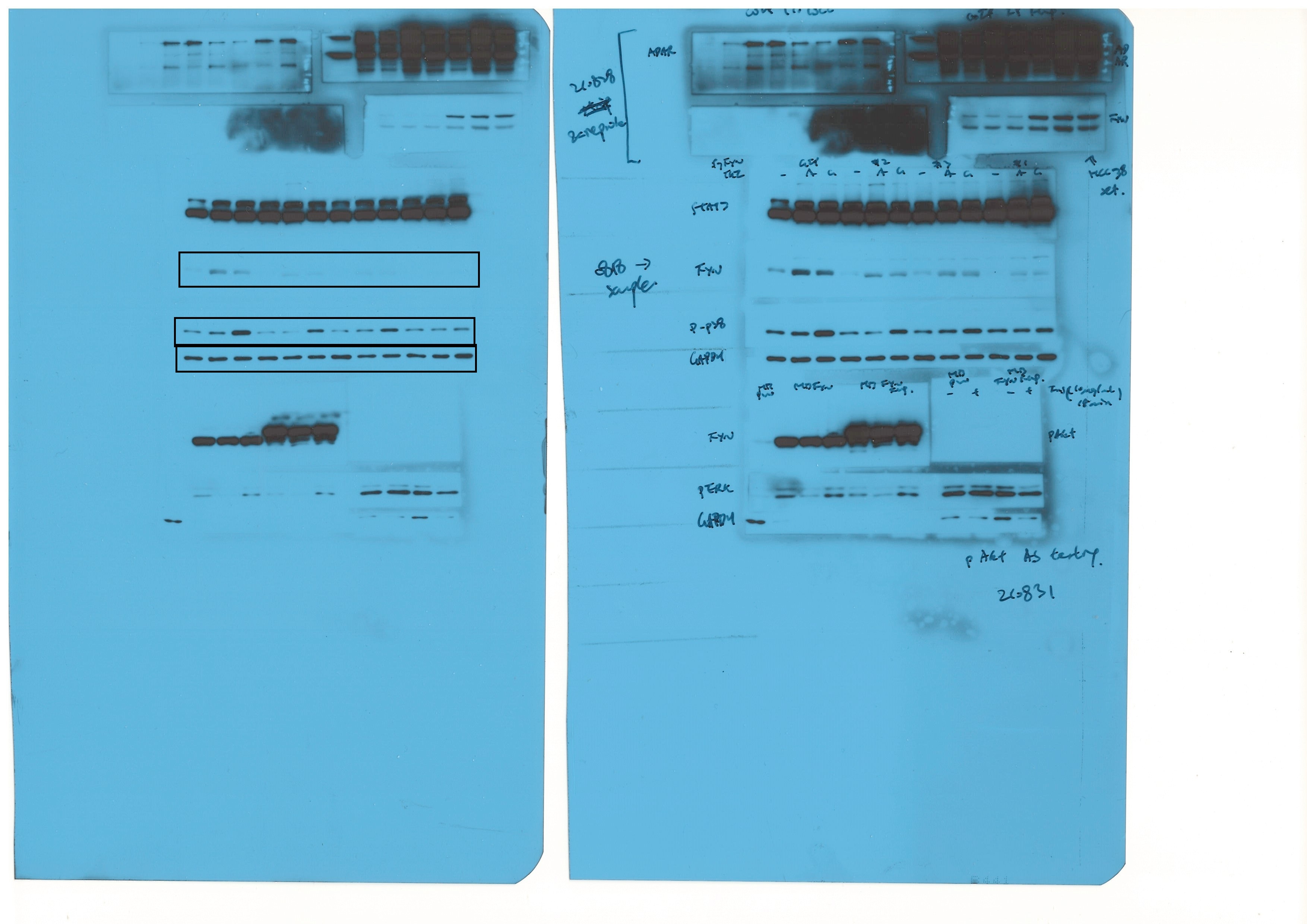

Supplement: Figure 2—source data 2. [file elife-93921-fig2-data2.zip › 2K.jpg]

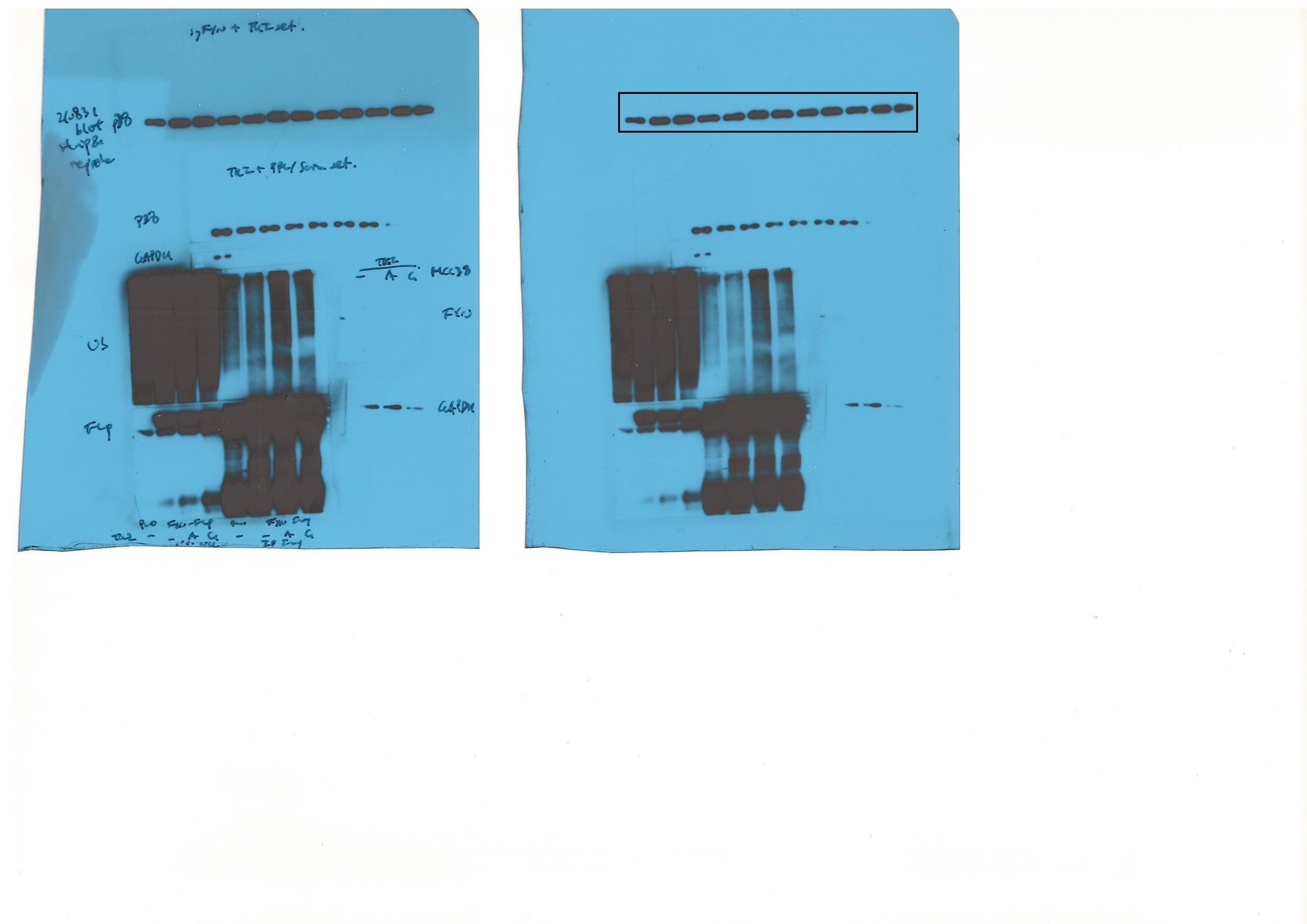

Supplement: Figure 2—source data 2. [file elife-93921-fig2-data2.zip › 2K-p38.jpg]

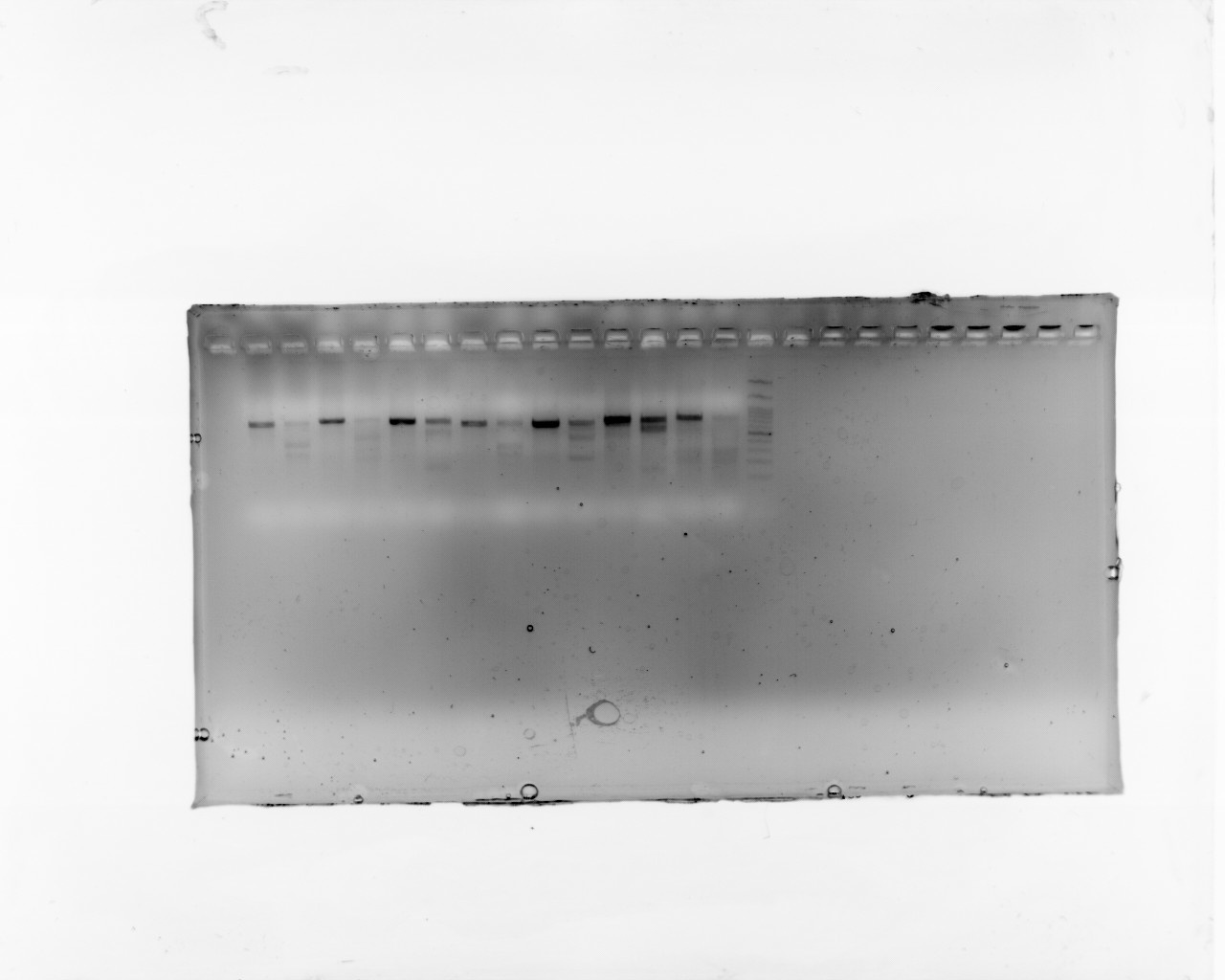

Supplement: Figure 2—figure supplement 1—source data 1. [file elife-93921-fig2-figsupp1-data1.zip › S2B-sgABL2-T7 assay-original.jpg]

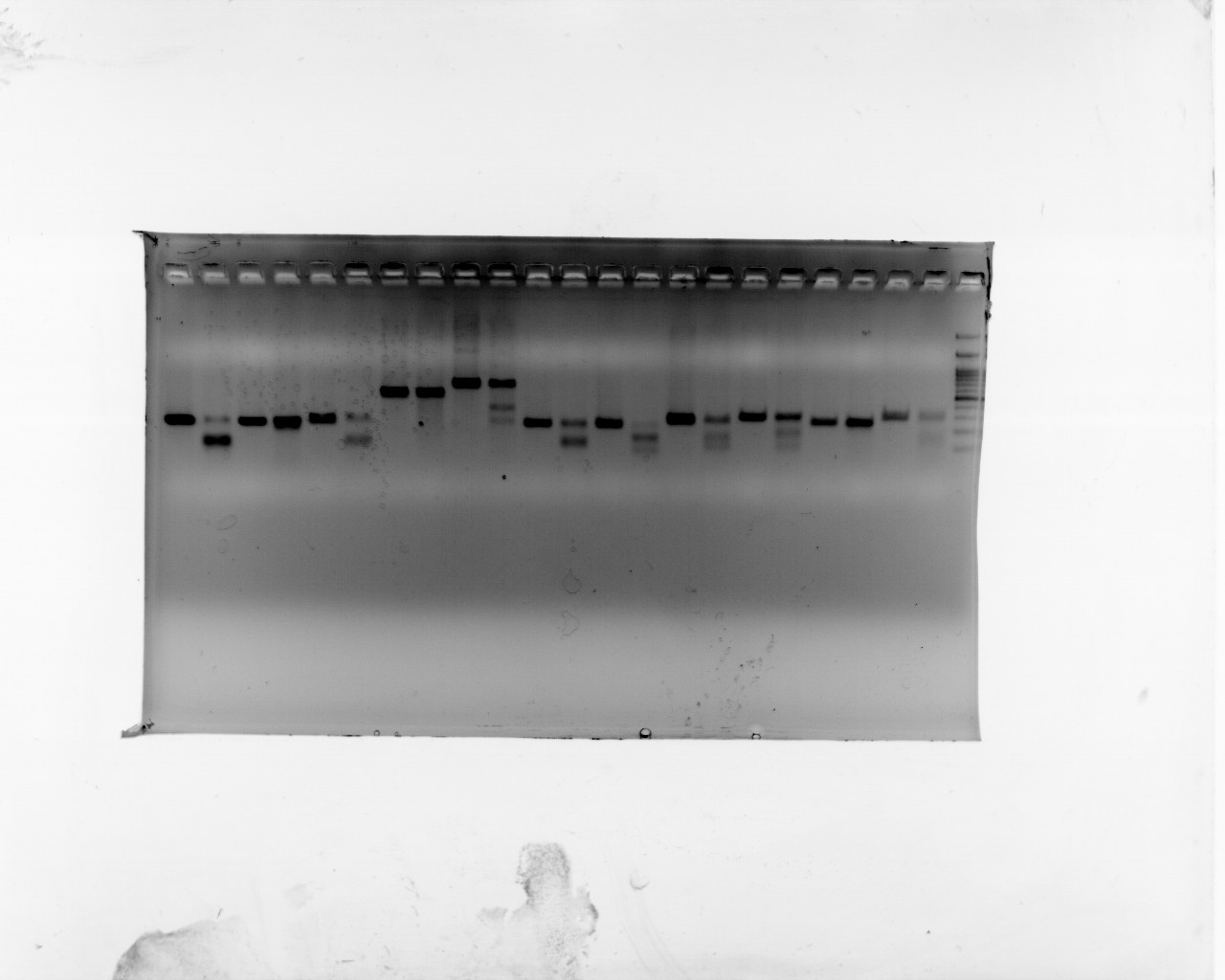

Supplement: Figure 2—figure supplement 1—source data 1. [file elife-93921-fig2-figsupp1-data1.zip › S2B-sgFGFR2 YES EGFR IGF1R T7 assay-original.tif]

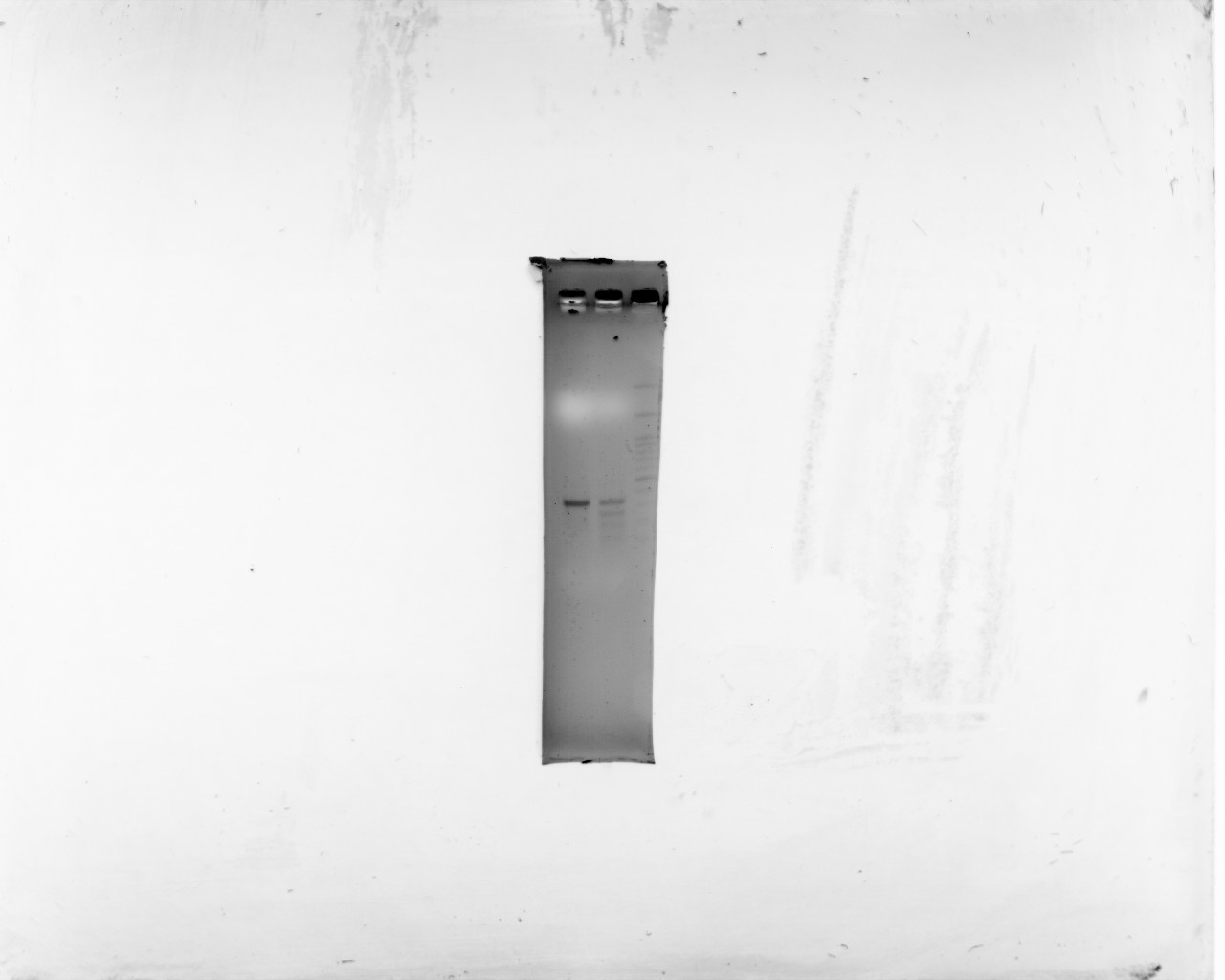

Supplement: Figure 2—figure supplement 1—source data 1. [file elife-93921-fig2-figsupp1-data1.zip › S2B-sgPTK6 T7 assay-original.tif]

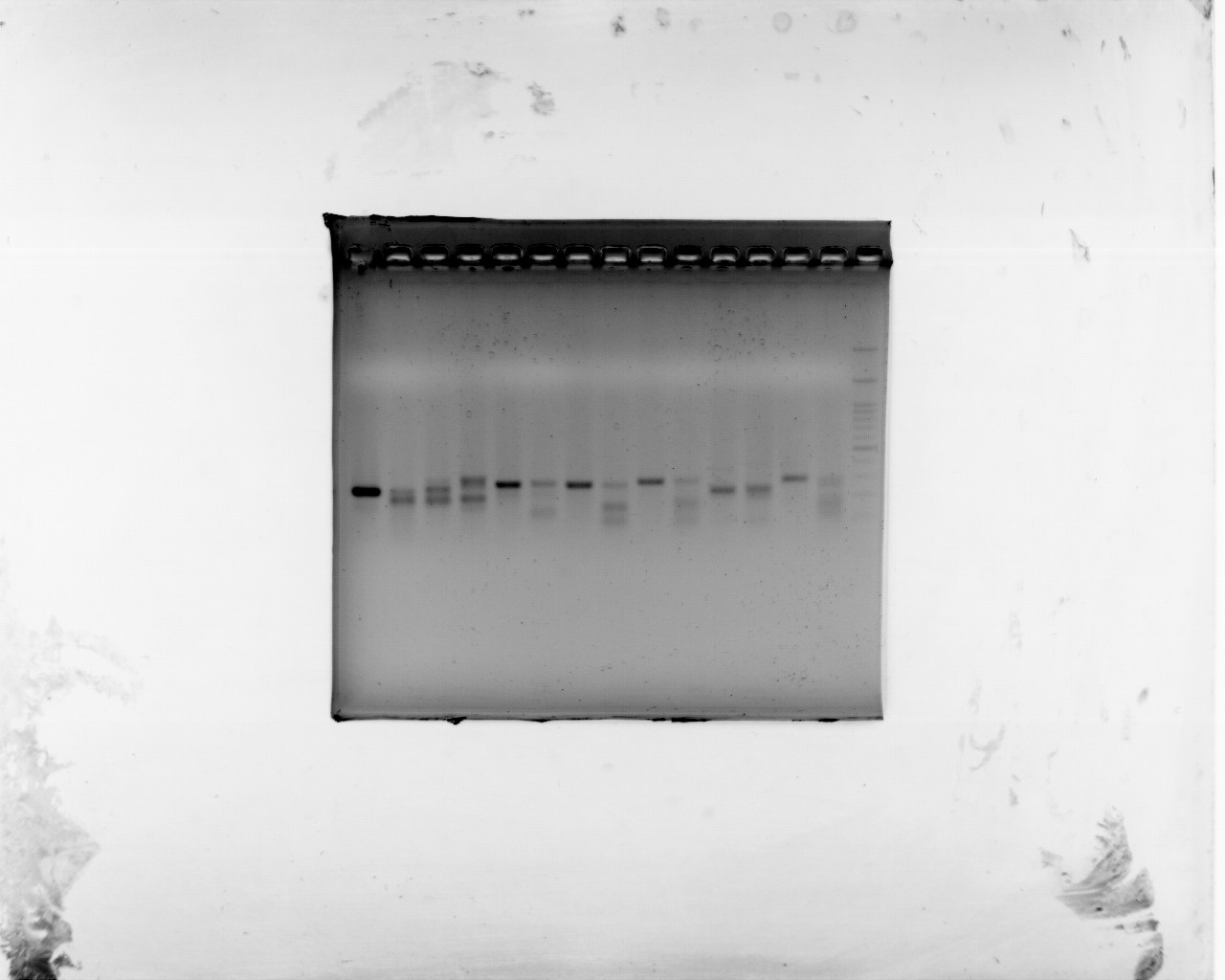

Supplement: Figure 2—figure supplement 1—source data 1. [file elife-93921-fig2-figsupp1-data1.zip › S2B-sgSRC-T7 assay-original.tif]

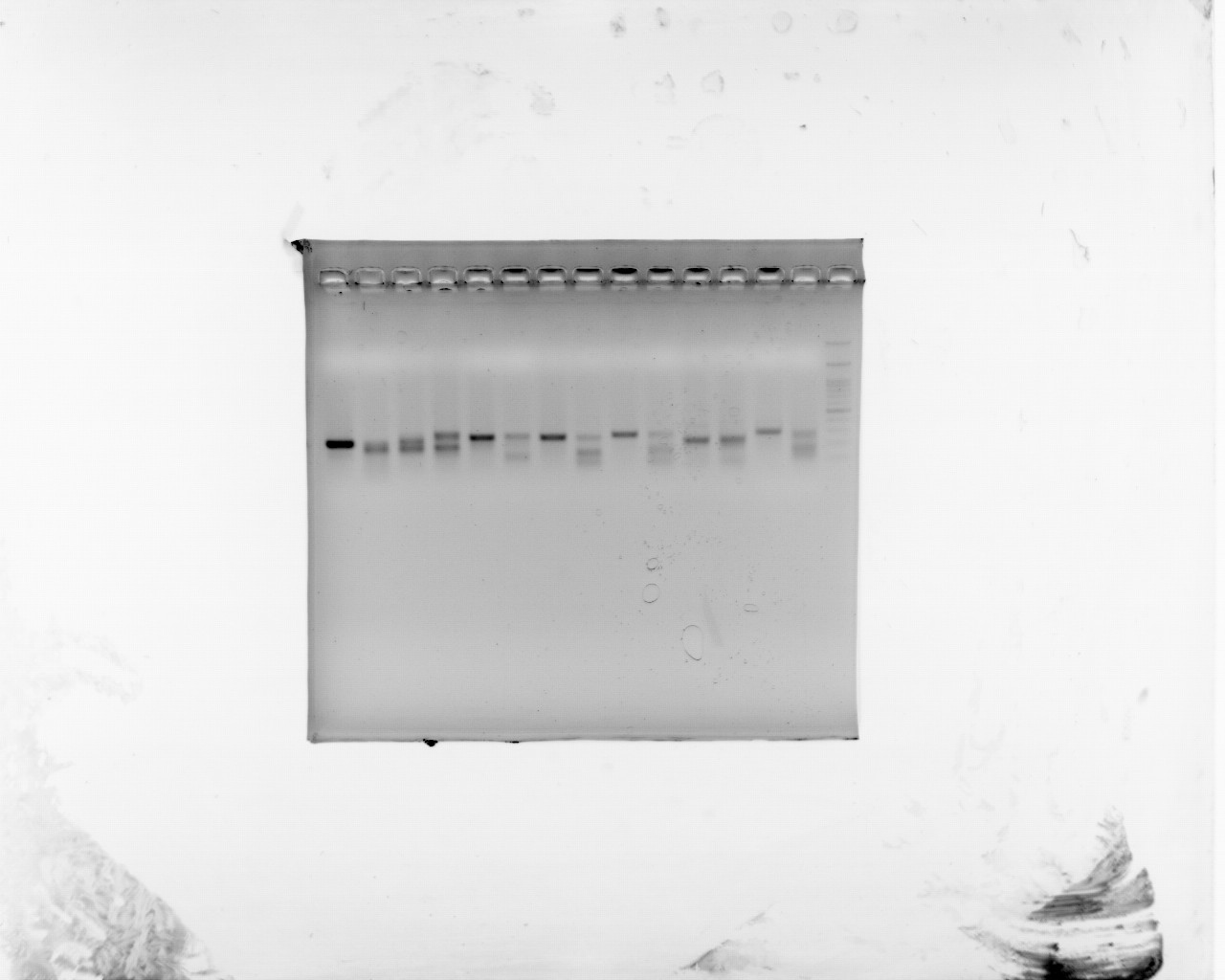

Supplement: Figure 2—figure supplement 1—source data 1. [file elife-93921-fig2-figsupp1-data1.zip › S2B-sgTEK FYN PDGFRA-T7 assay-original.tif]

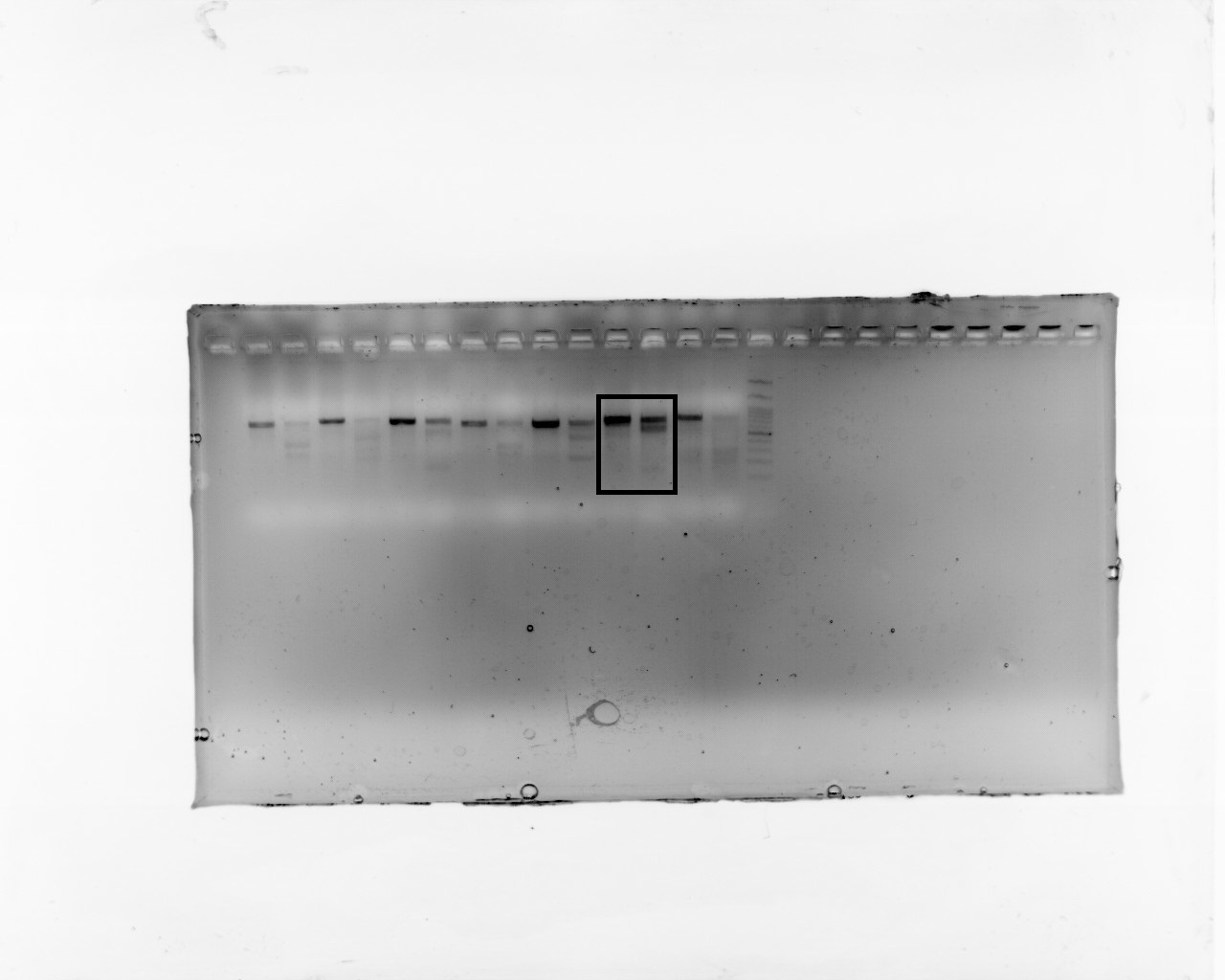

Supplement: Figure 2—figure supplement 1—source data 2. [file elife-93921-fig2-figsupp1-data2.zip › S2B-sgABL2-T7 assay.jpg]

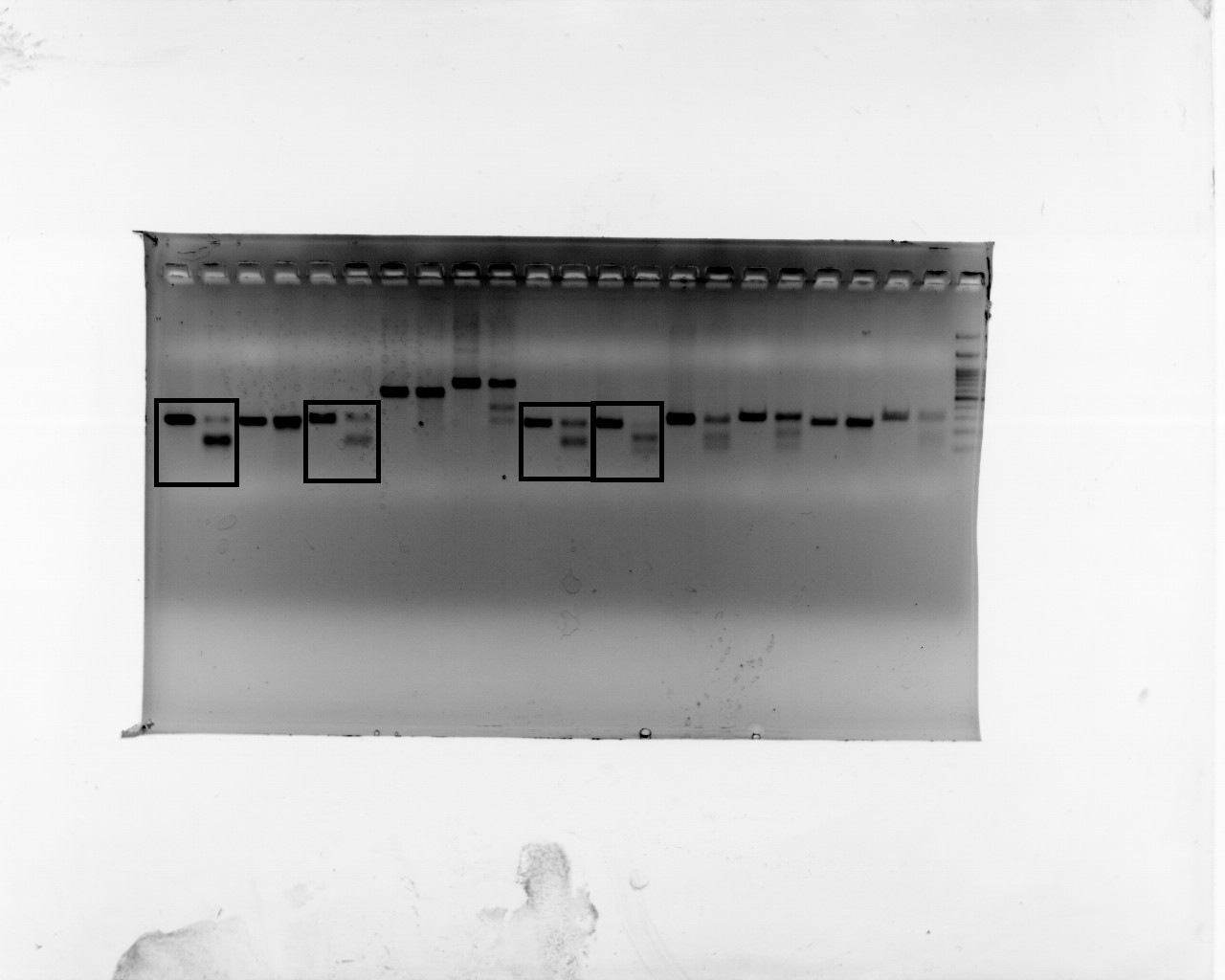

Supplement: Figure 2—figure supplement 1—source data 2. [file elife-93921-fig2-figsupp1-data2.zip › S2B-sgFGFR2 YES EGFR IGF1R T7 assay.tif]

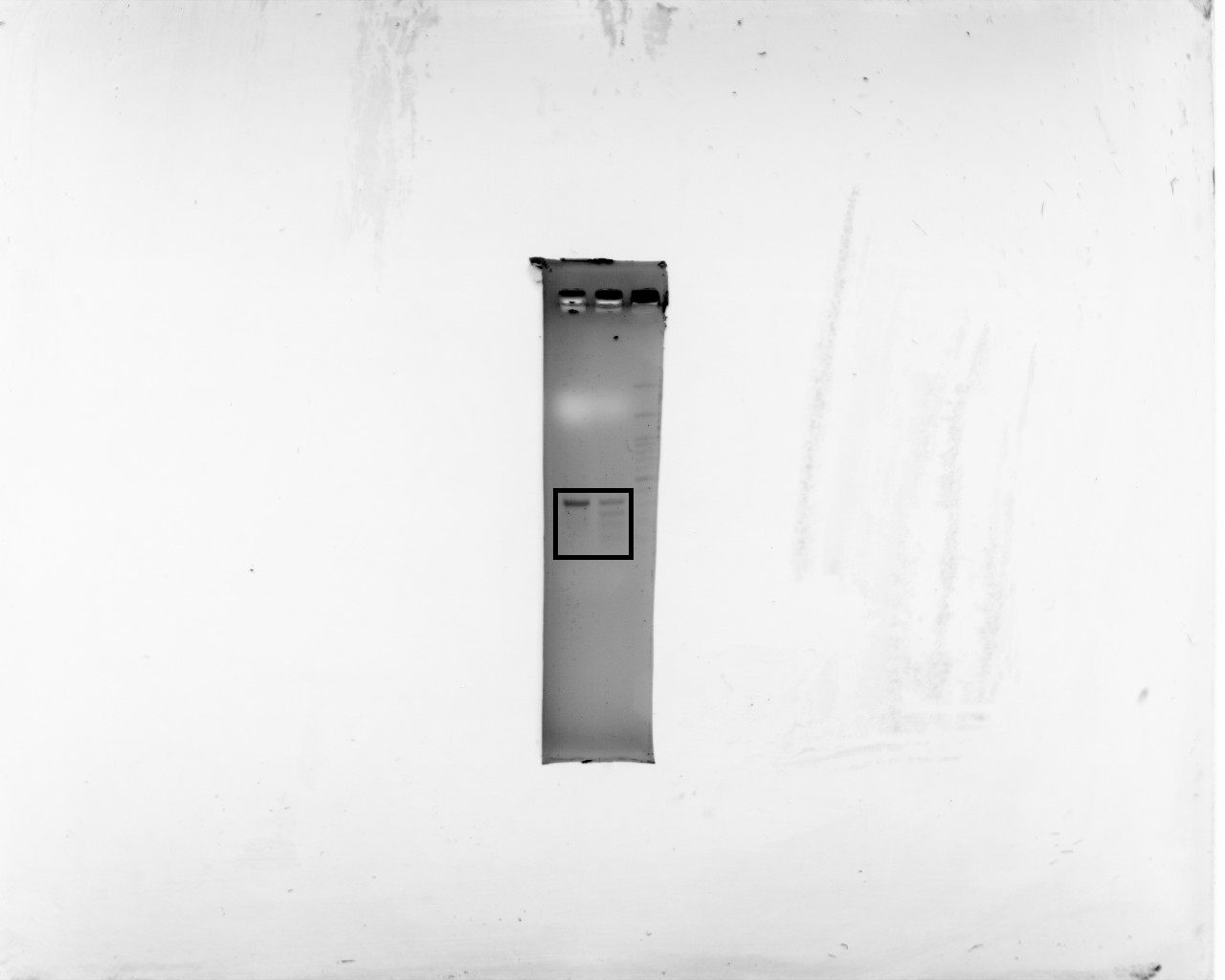

Supplement: Figure 2—figure supplement 1—source data 2. [file elife-93921-fig2-figsupp1-data2.zip › S2B-sgPTK6 T7 assay.tif]

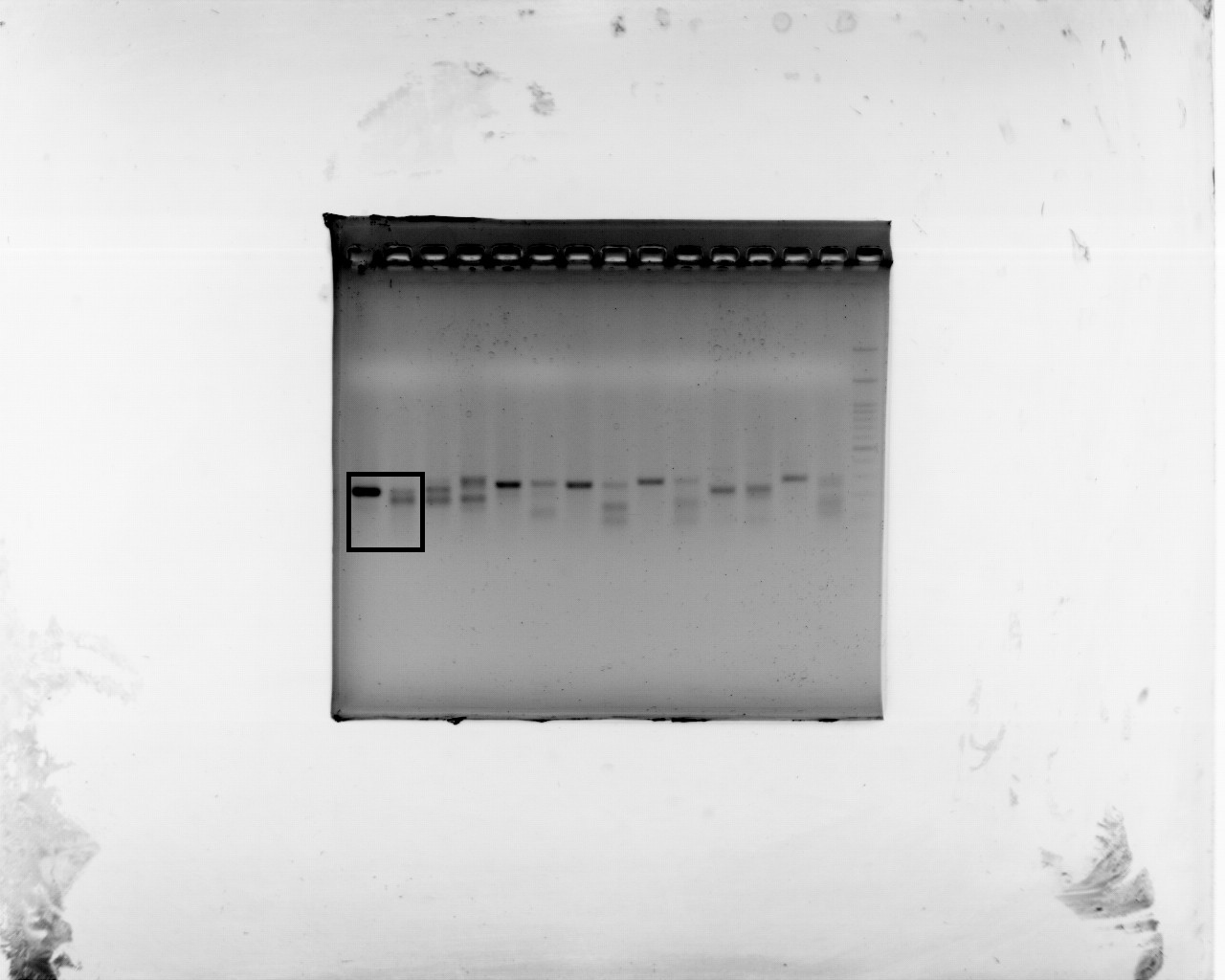

Supplement: Figure 2—figure supplement 1—source data 2. [file elife-93921-fig2-figsupp1-data2.zip › S2B-sgSRC-T7 assay.tif]

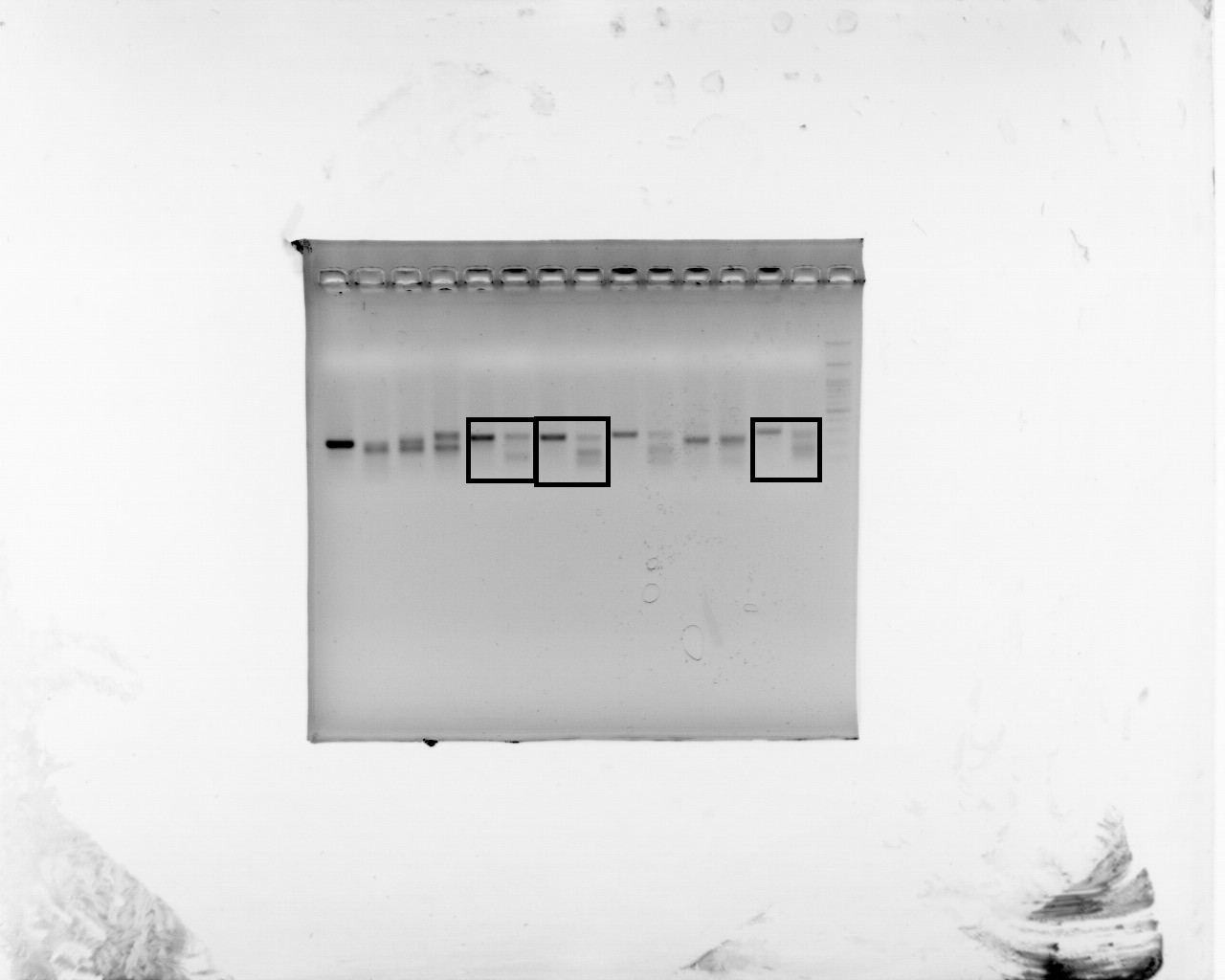

Supplement: Figure 2—figure supplement 1—source data 2. [file elife-93921-fig2-figsupp1-data2.zip › S2B-sgTEK FYN PDGFRA-T7 assay.tif]

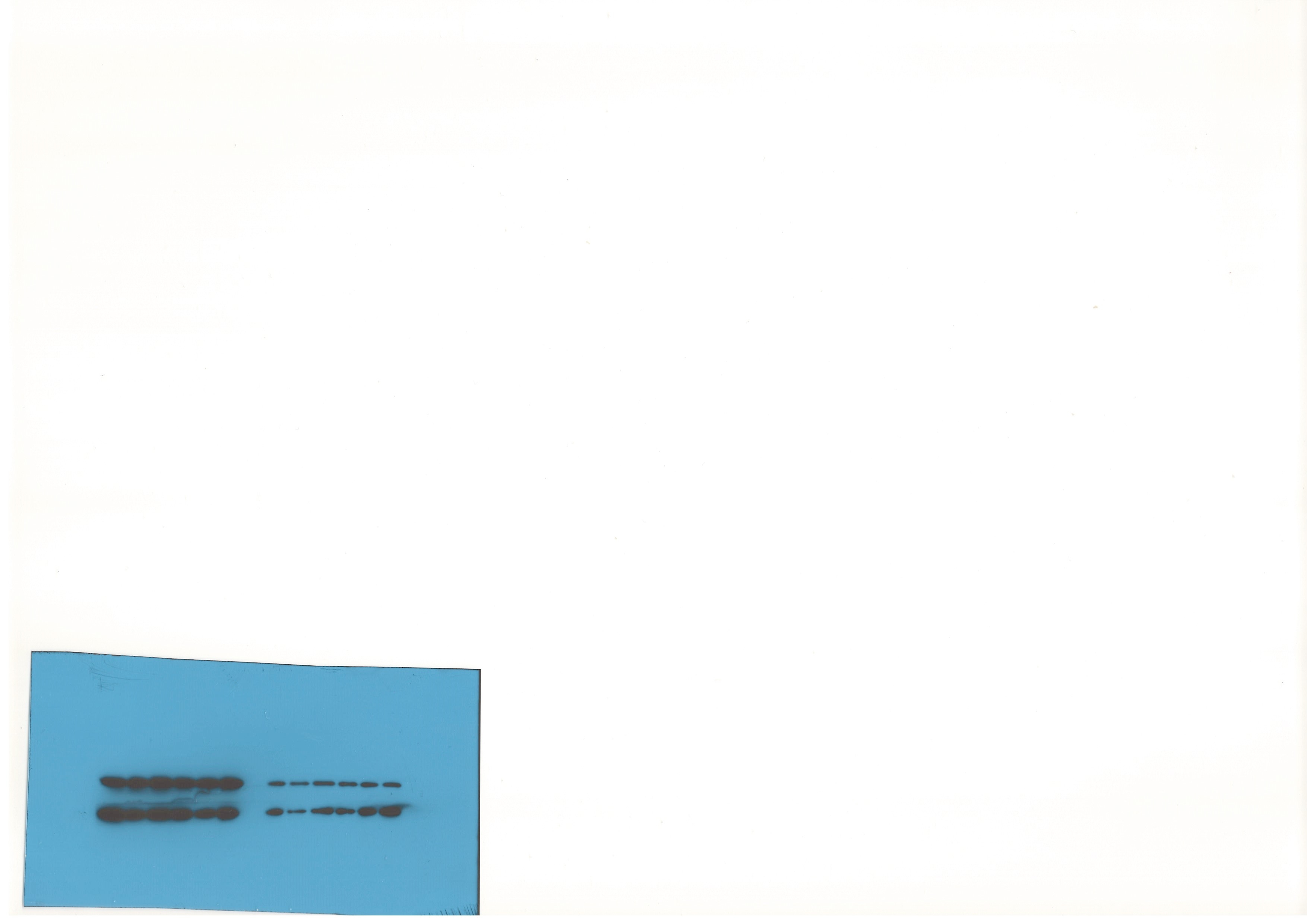

Supplement: Figure 2—figure supplement 3—source data 1. [file elife-93921-fig2-figsupp3-data1.zip › S3J-GAPDH p38.jpg]

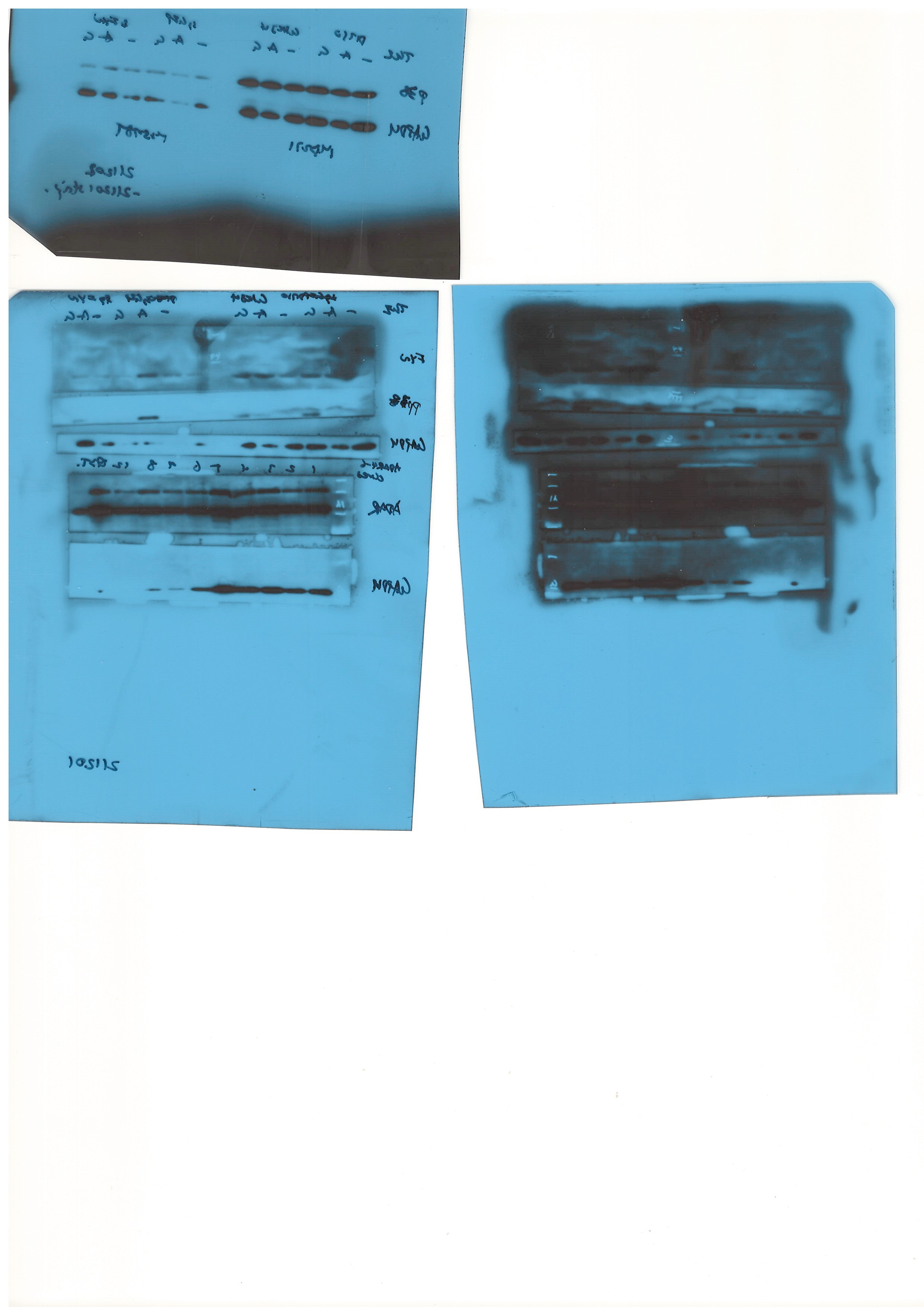

Supplement: Figure 2—figure supplement 3—source data 1. [file elife-93921-fig2-figsupp3-data1.zip › S3J-pp38 FYN.jpg]

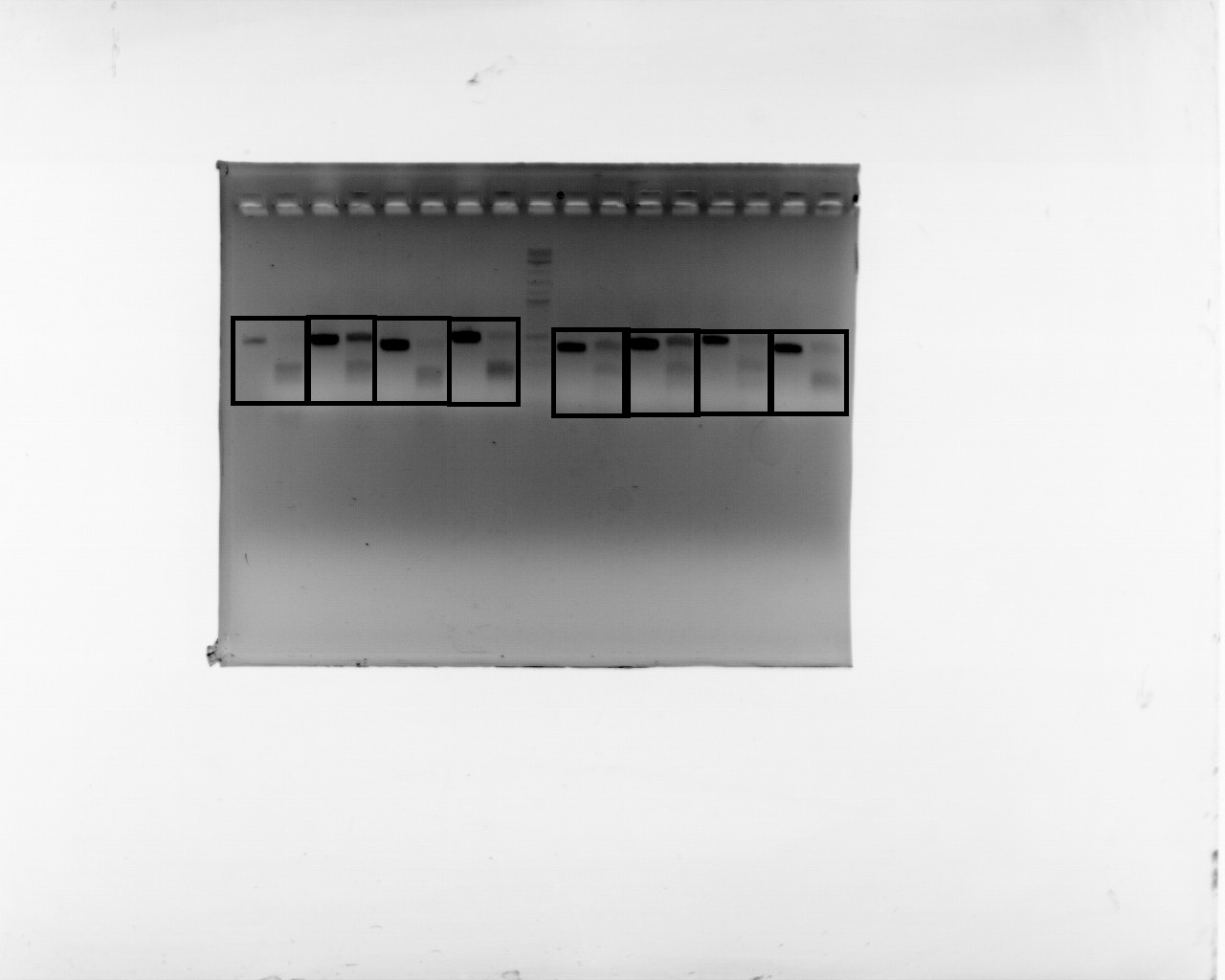

Supplement: Figure 2—figure supplement 3—source data 2. [file elife-93921-fig2-figsupp3-data2.zip › S5B.tif]

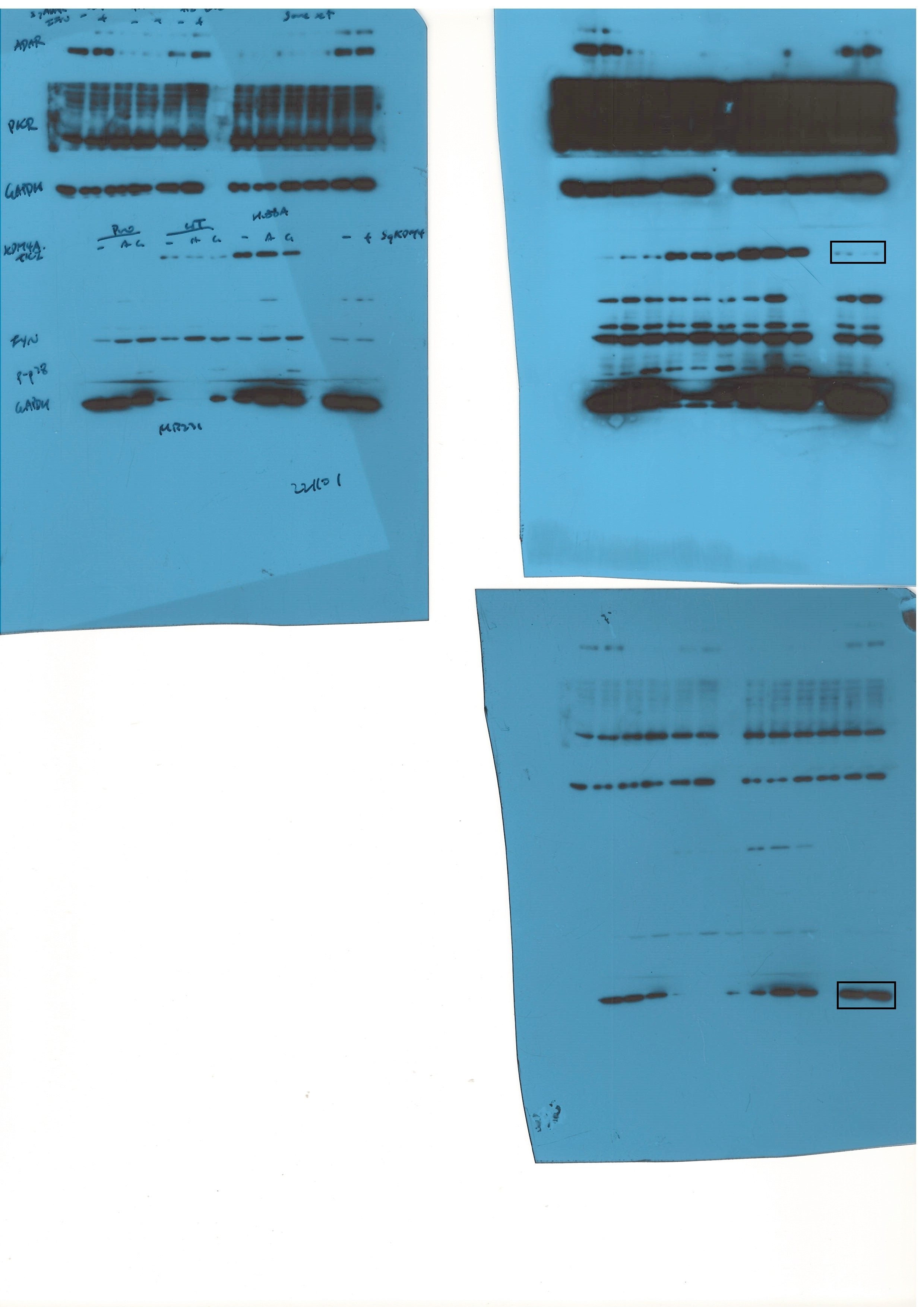

Supplement: Figure 2—figure supplement 3—source data 2. [file elife-93921-fig2-figsupp3-data2.zip › S5C.jpg]

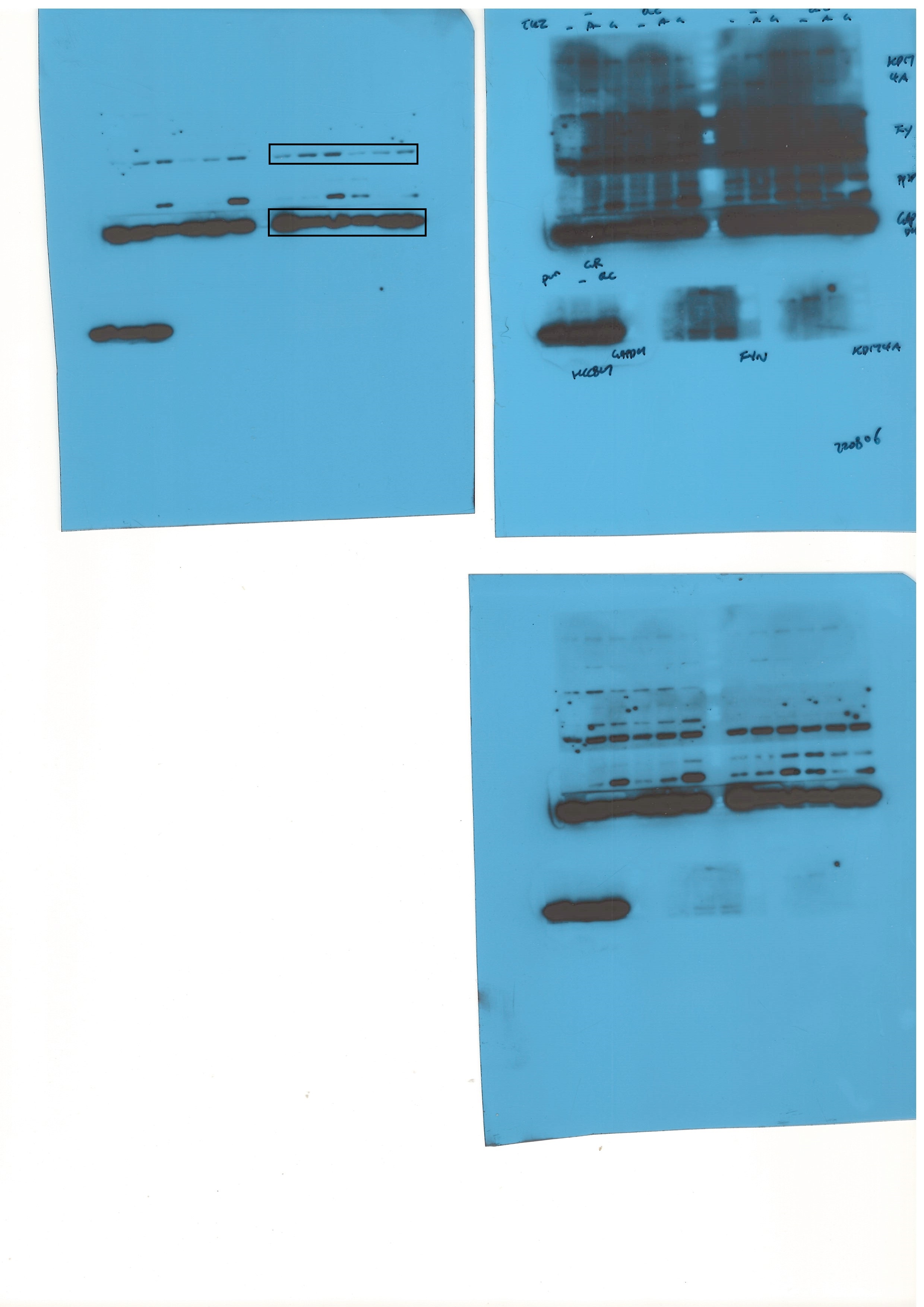

Supplement: Figure 2—figure supplement 3—source data 2. [file elife-93921-fig2-figsupp3-data2.zip › S5F-left.jpg]

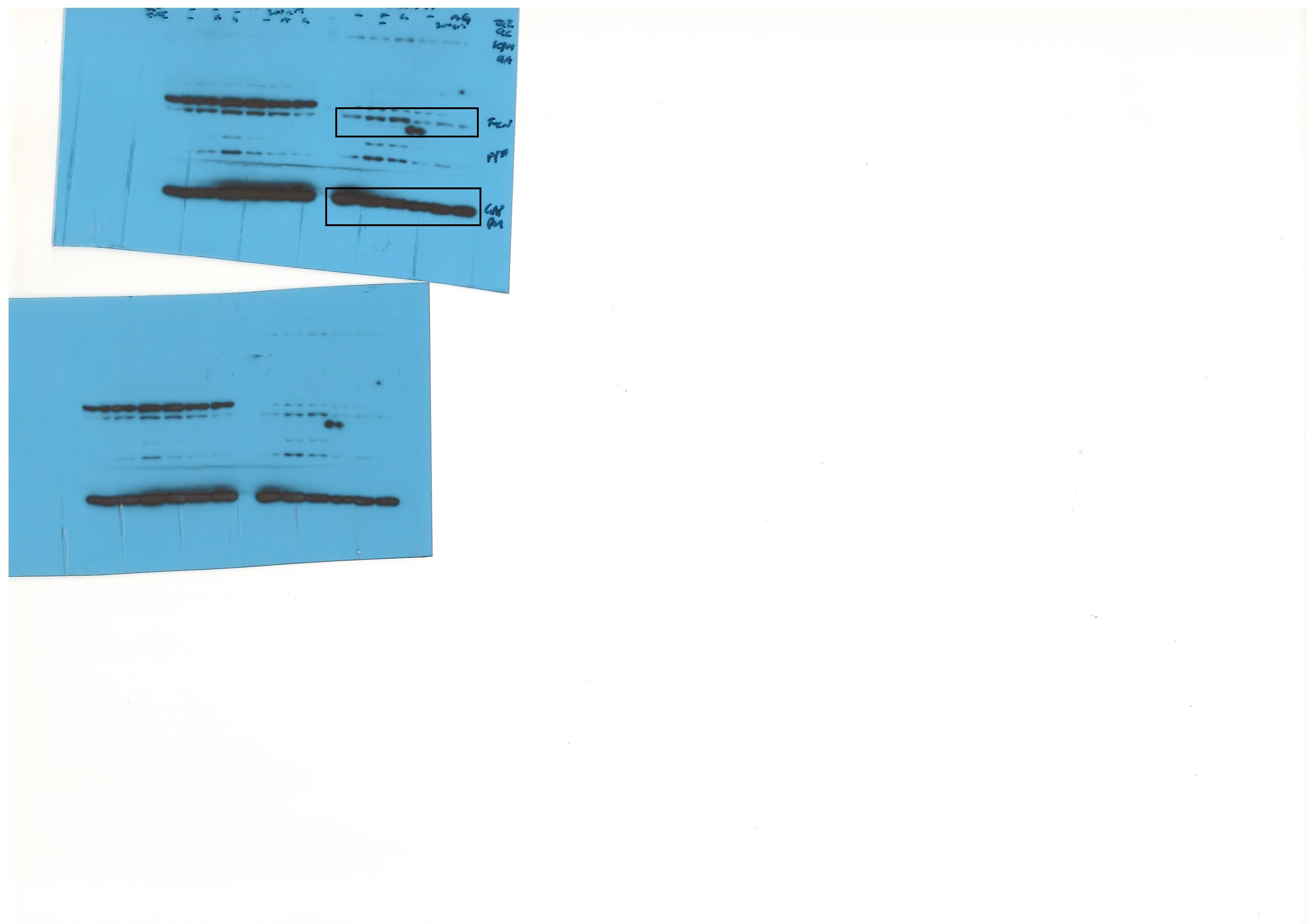

Supplement: Figure 2—figure supplement 3—source data 2. [file elife-93921-fig2-figsupp3-data2.zip › S5F-middle.jpg]

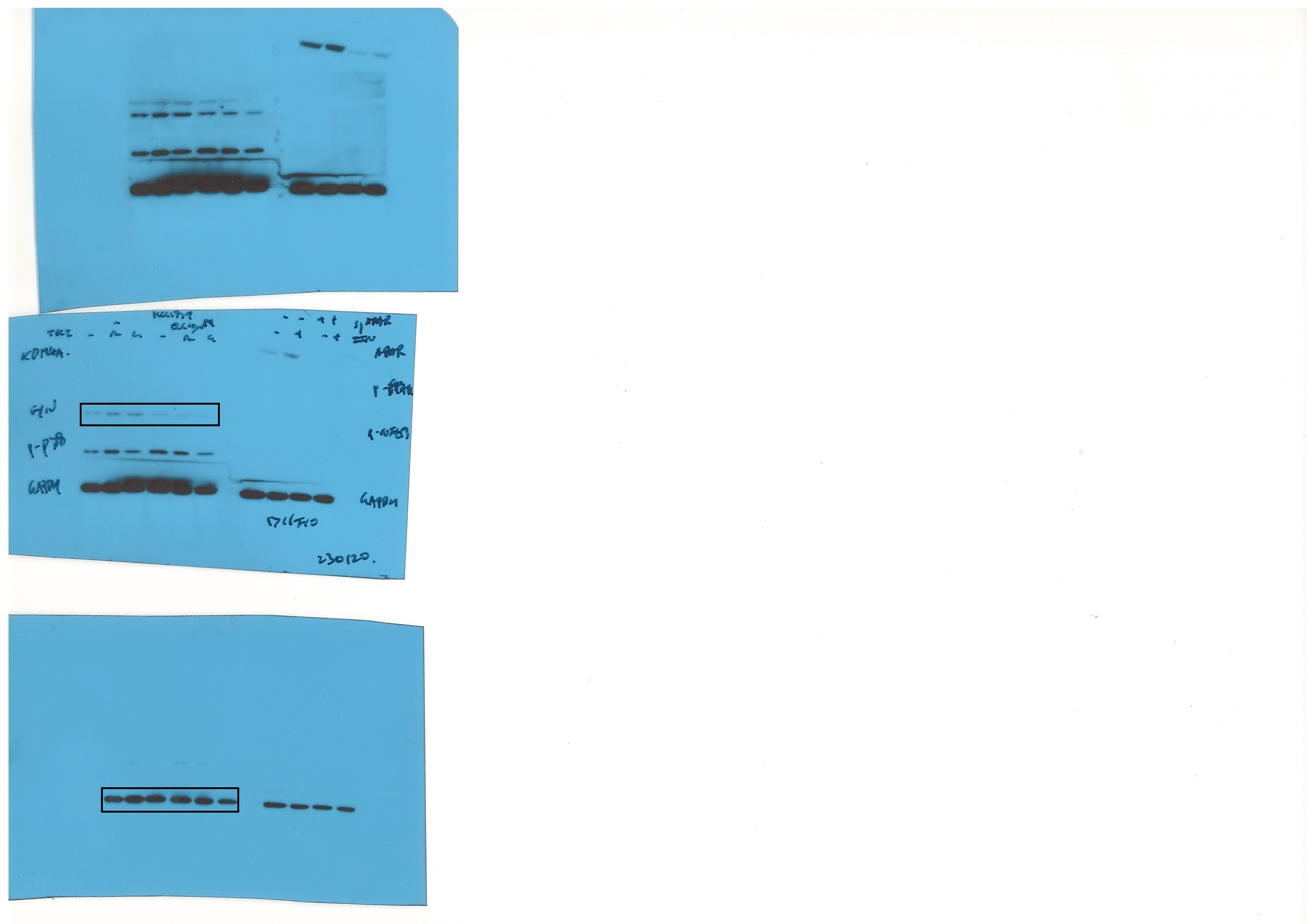

Supplement: Figure 2—figure supplement 3—source data 2. [file elife-93921-fig2-figsupp3-data2.zip › S5F-right.jpg]

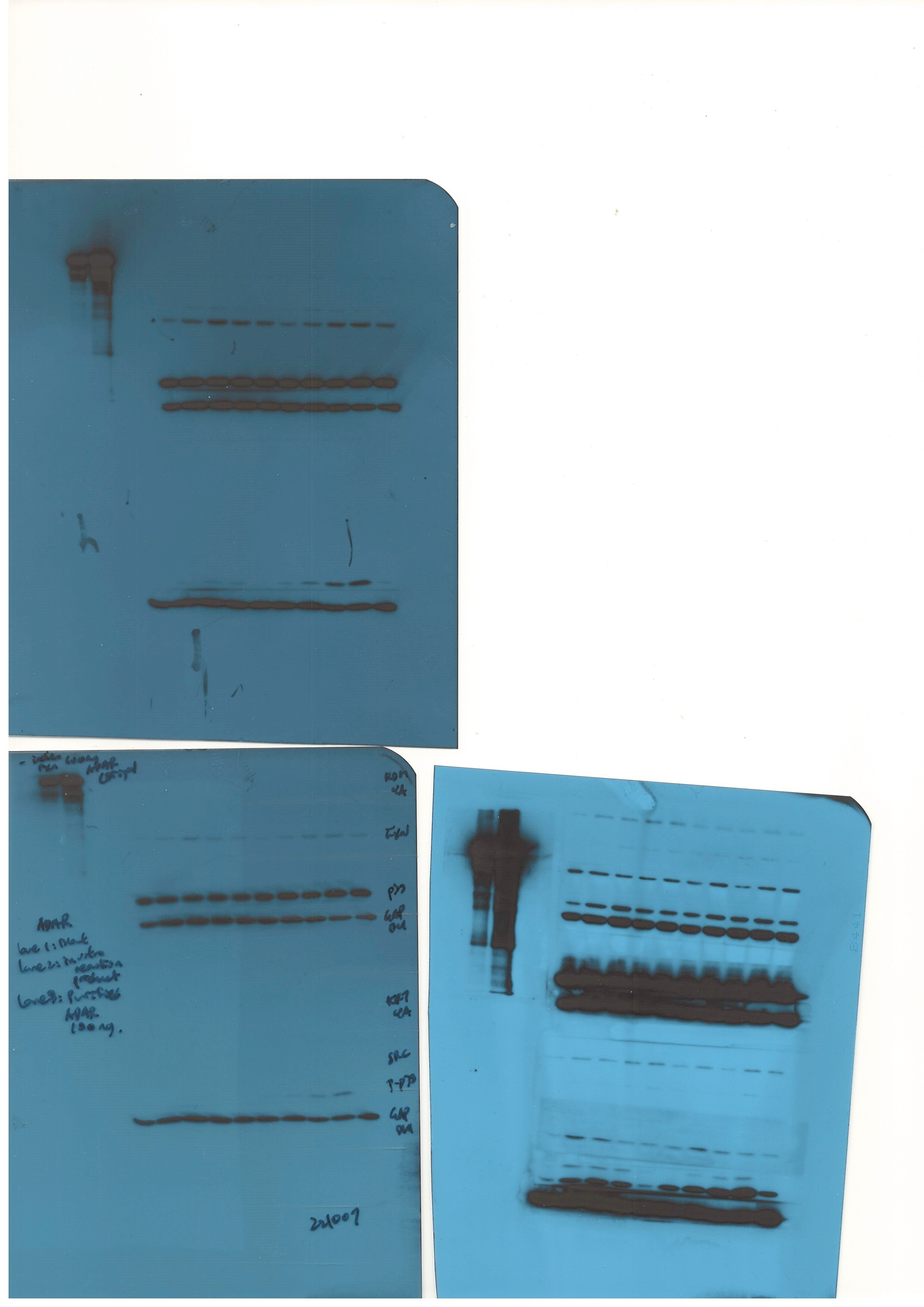

Supplement: Figure 3—source data 1. [file elife-93921-fig3-data1.zip › 3A.jpg]

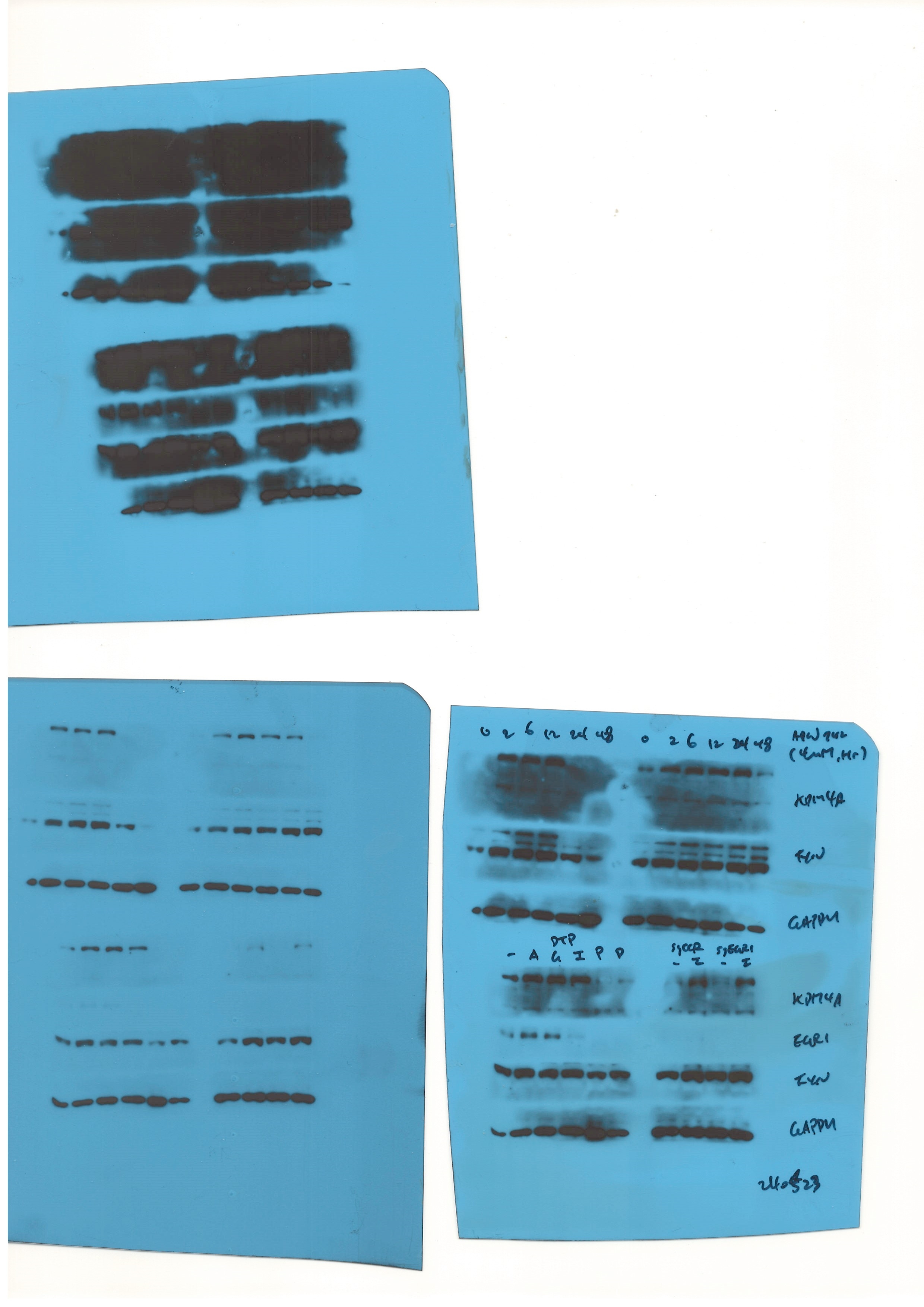

Supplement: Figure 3—source data 1. [file elife-93921-fig3-data1.zip › 3E.jpg]

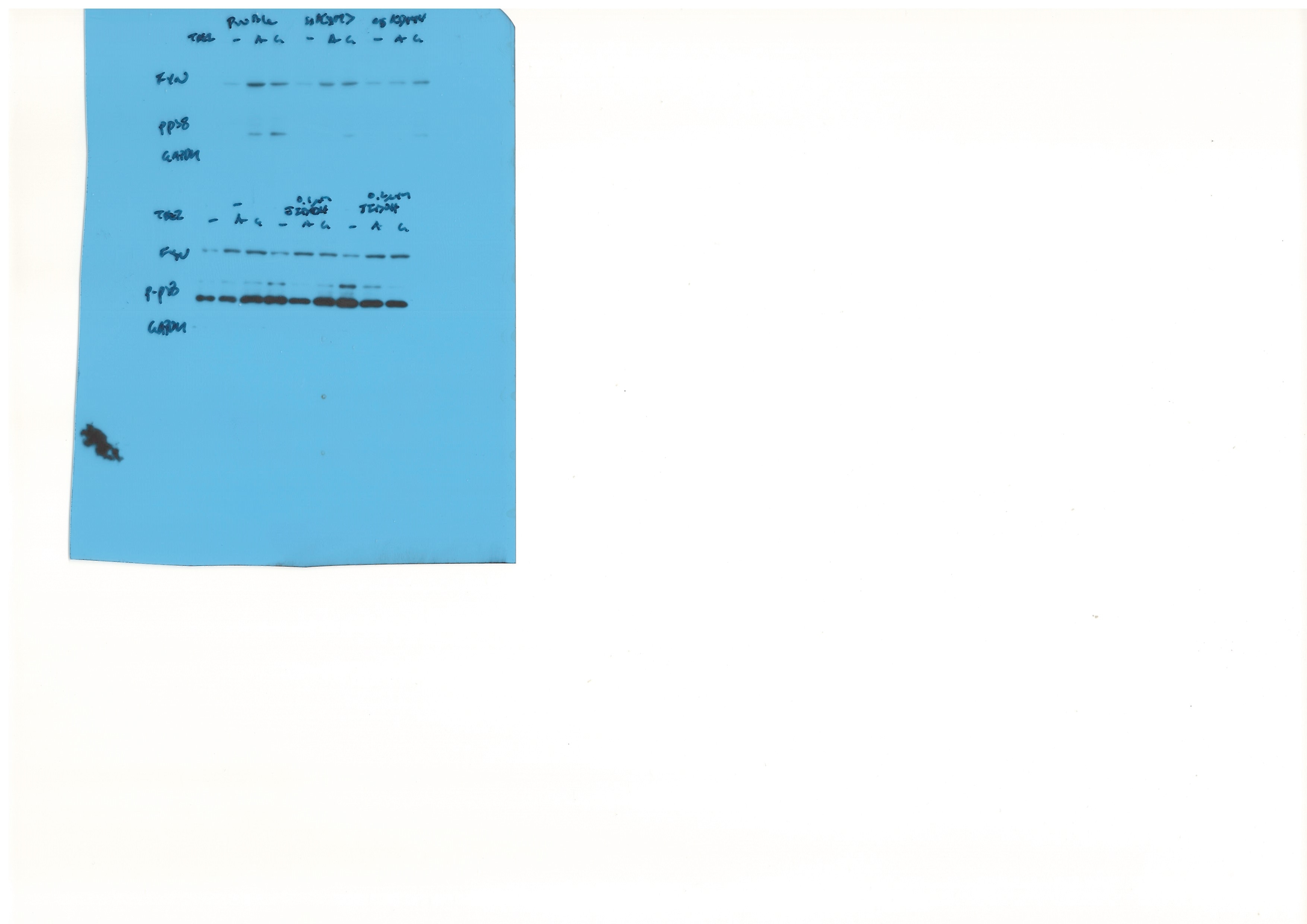

Supplement: Figure 3—source data 1. [file elife-93921-fig3-data1.zip › 3H-p38 GAPDH.jpg]

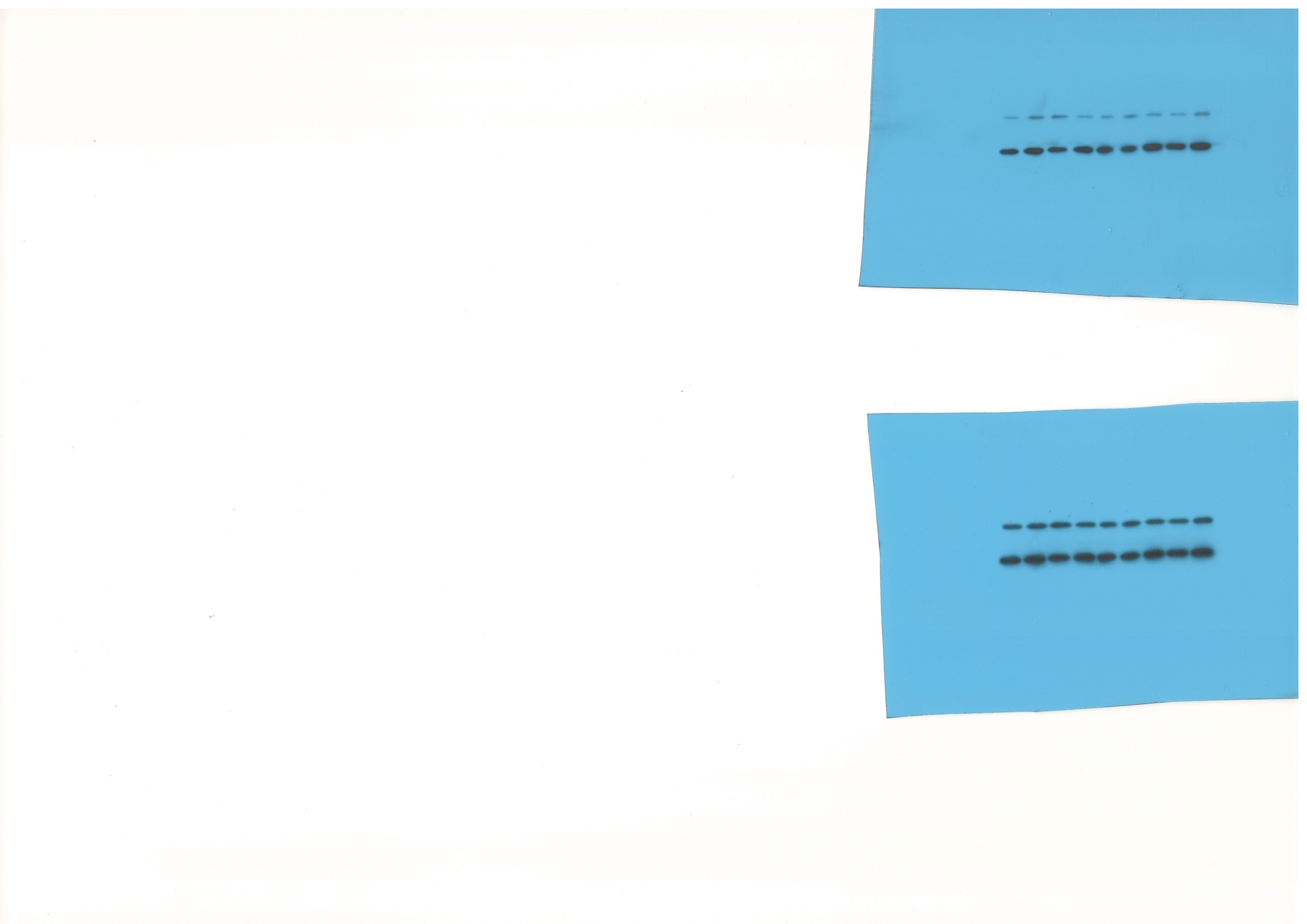

Supplement: Figure 3—source data 1. [file elife-93921-fig3-data1.zip › 3H-pp38 FYN.jpg]

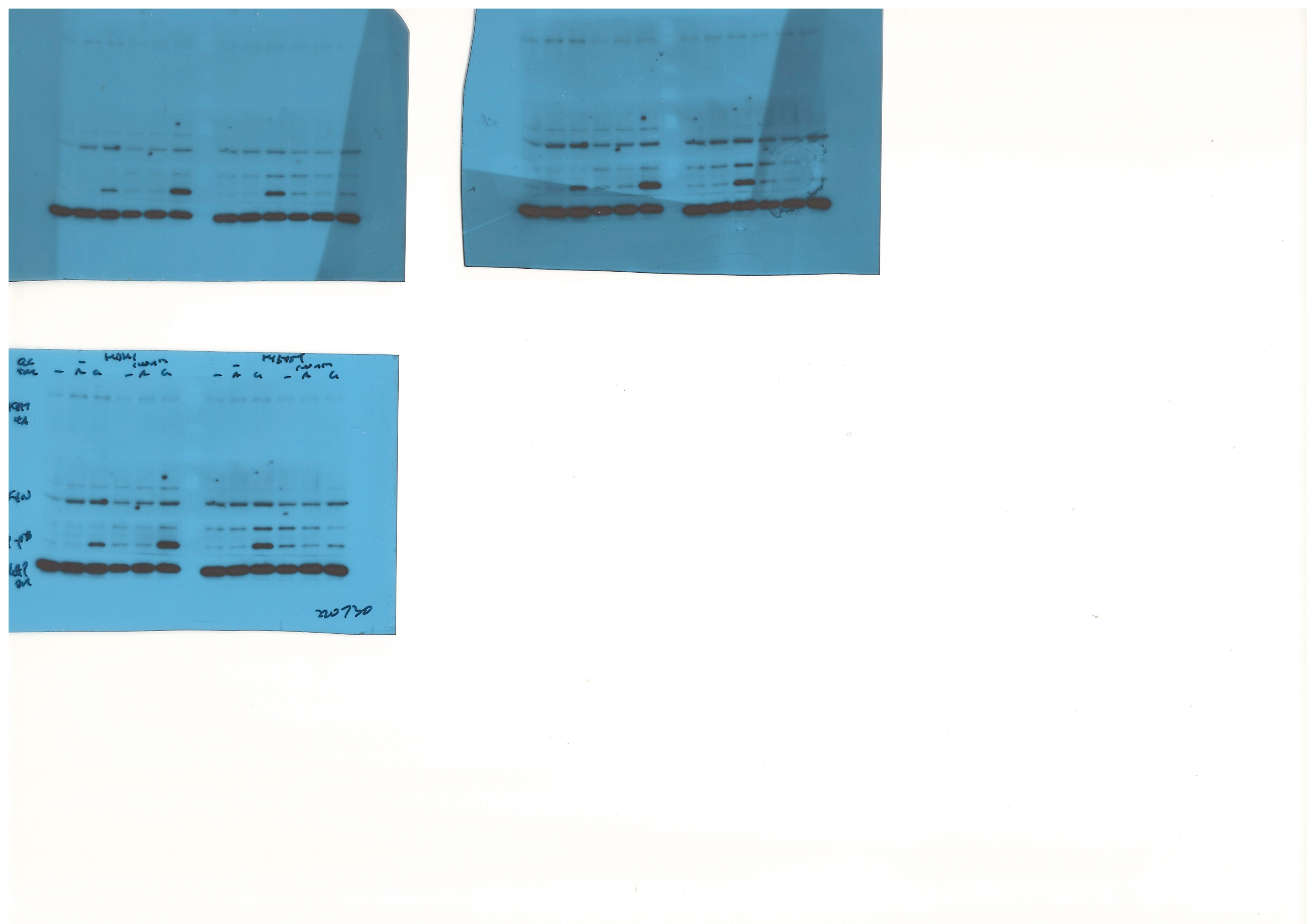

Supplement: Figure 3—source data 1. [file elife-93921-fig3-data1.zip › 3K.jpg]

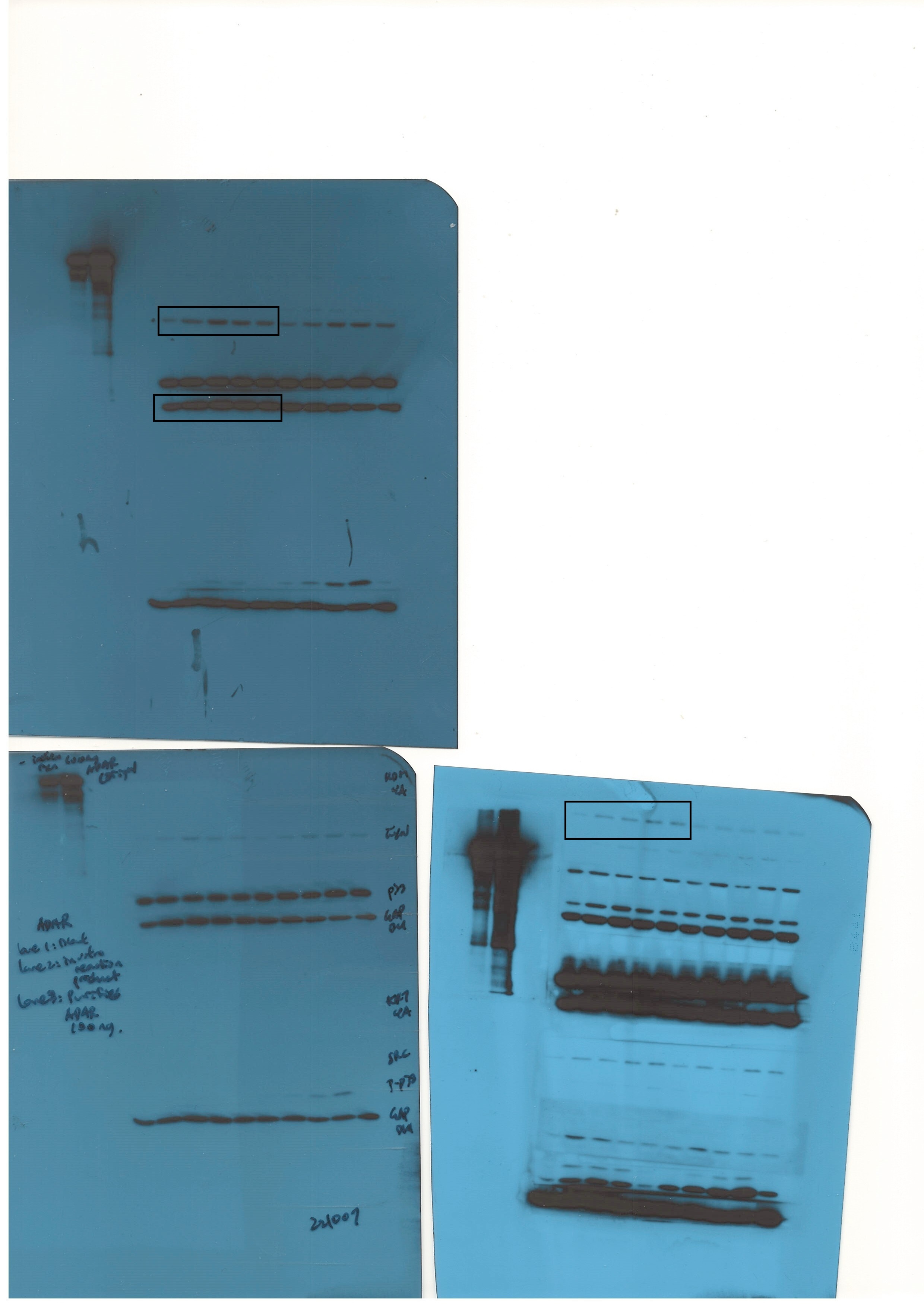

Supplement: Figure 3—source data 2. [file elife-93921-fig3-data2.zip › 3A.jpg]

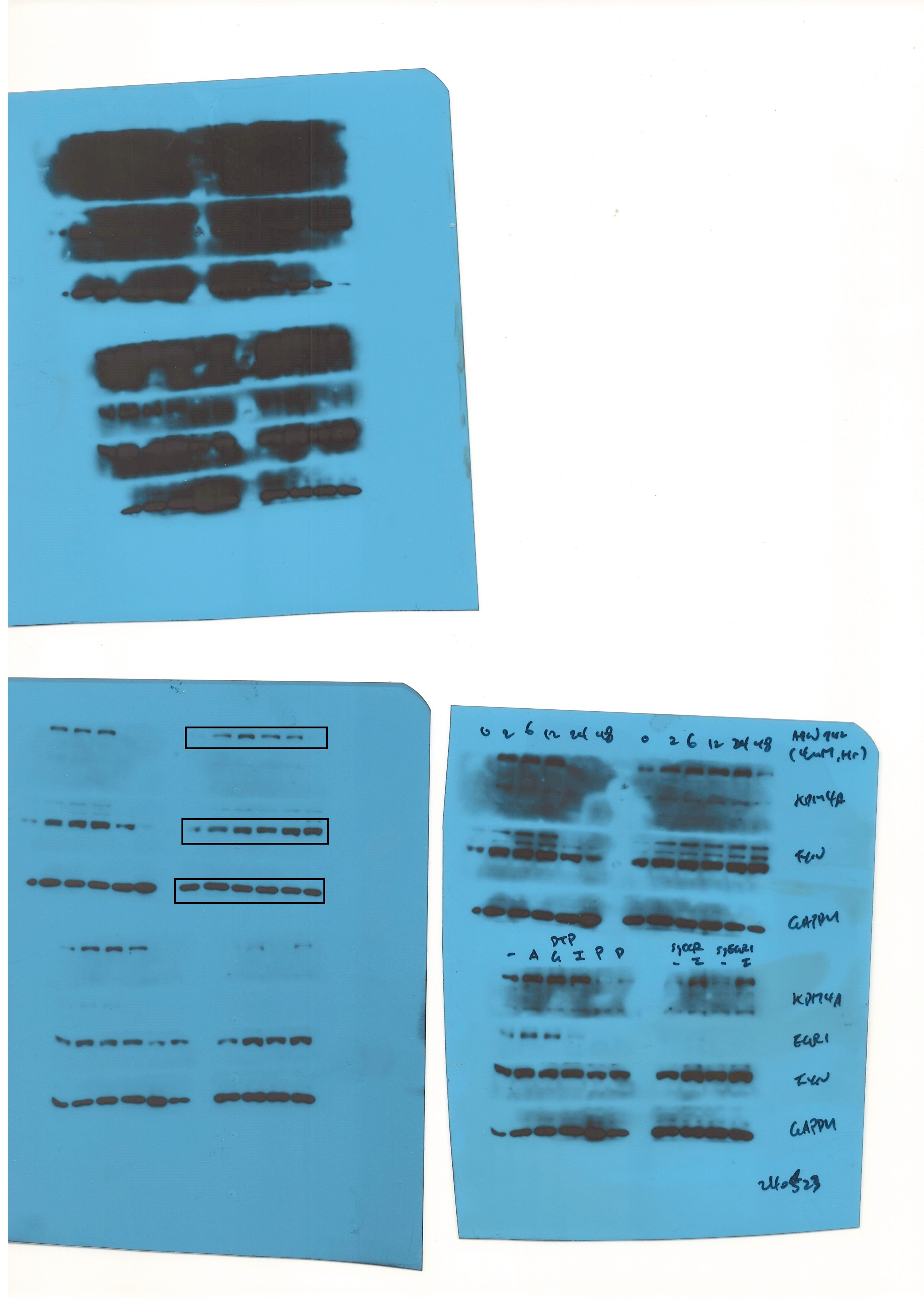

Supplement: Figure 3—source data 2. [file elife-93921-fig3-data2.zip › 3E.jpg]

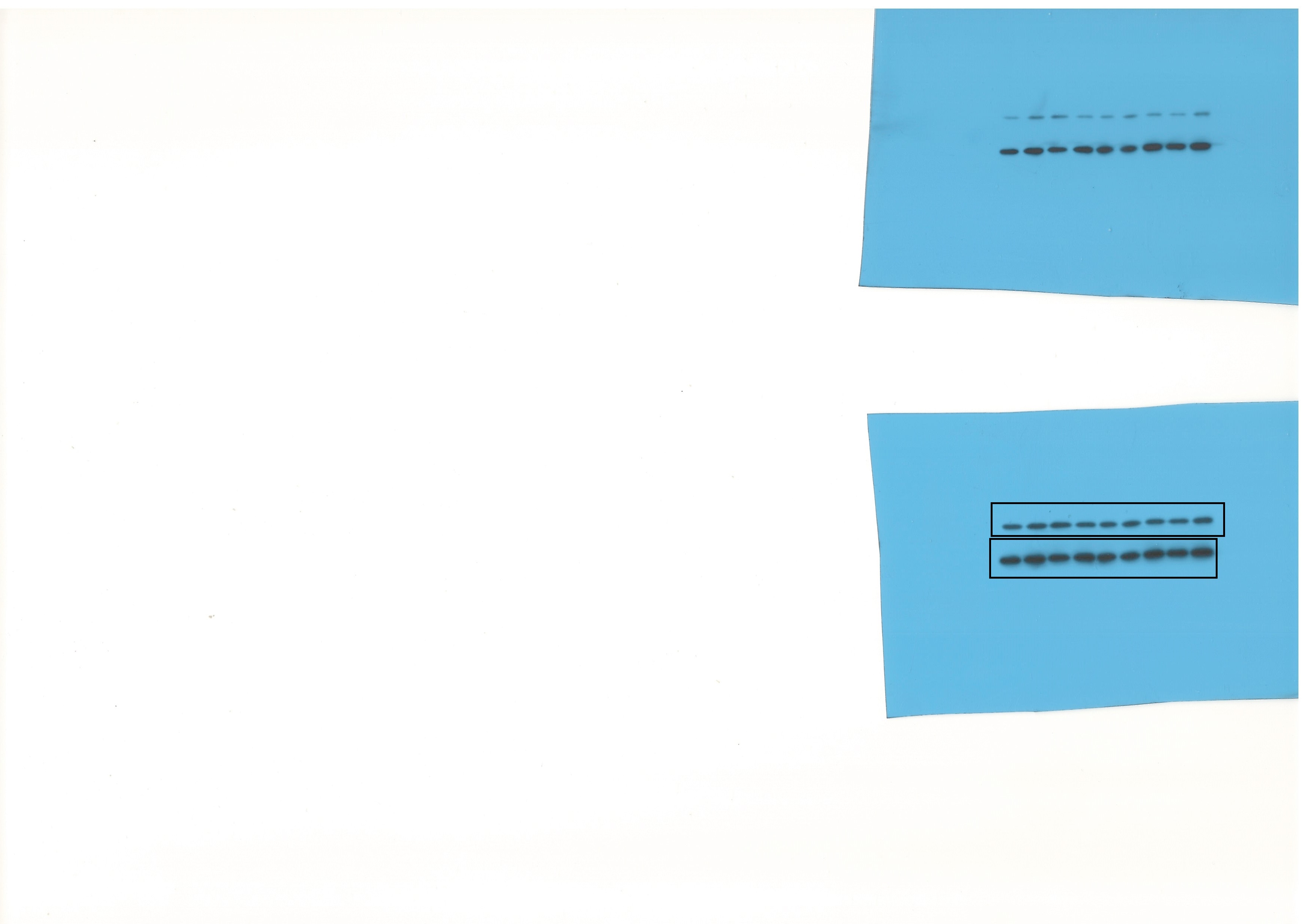

Supplement: Figure 3—source data 2. [file elife-93921-fig3-data2.zip › 3H-p38 GAPDH.jpg]

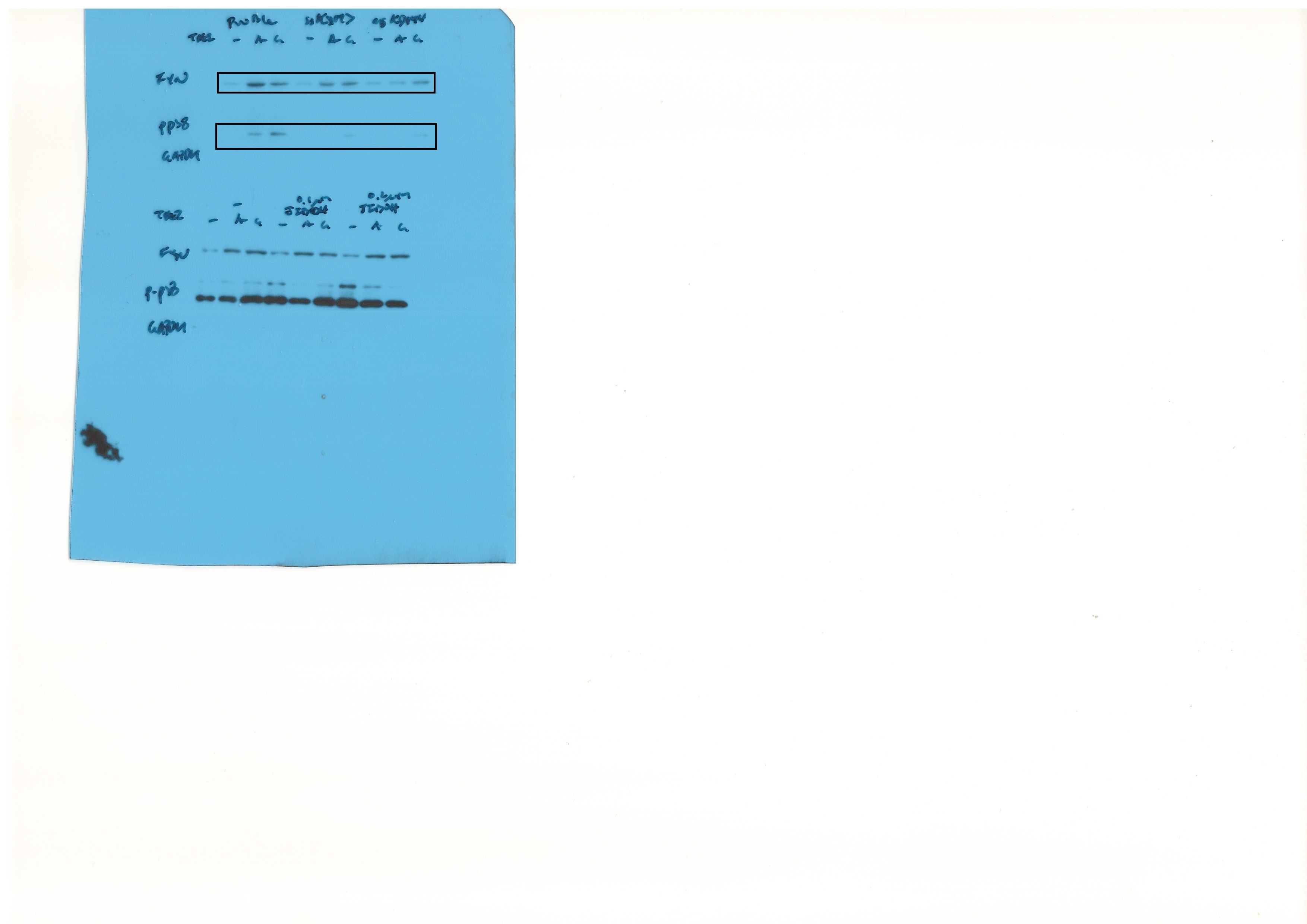

Supplement: Figure 3—source data 2. [file elife-93921-fig3-data2.zip › 3H-pp38 FYN.jpg]

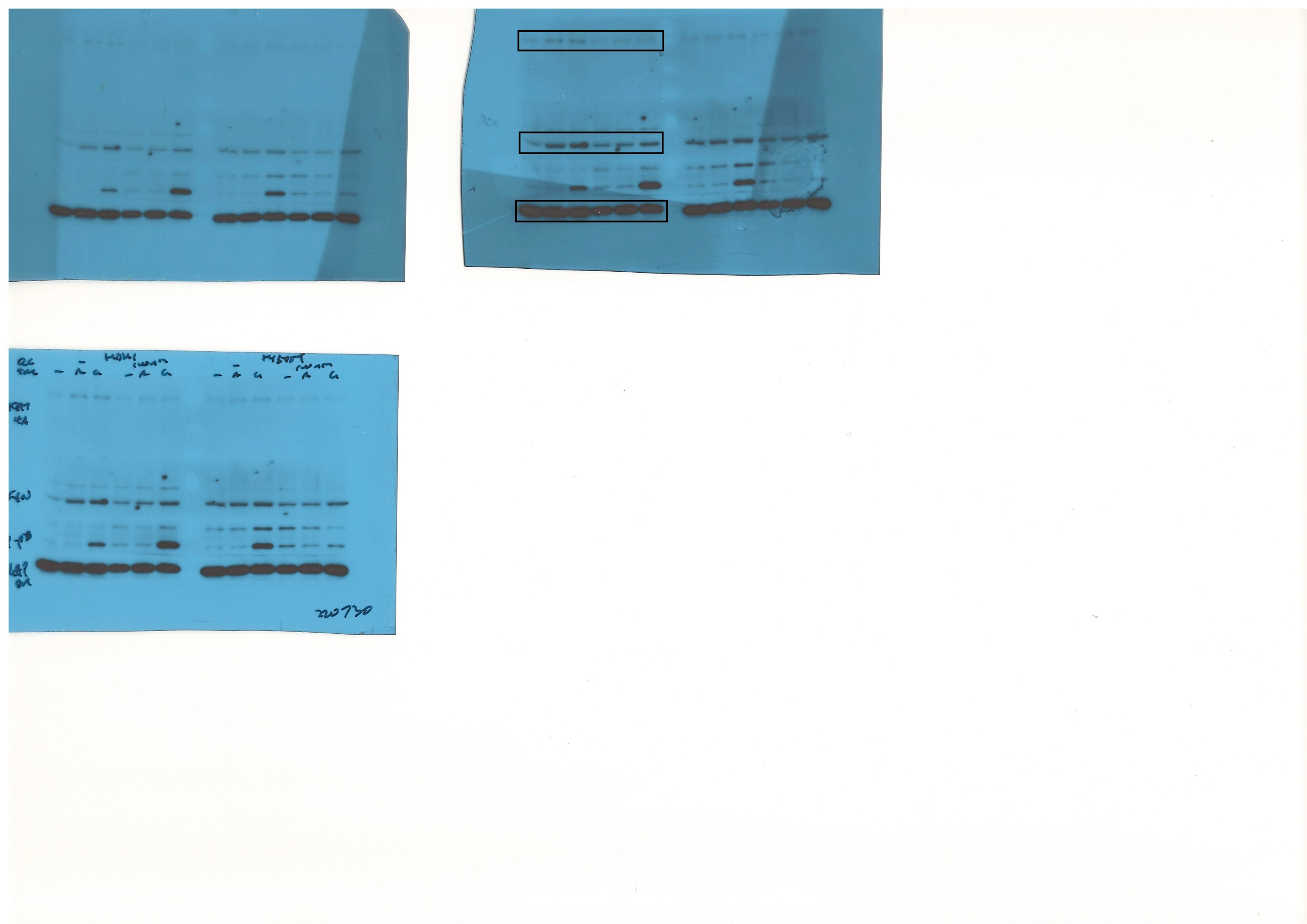

Supplement: Figure 3—source data 2. [file elife-93921-fig3-data2.zip › 3K.jpg]

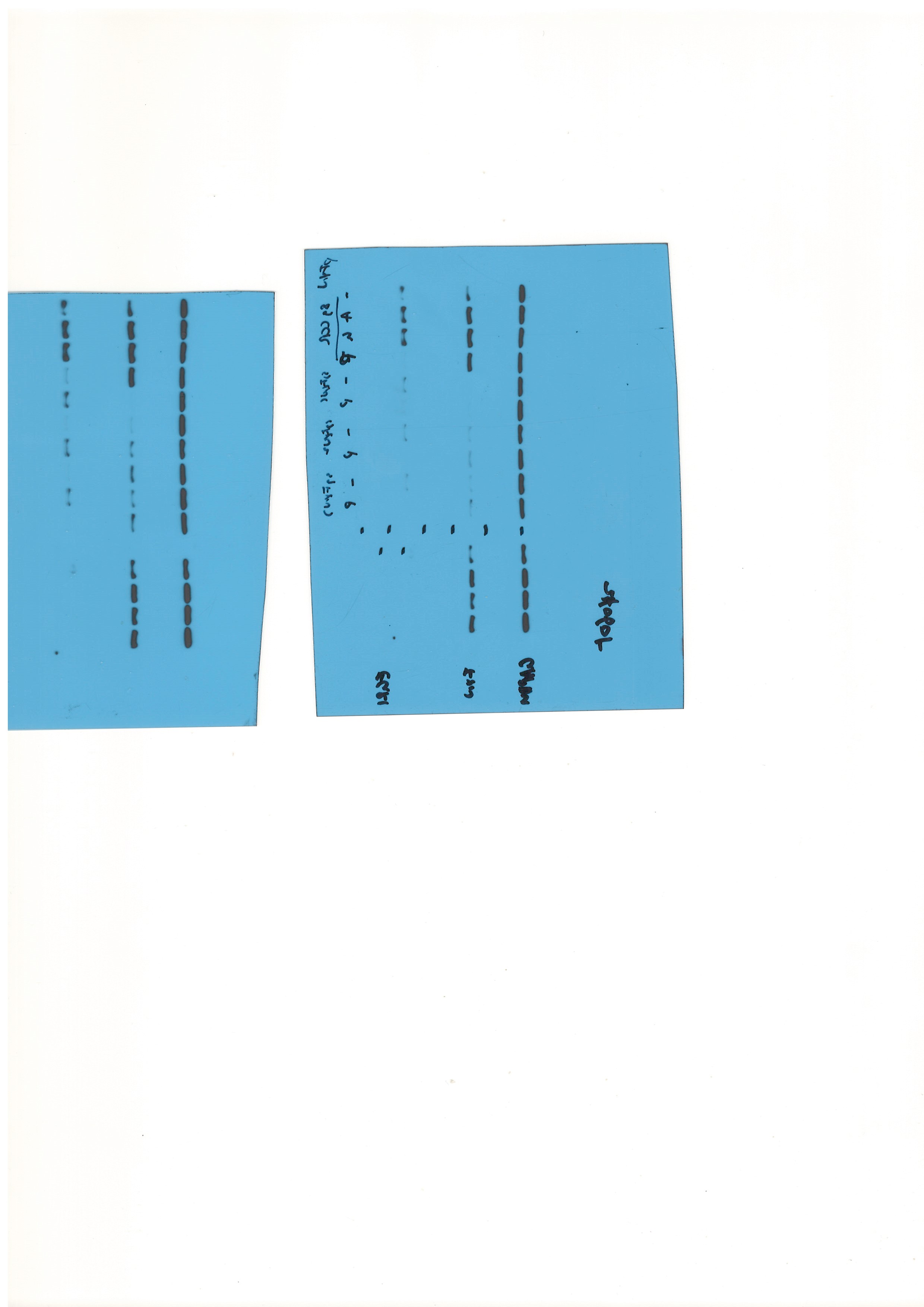

Supplement: Figure 3—figure supplement 1—source data 1. [file elife-93921-fig3-figsupp1-data1.zip › S4A.jpg]

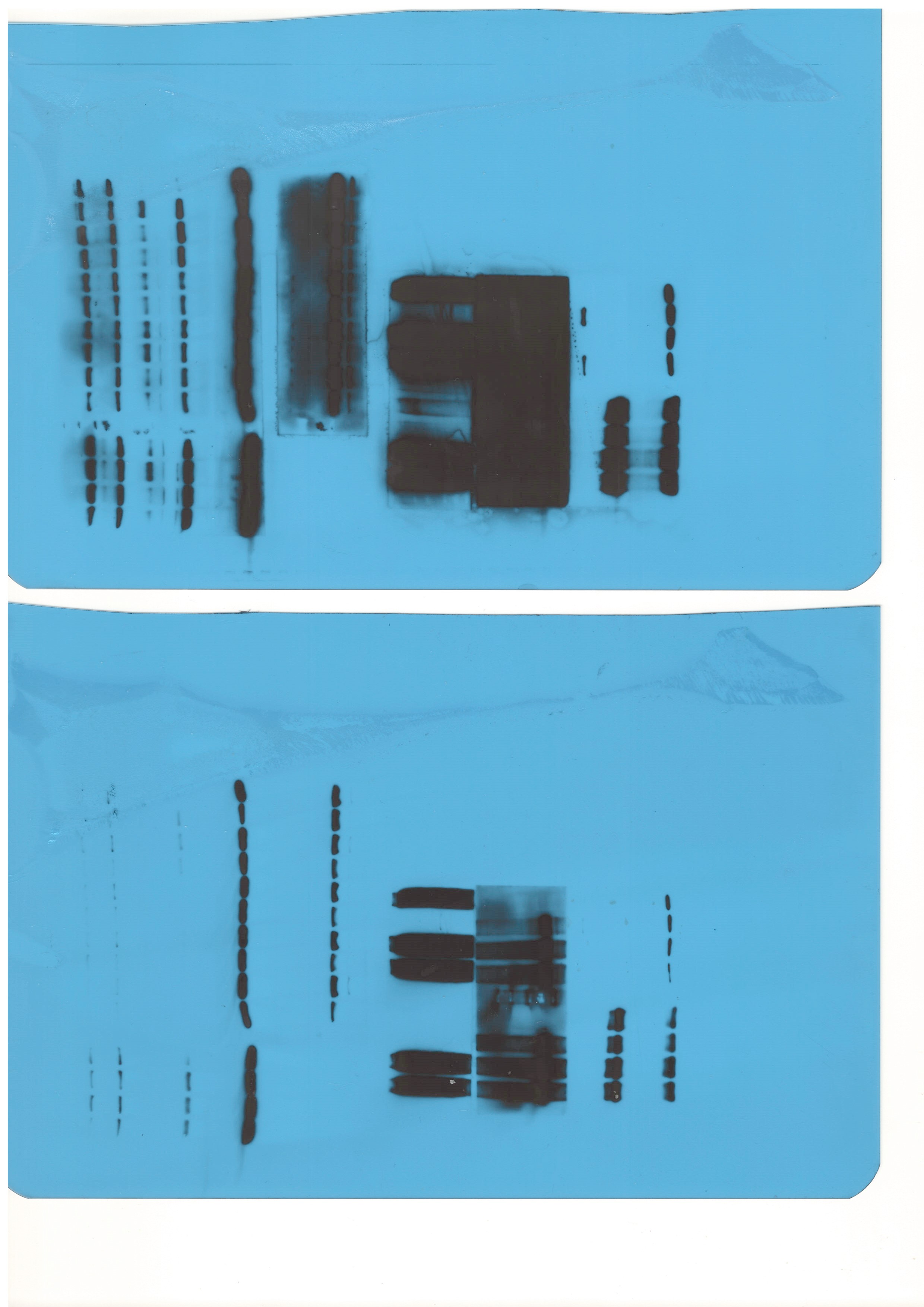

Supplement: Figure 3—figure supplement 1—source data 1. [file elife-93921-fig3-figsupp1-data1.zip › S4B.jpg]

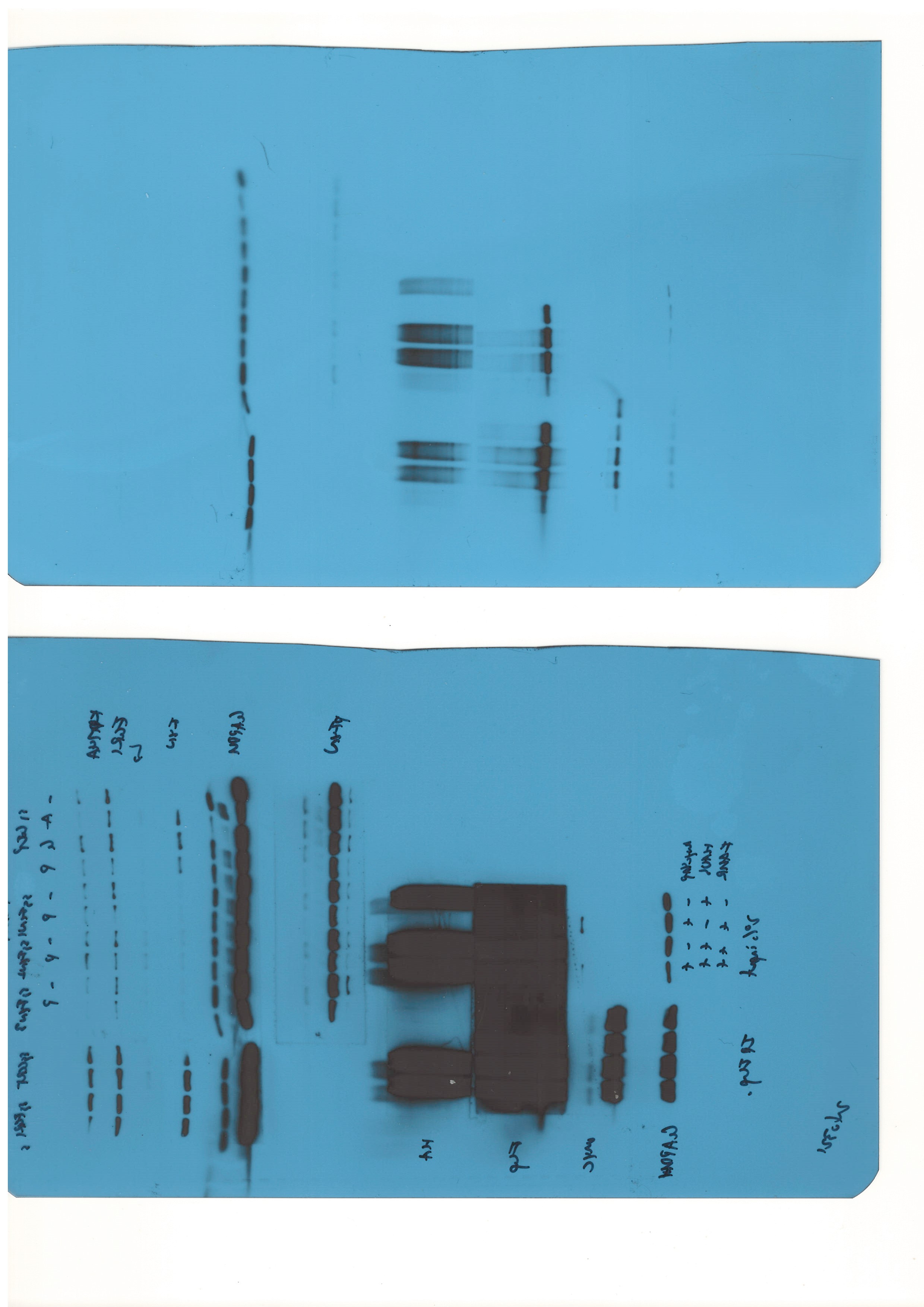

Supplement: Figure 3—figure supplement 1—source data 1. [file elife-93921-fig3-figsupp1-data1.zip › S4B-GAPDH.jpg]

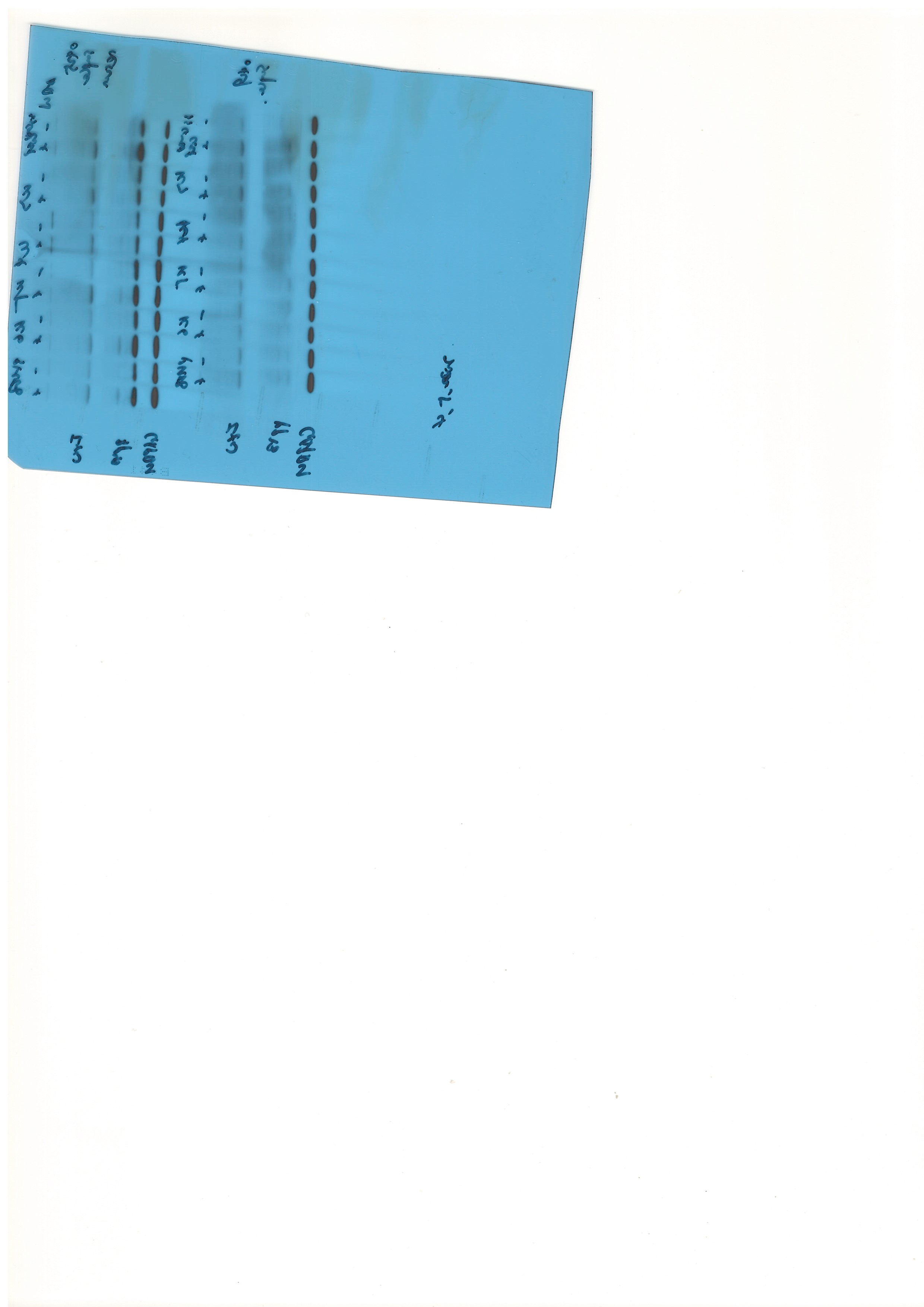

Supplement: Figure 3—figure supplement 1—source data 1. [file elife-93921-fig3-figsupp1-data1.zip › S4F.jpg]

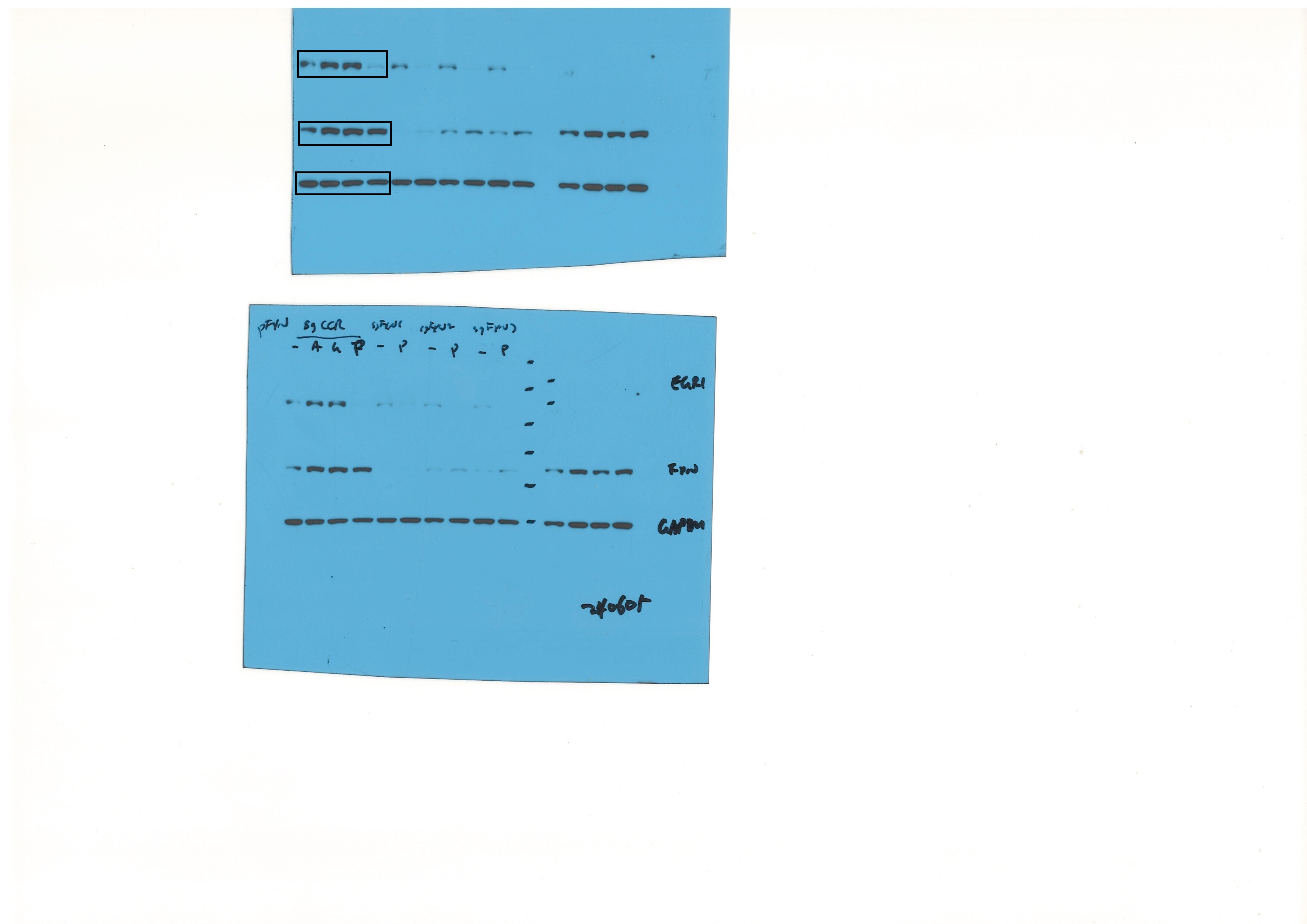

Supplement: Figure 3—figure supplement 1—source data 2. [file elife-93921-fig3-figsupp1-data2.zip › S4A.jpg]

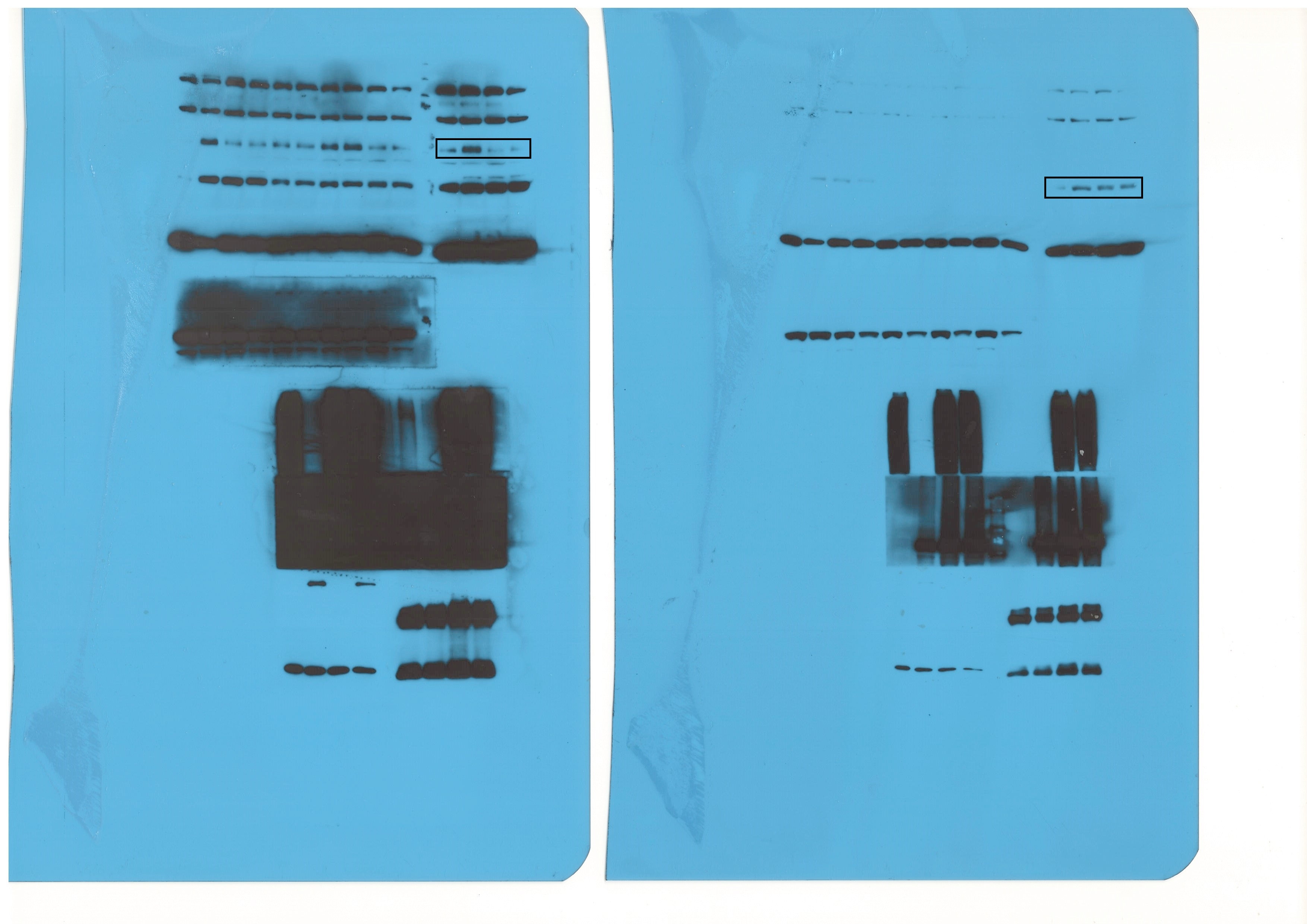

Supplement: Figure 3—figure supplement 1—source data 2. [file elife-93921-fig3-figsupp1-data2.zip › S4B.jpg]

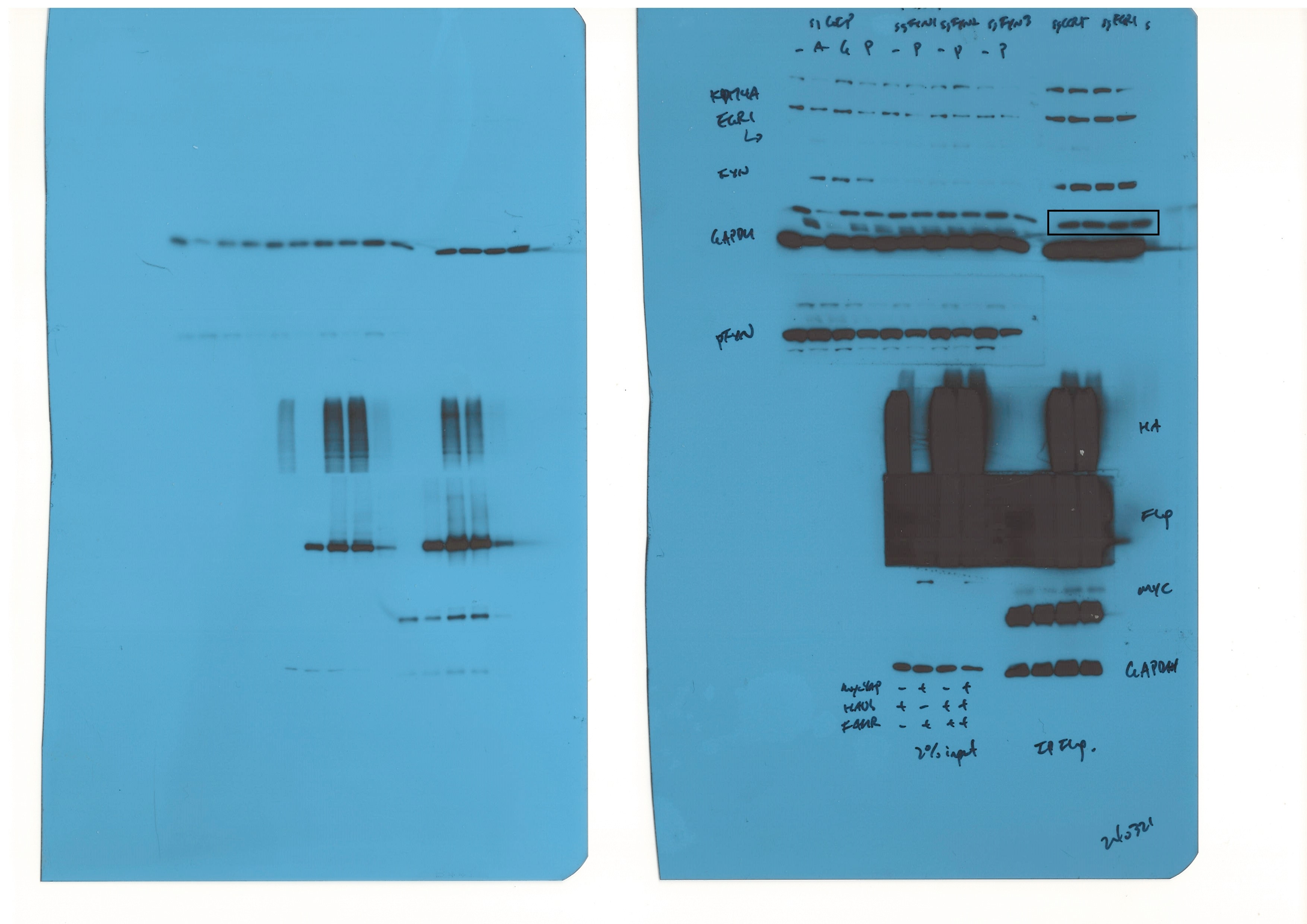

Supplement: Figure 3—figure supplement 1—source data 2. [file elife-93921-fig3-figsupp1-data2.zip › S4B-GAPDH.jpg]

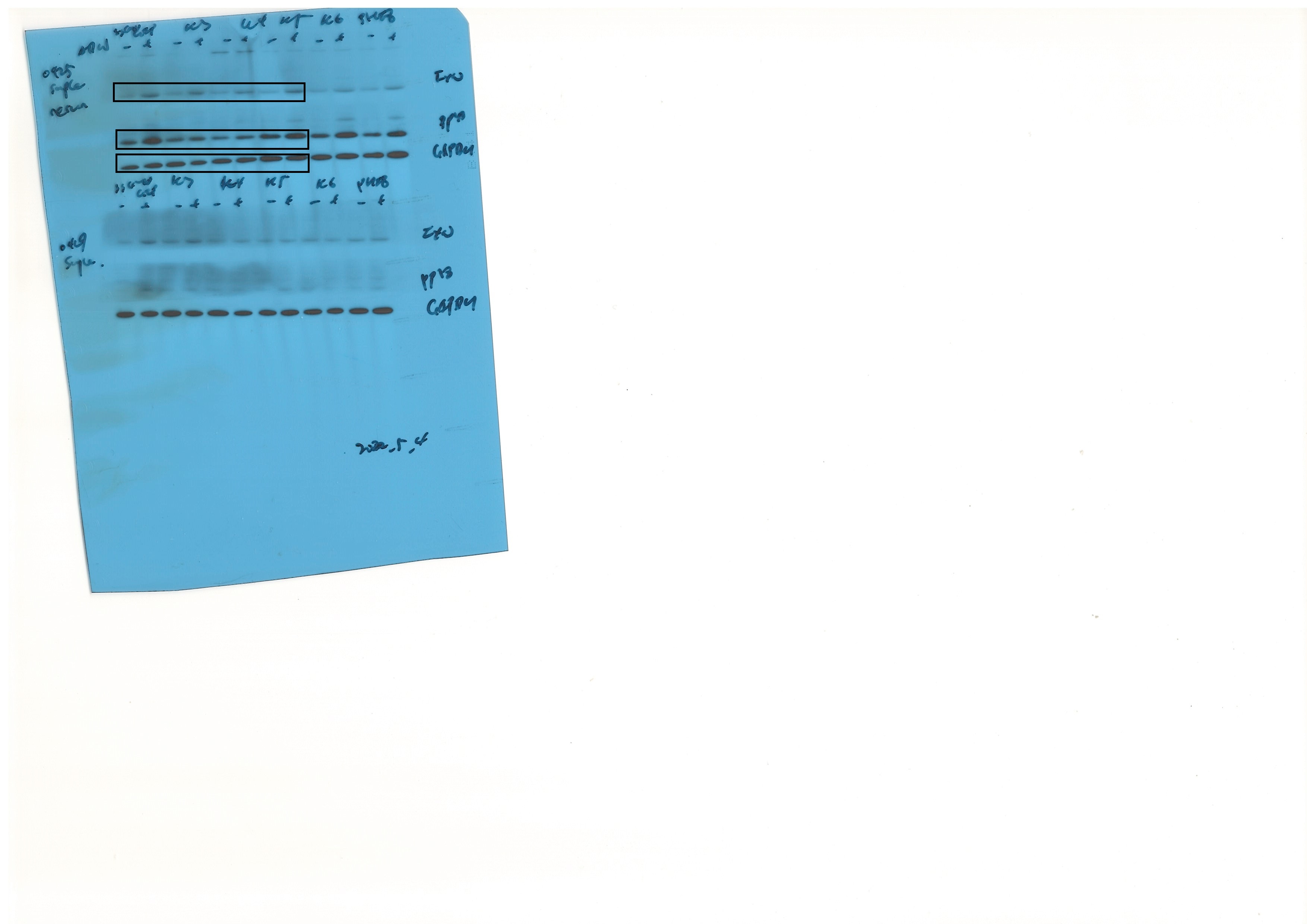

Supplement: Figure 3—figure supplement 1—source data 2. [file elife-93921-fig3-figsupp1-data2.zip › S4F.jpg]

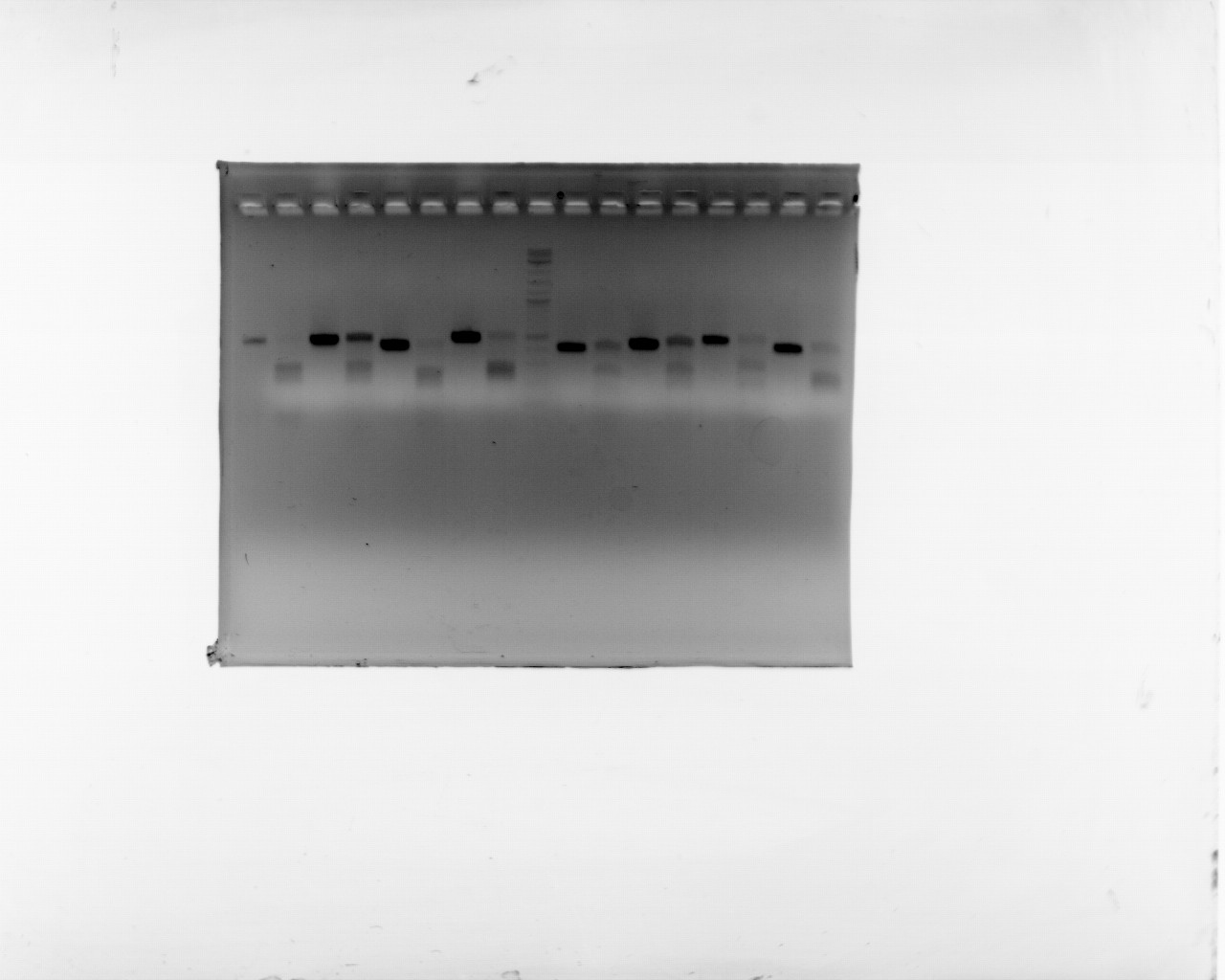

Supplement: Figure 3—figure supplement 2—source data 1. [file elife-93921-fig3-figsupp2-data1.zip › S5B.tif]

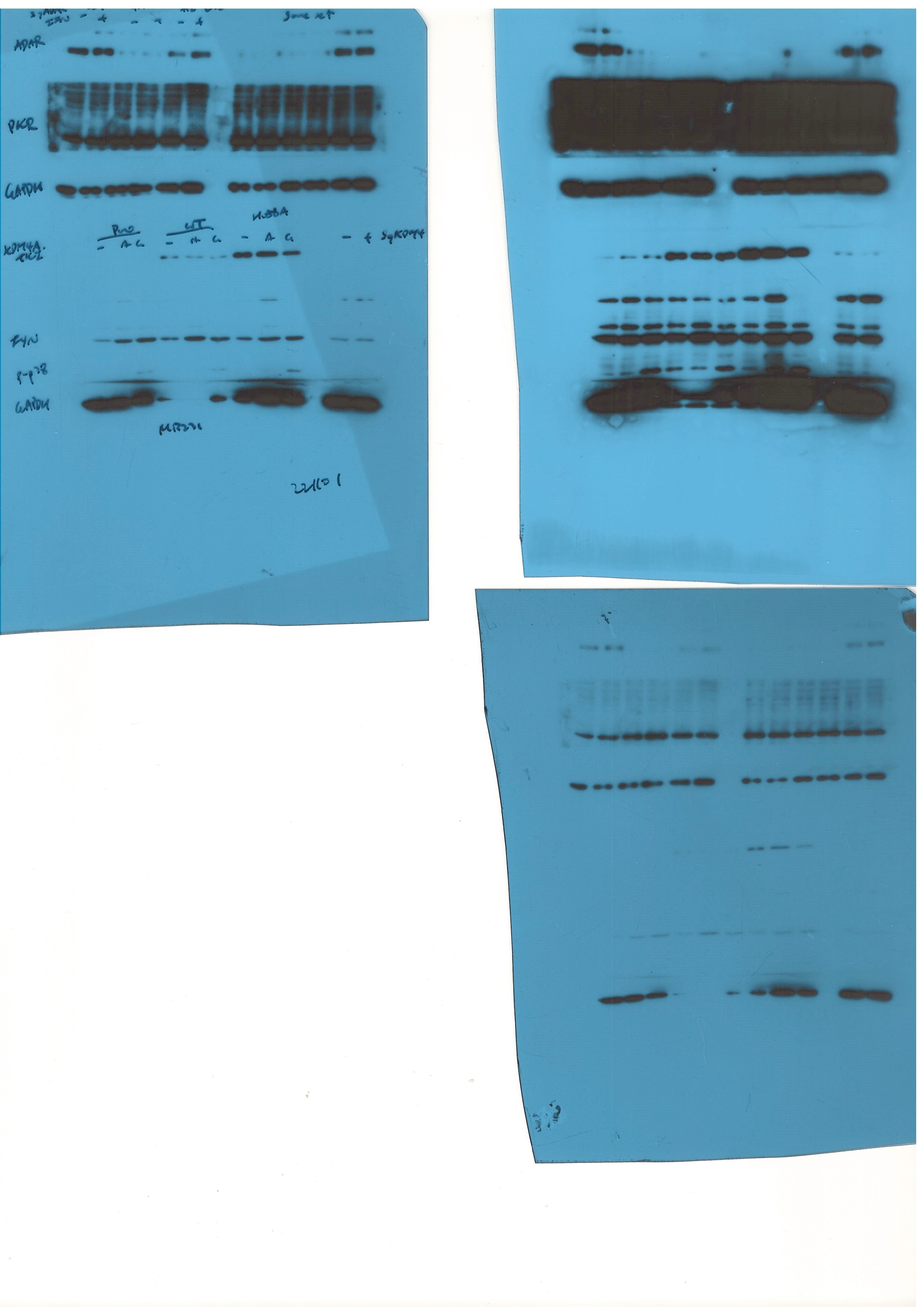

Supplement: Figure 3—figure supplement 2—source data 1. [file elife-93921-fig3-figsupp2-data1.zip › S5C.jpg]

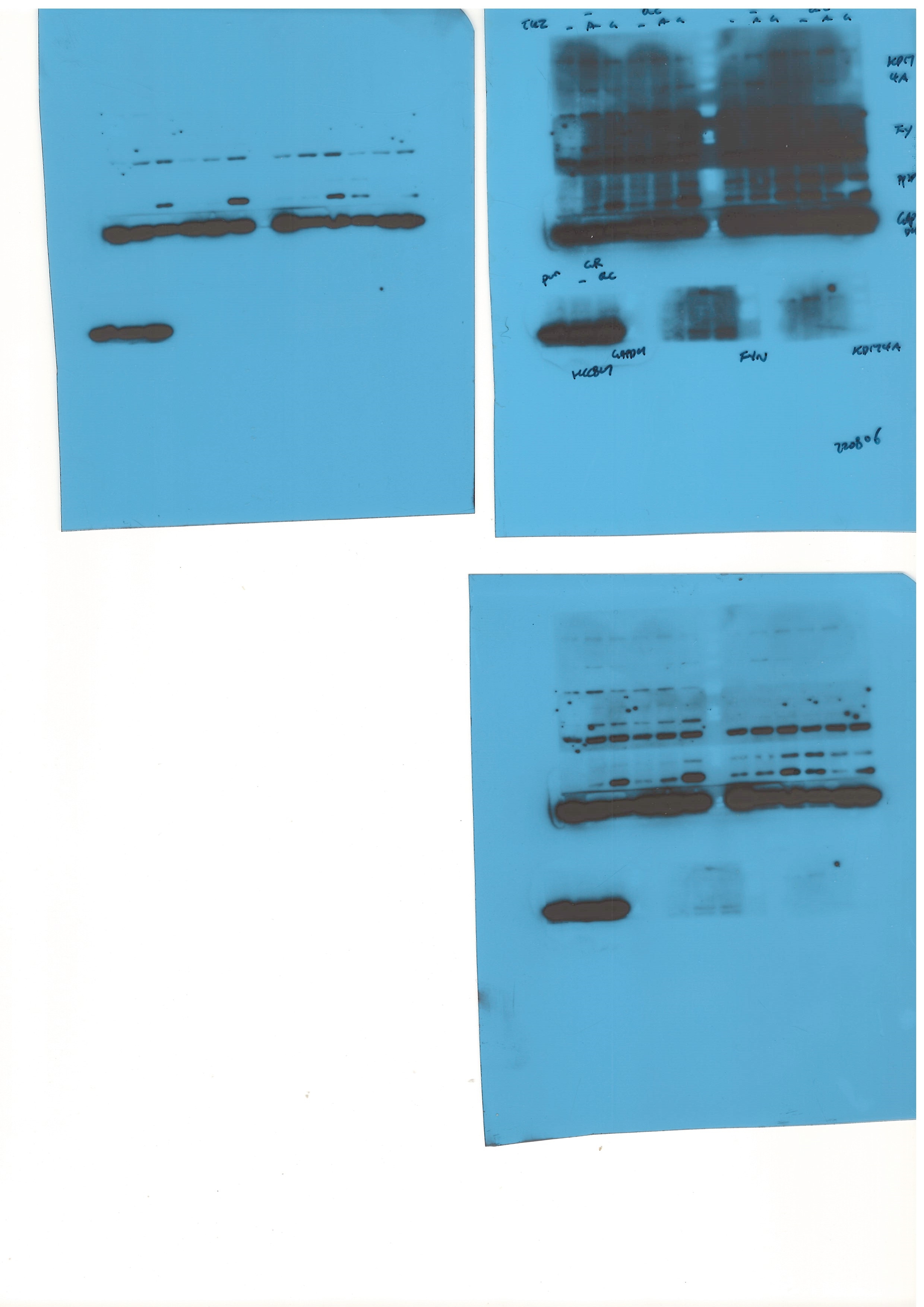

Supplement: Figure 3—figure supplement 2—source data 1. [file elife-93921-fig3-figsupp2-data1.zip › S5F-left.jpg]

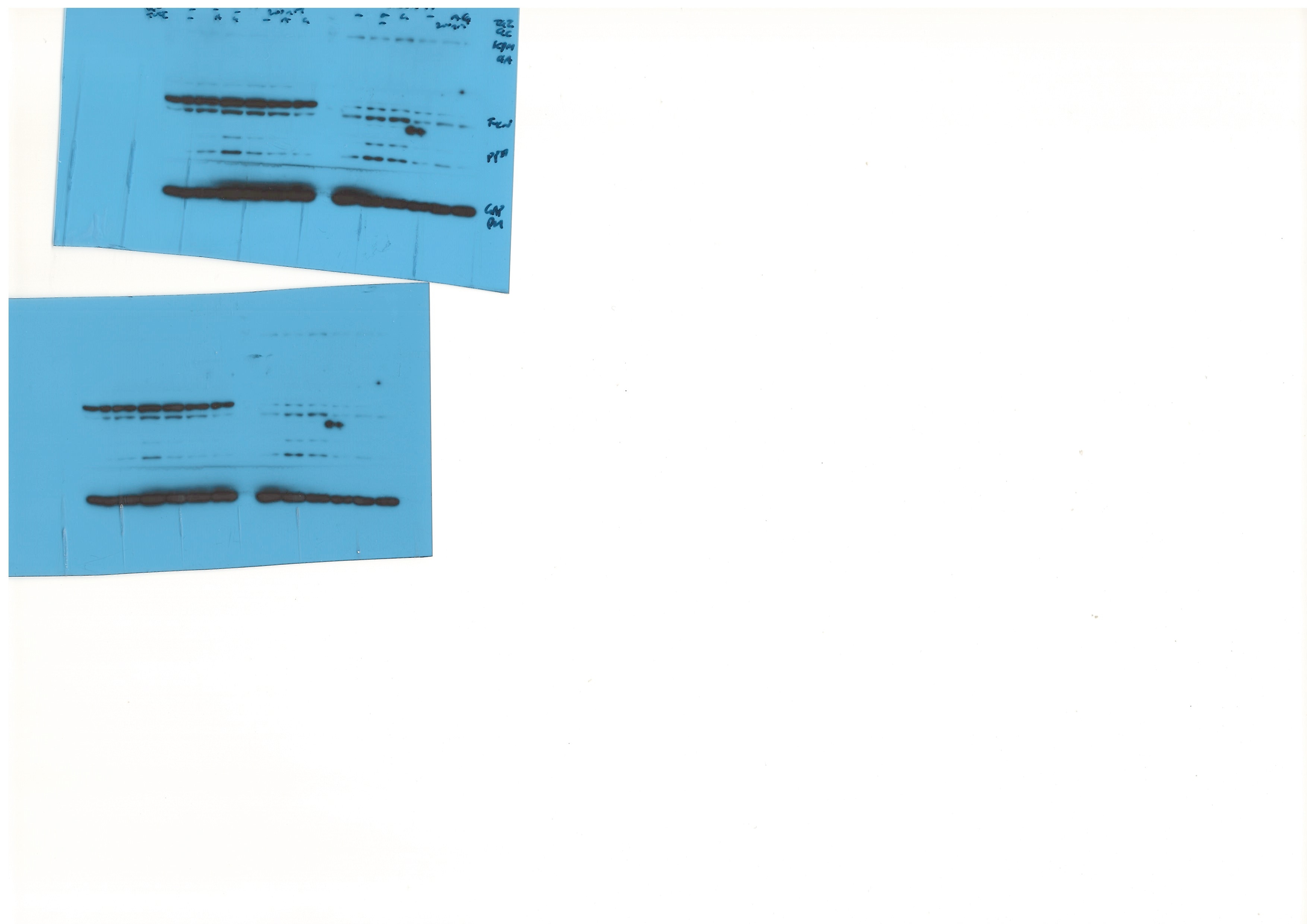

Supplement: Figure 3—figure supplement 2—source data 1. [file elife-93921-fig3-figsupp2-data1.zip › S5F-middle.jpg]

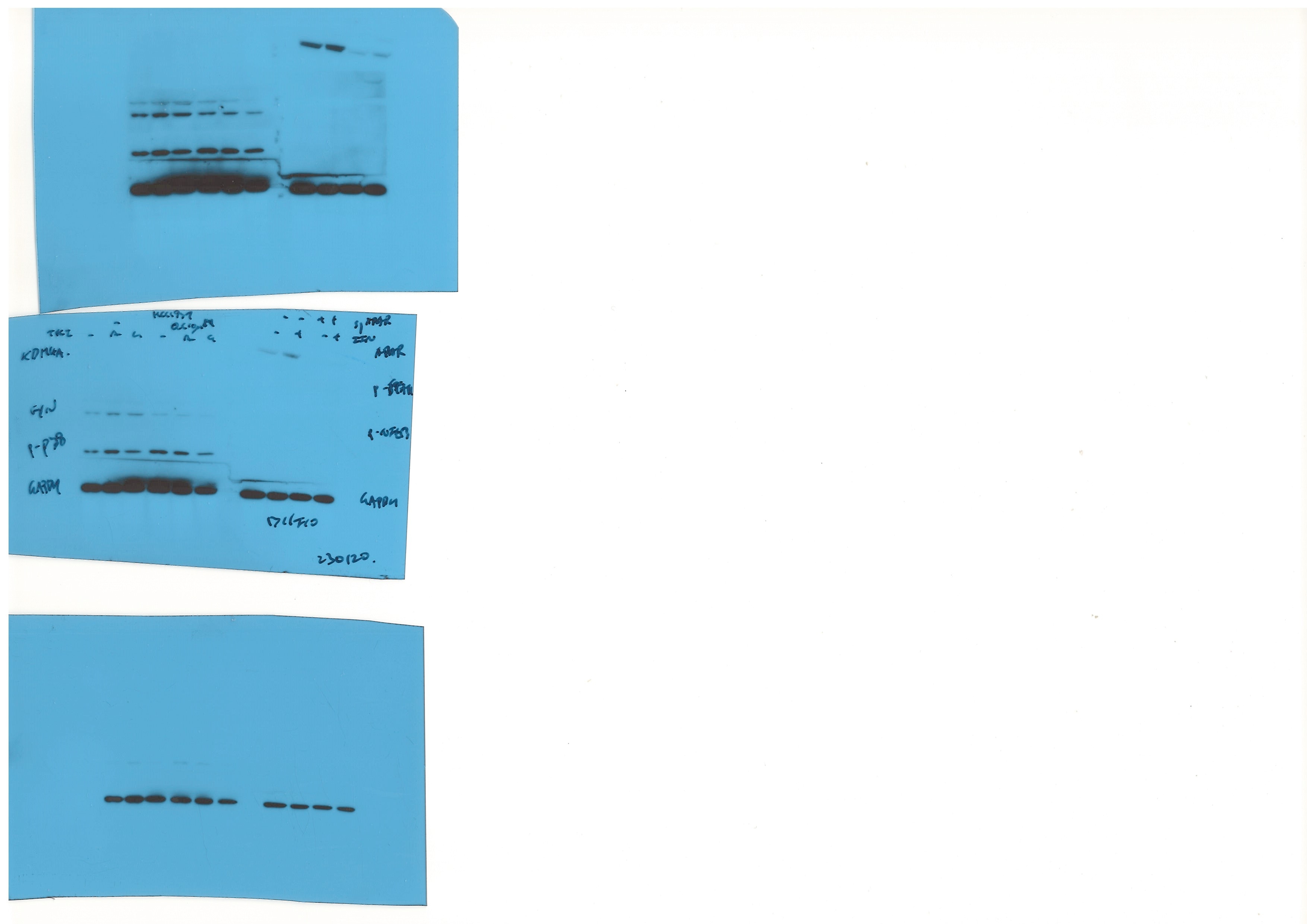

Supplement: Figure 3—figure supplement 2—source data 1. [file elife-93921-fig3-figsupp2-data1.zip › S5F-right.jpg]

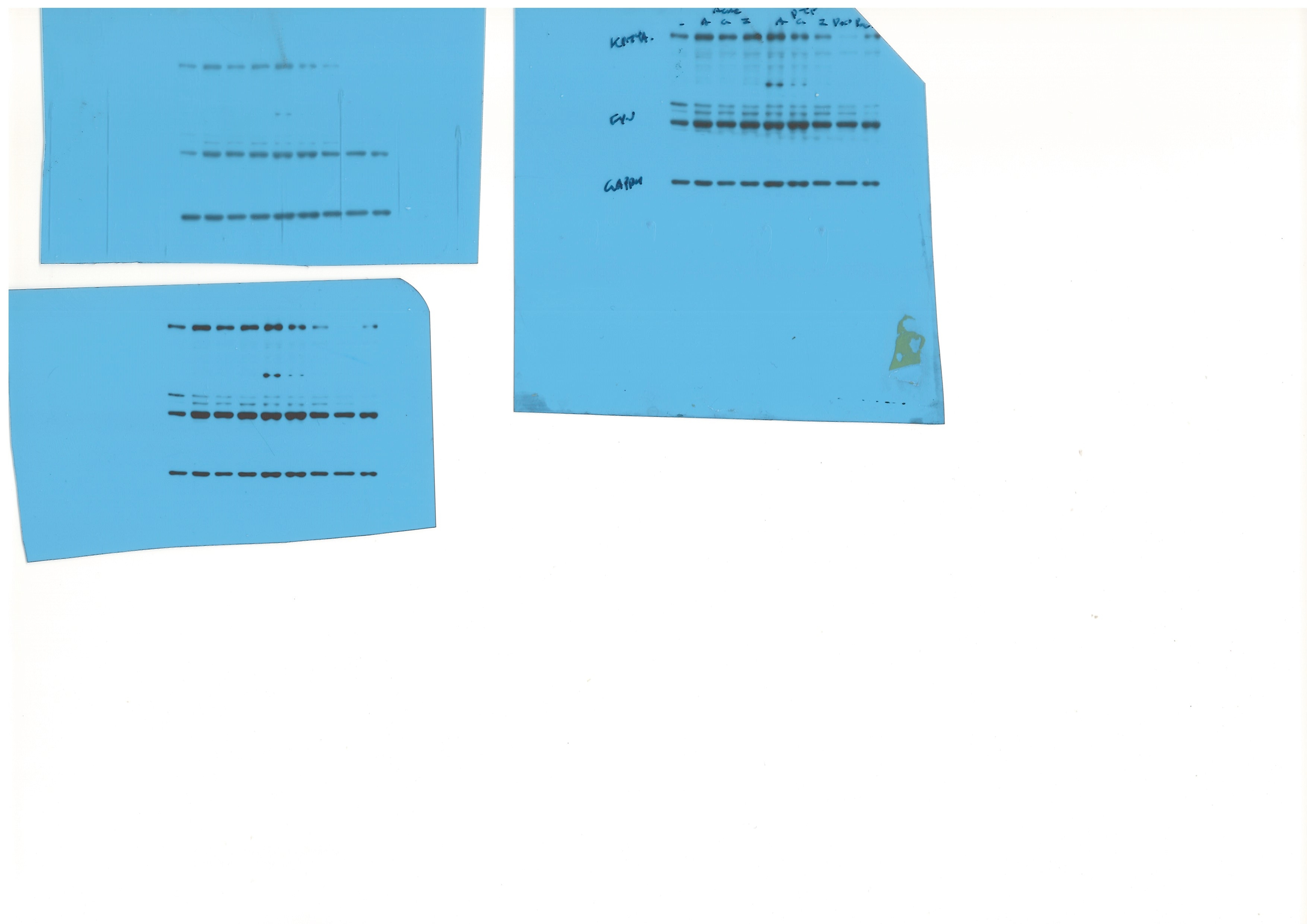

Supplement: Figure 5—source data 1. [file elife-93921-fig5-data1.zip › 5A.jpg]

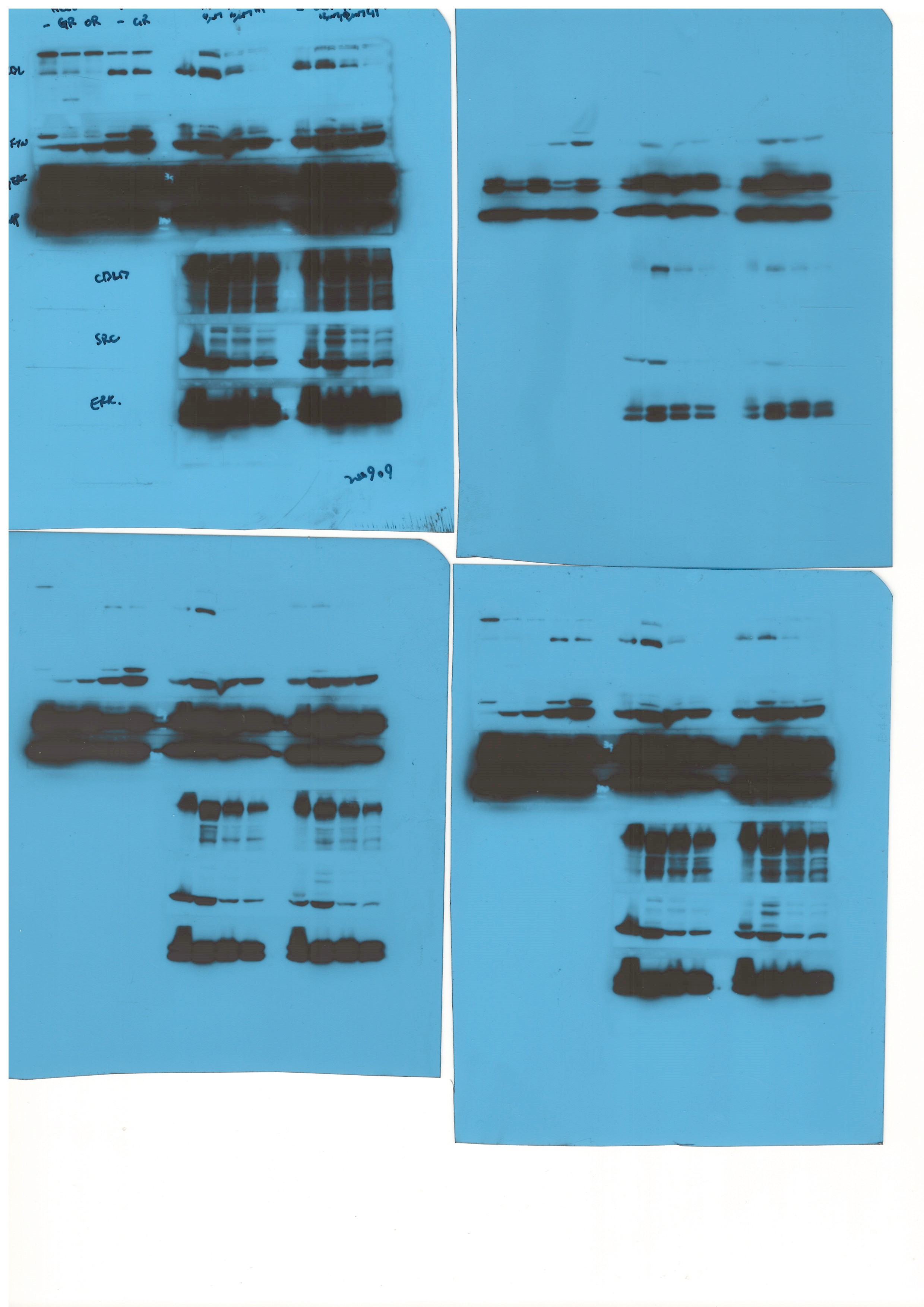

Supplement: Figure 5—source data 1. [file elife-93921-fig5-data1.zip › 5C.jpg]

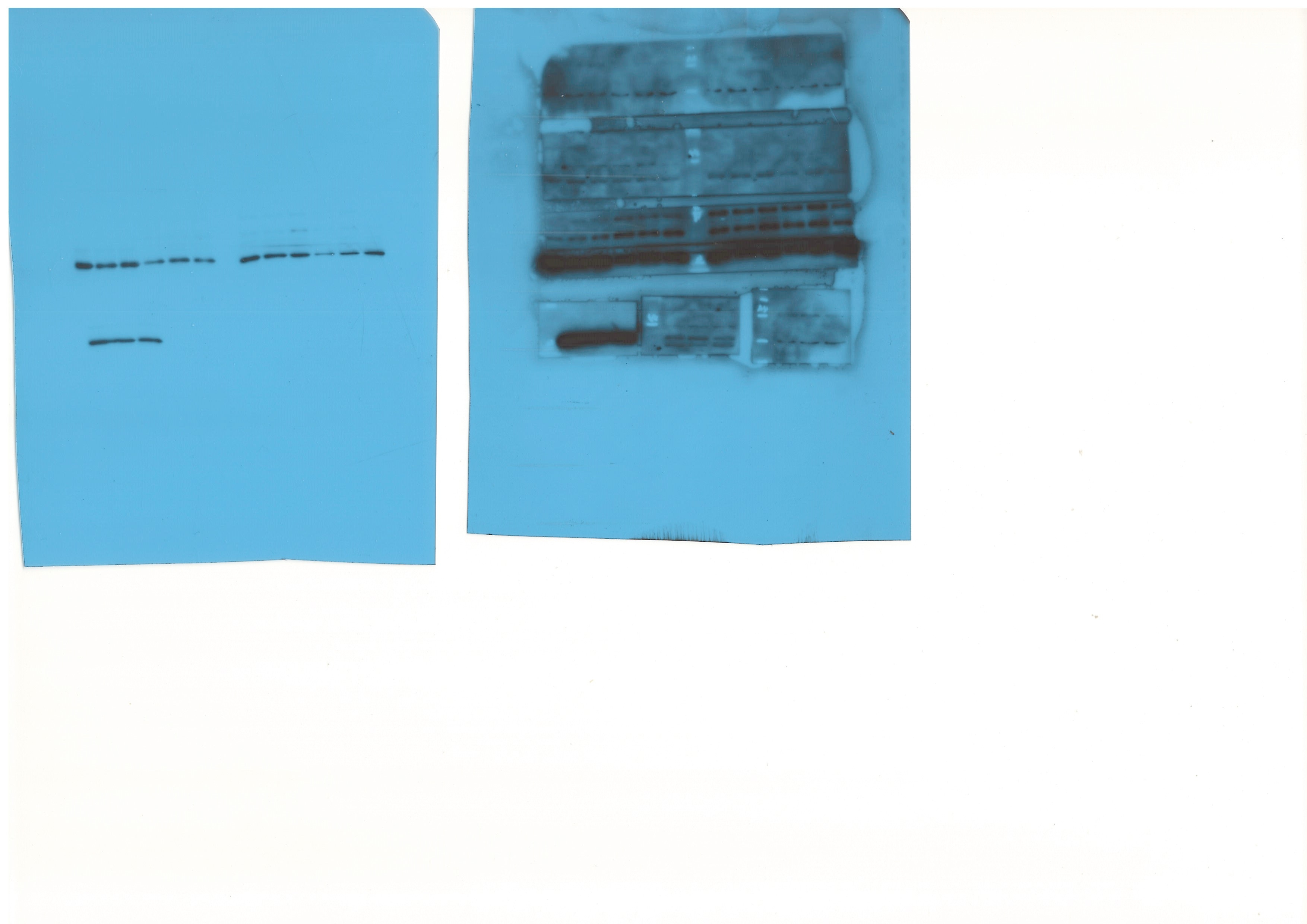

Supplement: Figure 5—source data 1. [file elife-93921-fig5-data1.zip › 5G.jpg]

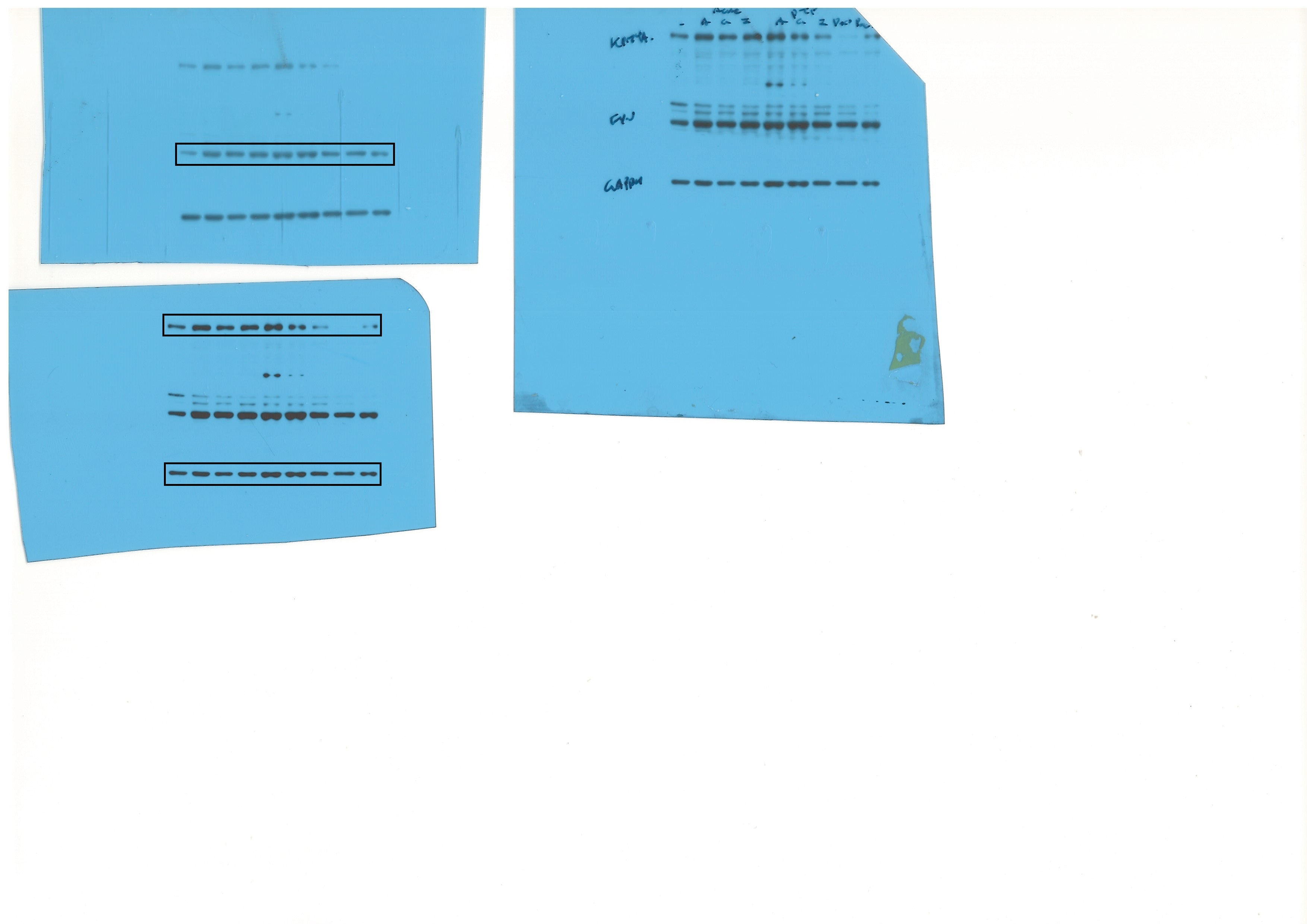

Supplement: Figure 5—source data 2. [file elife-93921-fig5-data2.zip › 5A.jpg]

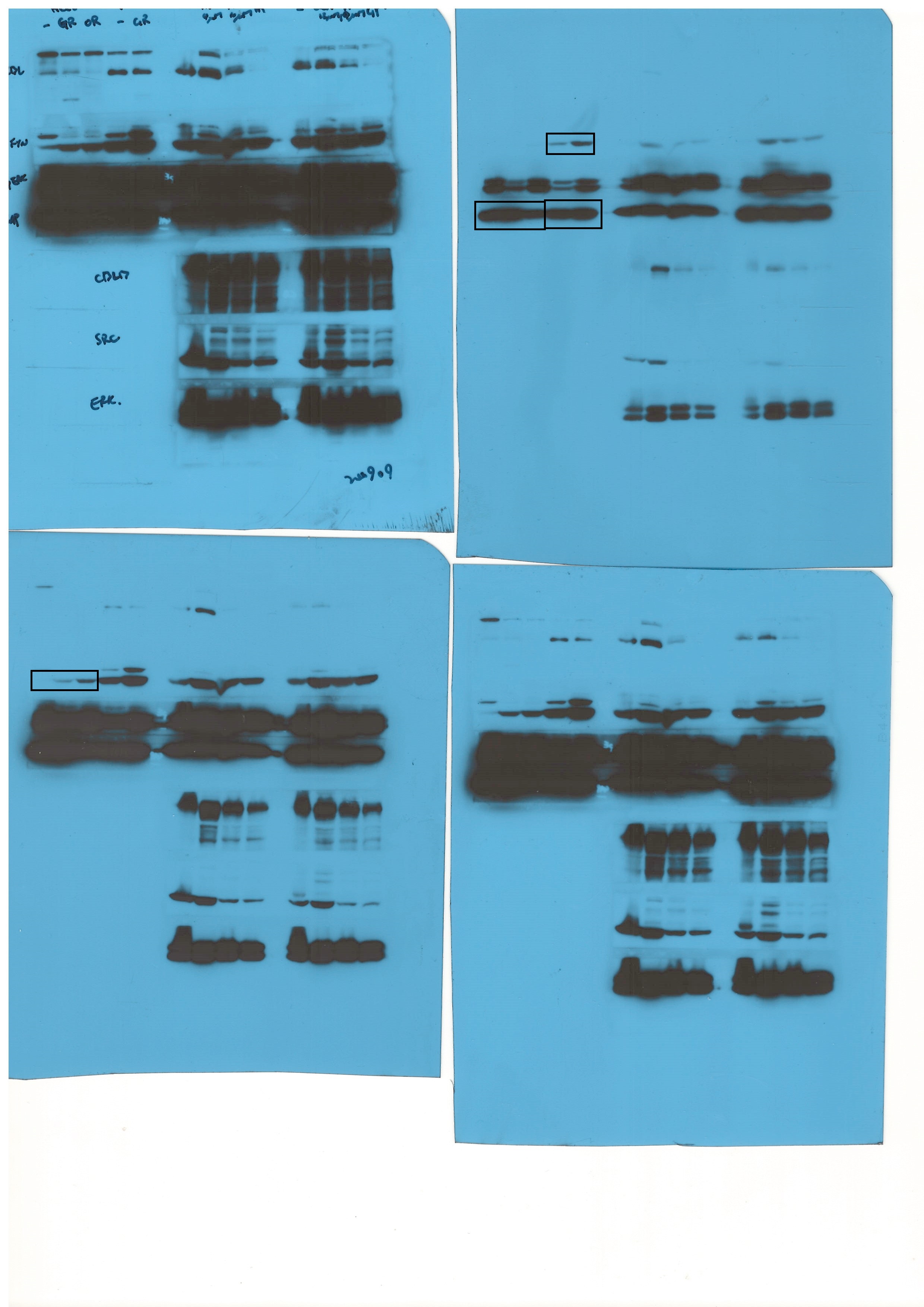

Supplement: Figure 5—source data 2. [file elife-93921-fig5-data2.zip › 5C.jpg]

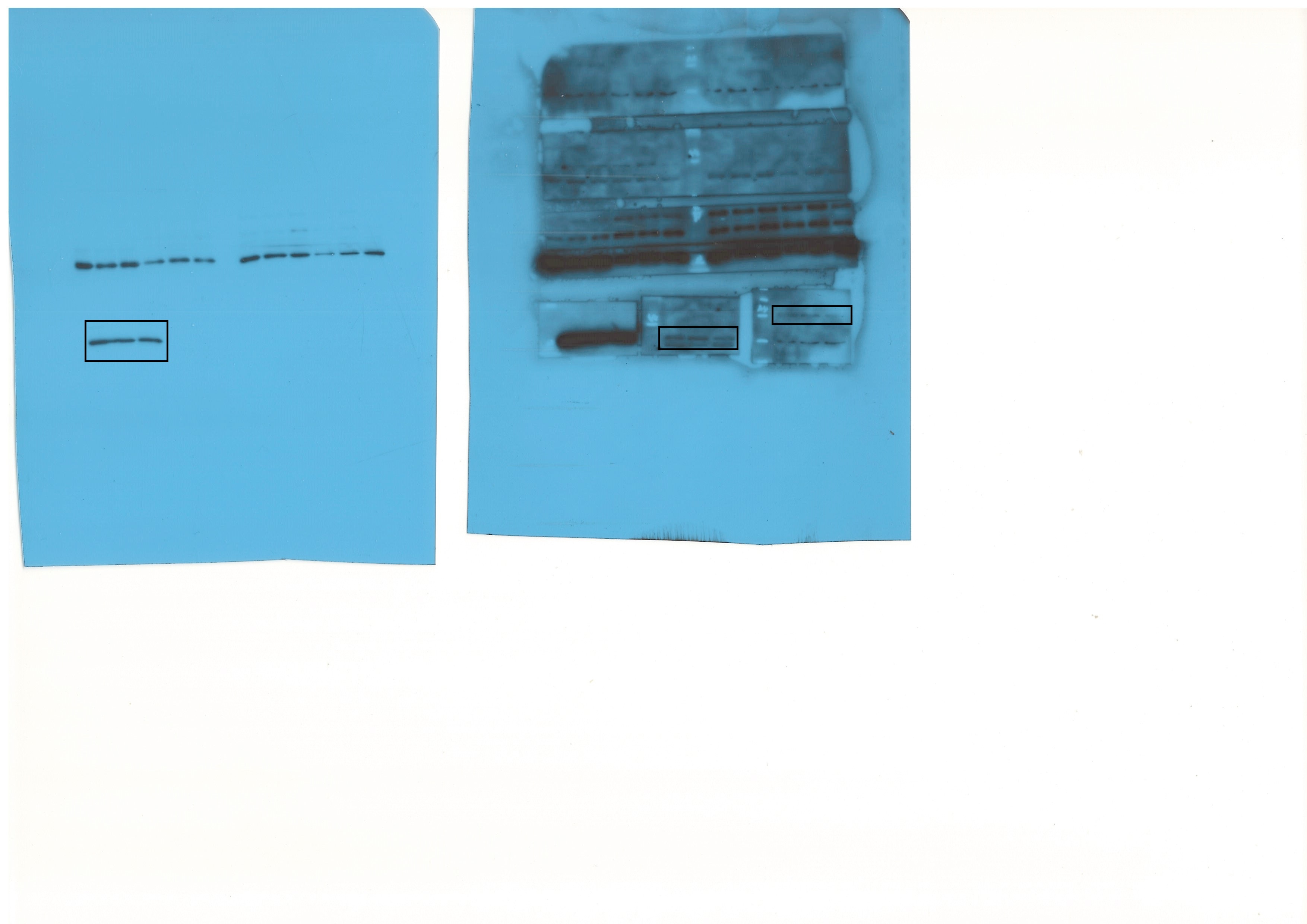

Supplement: Figure 5—source data 2. [file elife-93921-fig5-data2.zip › 5G.jpg]
